# Supplementary material for: Synthesis and Anti-Inflammatory Activity of N(2)-Arylindazol-3(2H)-One Derivatives: Copper-Promoted Direct N-Arylation via Chan–Evans–Lam Coupling
Source: Molecules. 2023 Sep 20;28(18):6706. doi: 10.3390/molecules28186706 (PMC10538006; doi:10.3390/molecules28186706)

Synthesis and Anti-Inflammatory Activity of *N*(2)-Arylindazol-3(2*H*)-One Derivatives: Copper-Promoted Direct *N*-Arylation via Chan–Evans–Lam Coupling

Kyungmin Kim [1,†], Jeong Ho Kim [1,†], Heejae Choi [1], Byeongno Lee [1], Jihyun Lee [2], Kang Min Ok [2], Tae Hoon Lee [1] and Hakwon Kim [1,*]

1 Department of Applied Chemistry, Global Center for Pharmaceutical Ingredient Materials, Kyung Hee University, Yongin-si, Gyeonggi 17104, Republic of Korea; sp10101@naver.com (K.K.); jeongho1333@gmail.com (J.H.K.); chlgmlwo96@naver.com (H.C.); bnlee@sogang.ac.kr (B.L.); thlee@khu.ac.kr (T.H.L.)

2 Department of Chemistry, Sogang University, Seoul 04107, Republic of Korea; jh_christina@naver.com (J.L.); kmok@sogang.ac.kr (K.M.O.)

* Correspondence: hwkim@khu.ac.kr; Tel.: +823-1201-2459

† These authors contributed equally to this work.

Keywords: Anti-inflammatory, cytotoxicity, indazol-3-ones, *N*-arylation, Chan-Evans-Lam coupling

Table of Contents

1. The General Procedure for the Synthesis of Compounds (S1j-S1m)
2. Biological Activity Raw Data
3. SCXRD (3a)
4. References
5. ^{1}H and ^{13}C NMR Spectra of Compounds

1. **The General Procedure for the Synthesis of Compounds (S1j-S1m).**

All chemical reagents were purchased from Sigma-Aldrich (St. Louis, MO, USA), Tokyo Chemical Industry (Tokyo, Japan), Alfa Aesar (Morecambe, UK), and Acros Organics (Brookline, MA, USA) and were used without further purification. All glassware was thoroughly dried in a convection oven. Reactions were monitored using thin-layer chromatography (TLC). Commercial TLC plates (silica gel 60 F_{254}, Merck Co., Rahway, NJ, USA) were developed and the spots were visualized under UV light at 254 or 365 nm. Silica gel column chromatography was performed with silica gel 60 (particle size 0.040−0.063 mm, Merck Co., Rahway, NJ, USA). Extra pure-grade solvents for column chromatography were purchased through Samchun Chemicals (Seoul, Republic of Korea) and Duksan Chemicals (Incheon, Republic of Korea). ^{1}H and ^{13}C NMR spectra were collected with a JEOL ECX-400 spectrometer (at 300 MHz for ^{1}H NMR and 75 MHz for ^{13}C NMR, Tokyo, Japan) and a JEOL JNM-ECZ400S (at 400 MHz for ^{1}H NMR and 100 MHz for ^{13}C NMR; Tokyo, Japan). ^{1}H NMR spectra chemical shifts were expressed in parts per million (ppm) downfield from tetramethylsilane [$Si(CH_3)_4$], and coupling constants were reported in Hertz (Hz). Splitting patterns are indicated as follows: s, singlet; d, doublet; t, triplet; and m, multiplet. ^{13}C NMR spectra were reported in ppm, referenced to chloroform-*d* and DMSO-d_6. Melting points (m.p.) were determined on a Barnstead Electrothermal 9100 instrument (Essex, UK) and were uncorrected. High resolution mass spectrometry (HRMS) spectra were recorded using a Jeol (JMS-700; Tokyo, Japan).

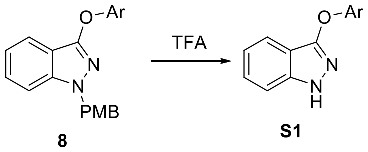

8 **S1**

N(1)-PMB protected *O*(3)-aryl-substituted indazoles (**8**, 0.15 mmol) were dissolved in TFA (2.3 mL). The mixture was stirred at 60 °C for 1 hour. After the reaction was complete, the mixture was concentrated under vacuum and subsequently purified through silica gel flash column chromatography to obtain pure solid products. (**S1j-S1m**).

S1j, Yield = 99%, white solid, 3-(4-nitrophenoxy)-1*H*-indazole, m.p.: 146-148 °C. ^{1}H NMR (400 MHz, DMSO-d_6) δ 8.28 (d, *J* = 6.8 Hz, 2H), 7.56 (d, *J* = 8.4 Hz, 1H), 7.49 (d, *J* = 8.0 Hz, 1H), 7.44 (t, *J* = 7.2 Hz, 1H), 7.33 (d, *J* = 6.8 Hz, 2H), 7.13 (t, *J* = 7.2 Hz, 1H). ^{13}C NMR (100 MHz, $CDCl_3$) δ 161.49, 153.05, 143.47, 142.02, 128.29, 125.80, 121.43, 119.33, 117.75, 113.01, 110.35. HRMS (EI) calcd for $C_{13}H_9N_3O_3$ $(M)^+$ 255.0644, found 255.0646.

S1k, Yield = 96%, white solid, 1-(4-(1*H*-indazol-3-yloxy)phenyl)ethanone, m.p.: 140-142 °C. ^{1}H NMR (400 MHz, DMSO-d_6) δ 7.95 (d, *J* = 6.8 Hz, 2H), 7.49 (d, *J* = 8.8 Hz, 1H), 7.39 (d, *J* = 8.8 Hz, 1H), 7.38 (t, *J* = 8.0 Hz, 1H), 7.17 (d, *J* = 6.8 Hz, 2H), 7.06 (t, *J* = 8.0 Hz, 1H), 2.51 (s, 3H). ^{13}C NMR (100 MHz, DMSO-d_6) δ 196.52,

160.49, 151.60, 141.56, 132.17, 130.62, 127.29, 120.59, 118.66, 116.76, 111.97, 110.96, 26.62. HRMS (EI) calcd for $C_{15}H_{12}N_2O_2$ $(M)^+$ 252.0899, found 252.0897.

S1l, Yield = 96%, white solid, 4-(1*H*-indazol-3-yloxy)benzonitrile, m.p.: 159-161 °C. ^{1}H NMR (400 MHz, DMSO-d_6) δ 7.87 (d, *J* = 6.8 Hz, 2H), 7.54 (d, *J* = 8.8 Hz, 1H), 7.46 (d, *J* = 8.8 Hz, 1H), 7.43 (t, *J* = 7.2 Hz, 1H), 7.29 (d, *J* = 6.8 Hz, 2H), 7.12 (t, *J* = 7.2 Hz, 1H). ^{13}C NMR (100 MHz, $CDCl_3$) δ 159.93, 153.14, 142.01, 134.06, 128.25, 121.34, 119.40, 118.68, 118.40, 113.03, 110.31, 107.04. HRMS (EI) calcd for $C_{14}H_9N_3O$ $(M)^+$ 235.0746, found 235.0745.

S1m, Yield = 98%, white solid, 3-(4-(trifluoromethyl)phenoxy)-1*H*-indazole, m.p.: 81-83 °C. ^{1}H NMR (400 MHz, DMSO-d6) δ 7.75 (d, *J* = 8.8 Hz, 2H), 7.53 (d, *J* = 8.4 Hz, 1H), 7.46 (d, *J* = 8.0 Hz, 1H), 7.42 (t, *J* = 8.4 Hz, 1H), 7.31 (d, *J* = 8.4 Hz, 2H), 7.11 (d, *J* = 8.0 Hz, 1H). ^{13}C NMR (100 MHz, $CDCl_3$) δ 159.05, 153.84, 142.06, 128.10, 127.09, 127.06, 126.00, 125.68, 125.45, 122.75, 121.09, 119.59, 118.09, 113.02, 110.24. HRMS (EI) calcd for $C_{14}H_9F_3N_2O$ $(M)^+$ 278.0667, found 278.0664.

2. Biological Activity Raw Data

Figure S1. Cell viability assay (LD 50) of **3** and **5**

3a

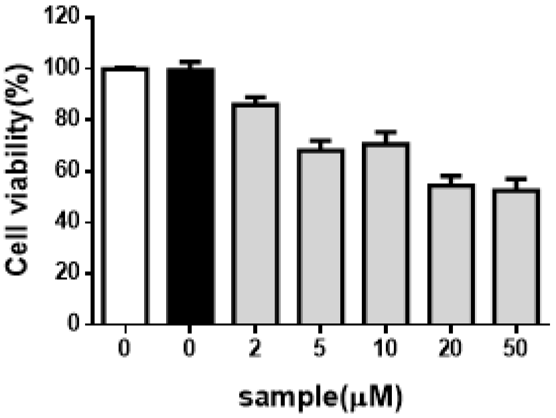

3b

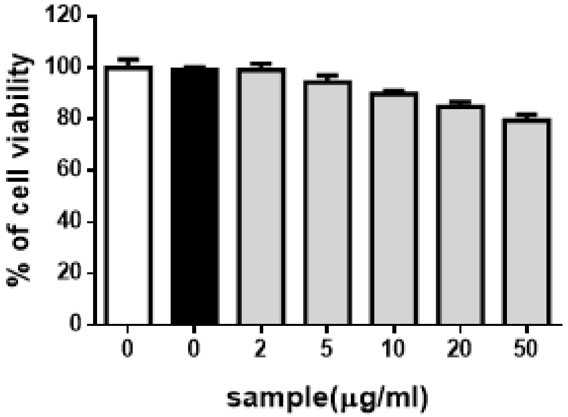

3c

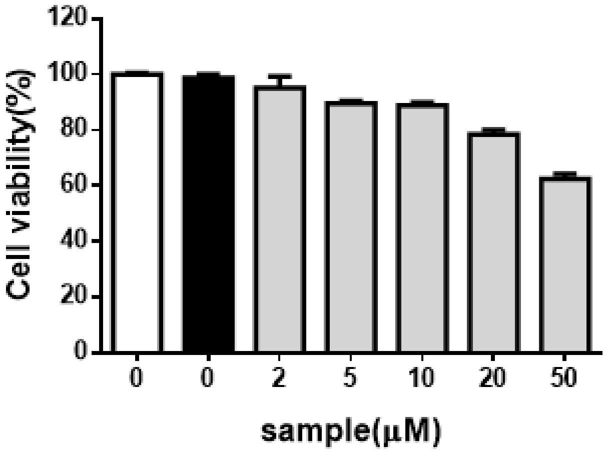

3d

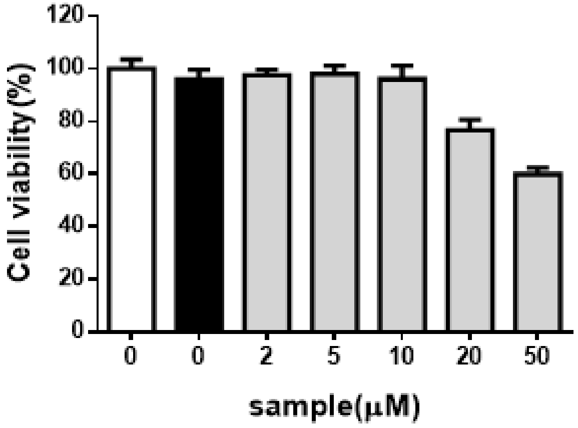

Cell viability(%)
120
100
80
60
40
20
0
0
0
2
5
10
20
50
sample(μM)

3e

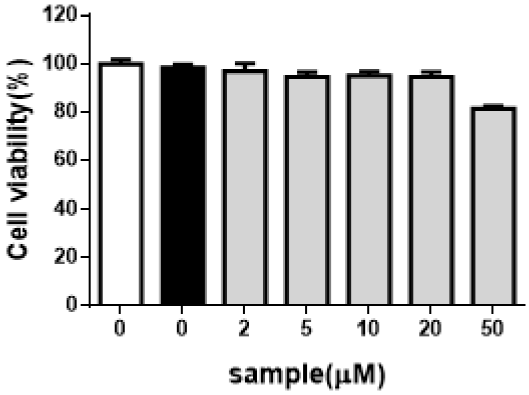

Cell viability(%)
120
100
80
60
40
20
0
0
0
2
5
10
20
50
sample(μM)

3f

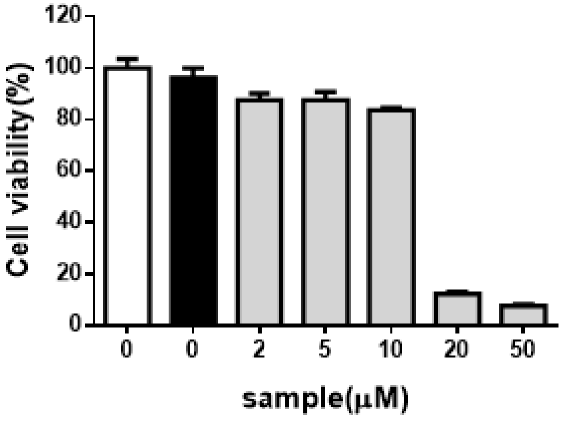

Cell viability(%)
120
100
80
60
40
20
0
0
0
2
5
10
20
50
sample(μM)

3g

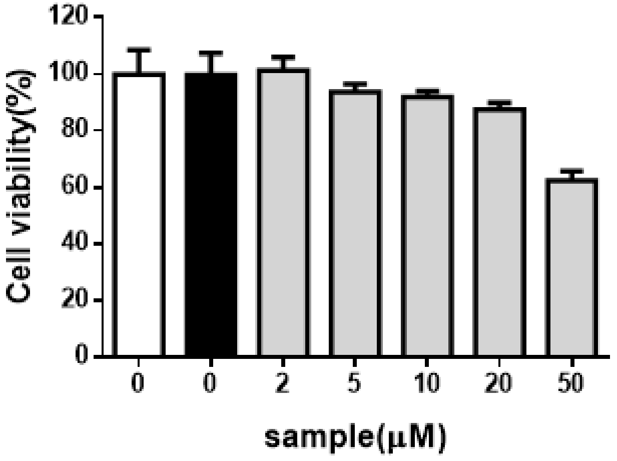
Cell viability(%)
120
100
80
60
40
20
0
0
0
2
5
10
20
50
sample(μM)

3h

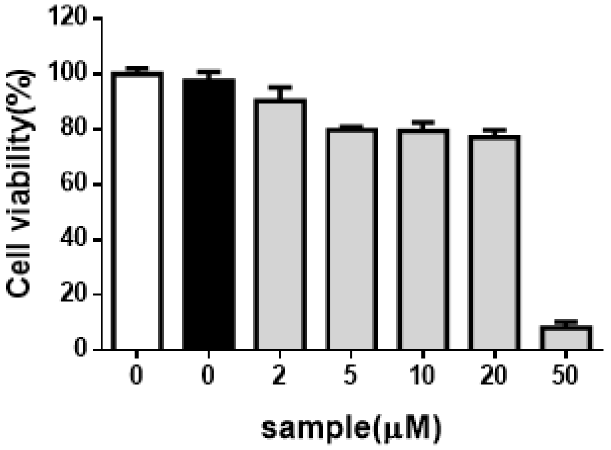
Cell viability(%)
120
100
80
60
40
20
0
0
0
2
5
10
20
50
sample(μM)

3i

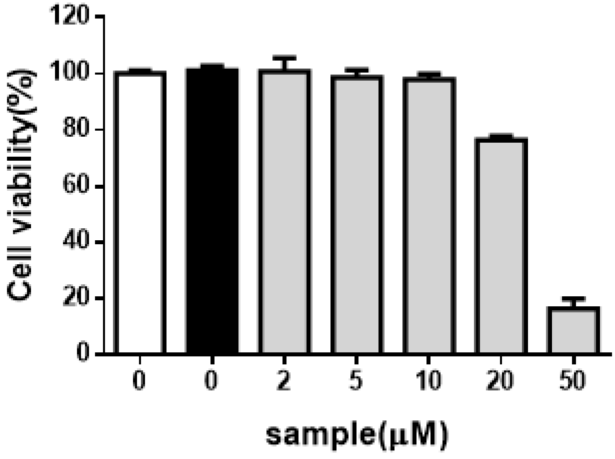
Cell viability(%)
120
100
80
60
40
20
0
0
0
2
5
10
20
50
sample(μM)

3j

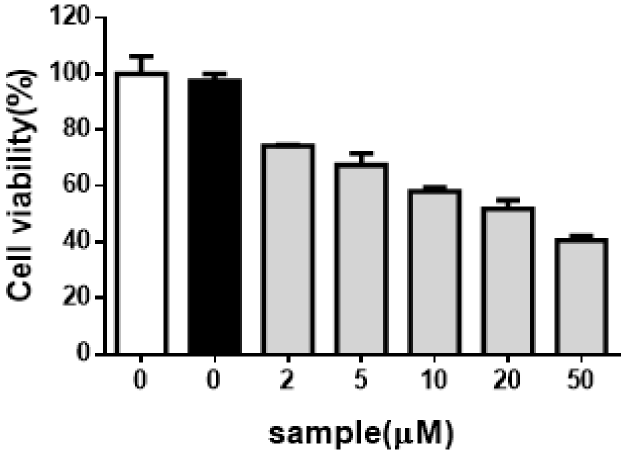
Cell viability(%)
0
20
40
60
80
100
120
0 0 2 5 10 20 50
sample(μM)

3k

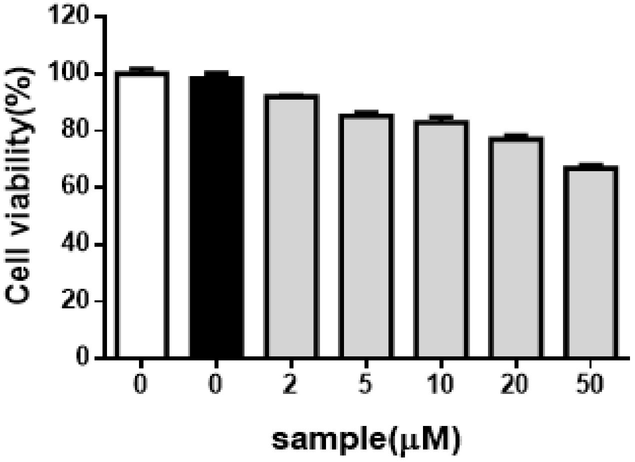
Cell viability(%)
0
20
40
60
80
100
120
0 0 2 5 10 20 50
sample(μM)

3l

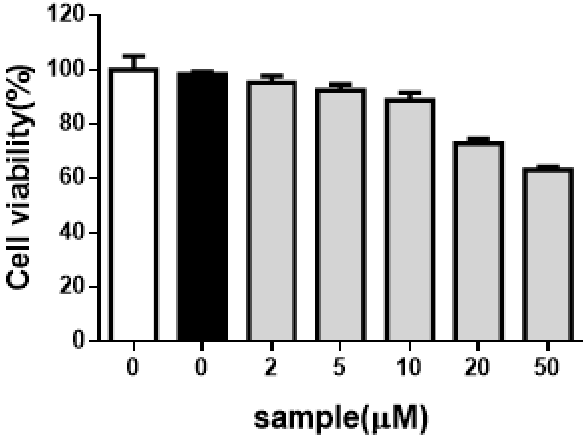
Cell viability(%)
0
20
40
60
80
100
120
0 0 2 5 10 20 50
sample(μM)

3m

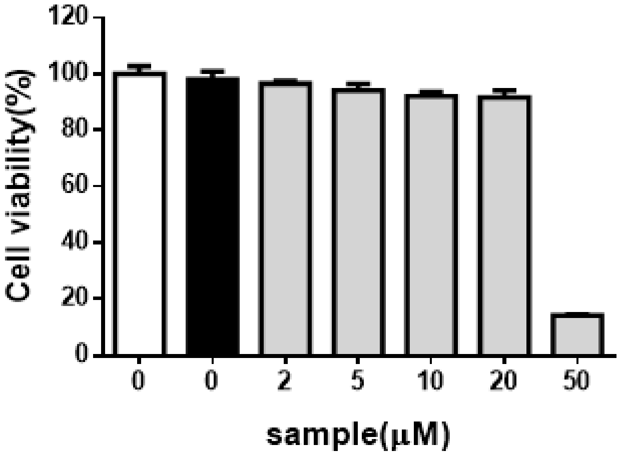
Cell viability(%)
120
100
80
60
40
20
0
0
0
2
5
10
20
50
sample(μM)

3n

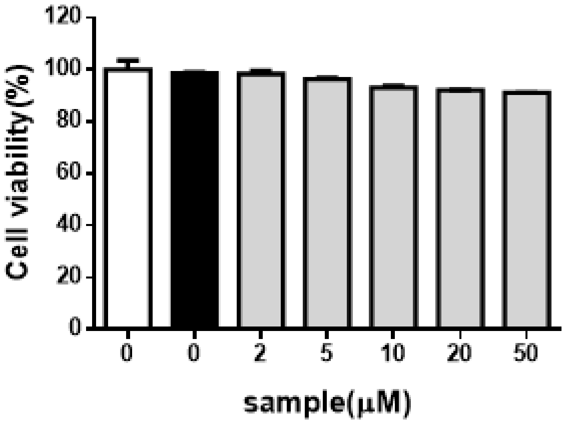
Cell viability(%)
120
100
80
60
40
20
0
0
0
2
5
10
20
50
sample(μM)

3o

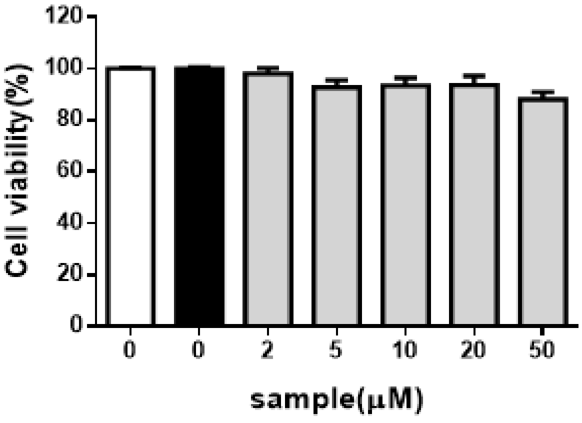
Cell viability(%)
120
100
80
60
40
20
0
0
0
2
5
10
20
50
sample(μM)

5a

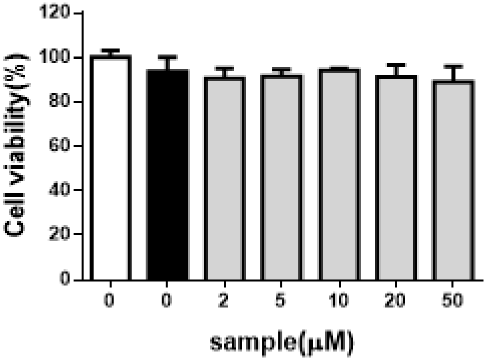
Cell viability(%)
0
20
40
60
80
100
120
0
0
2
5
10
20
50
sample(μM)

5b

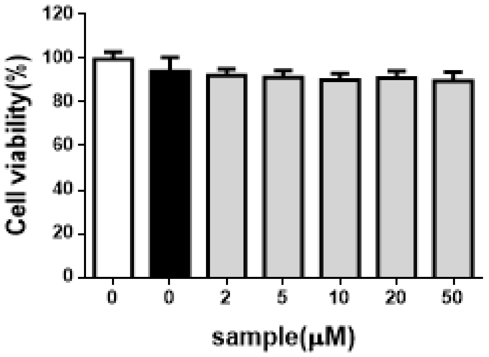
Cell viability(%)
0
20
40
60
80
100
120
0
0
2
5
10
20
50
sample(μM)

5c

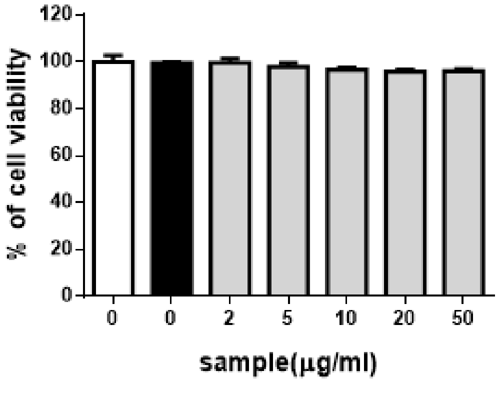
% of cell viability
0
20
40
60
80
100
120
0
0
2
5
10
20
50
sample(μg/ml)

5d

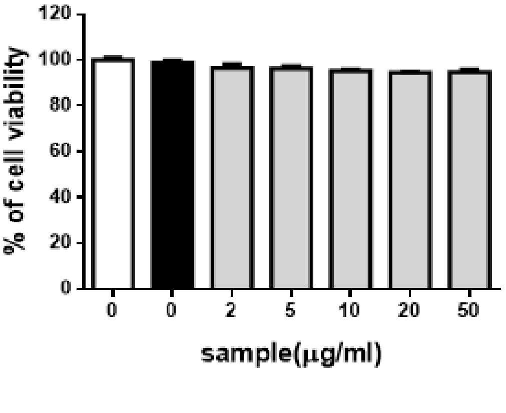
% of cell viability
0
20
40
60
80
100
120
0
0
2
5
10
20
50
sample(μg/ml)

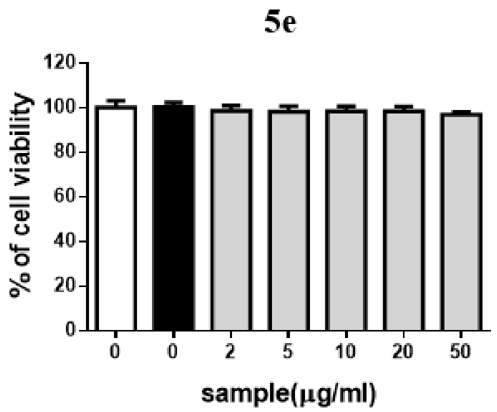
5e
120
100
80
60
40
20
0
% of cell viability
0
0
2
5
10
20
50
sample(μg/ml)

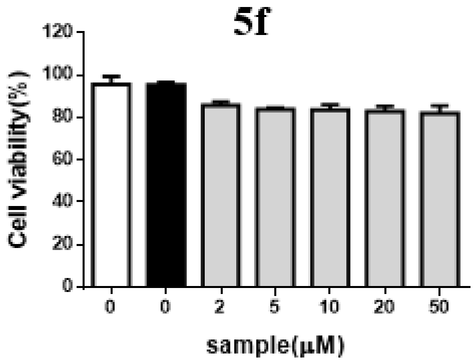
5f
120
100
80
60
40
20
0
Cell viability(%)
0
0
2
5
10
20
50
sample(μM)

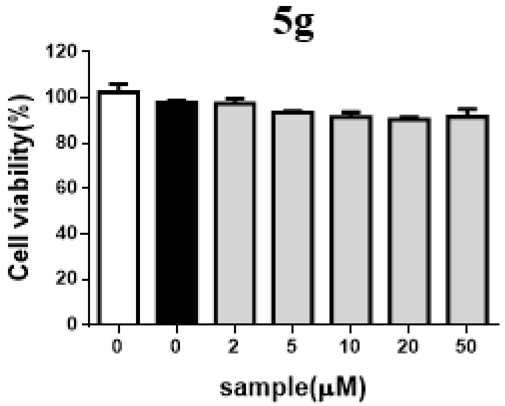
5g
120
100
80
60
40
20
0
Cell viability(%)
0
0
2
5
10
20
50
sample(μM)

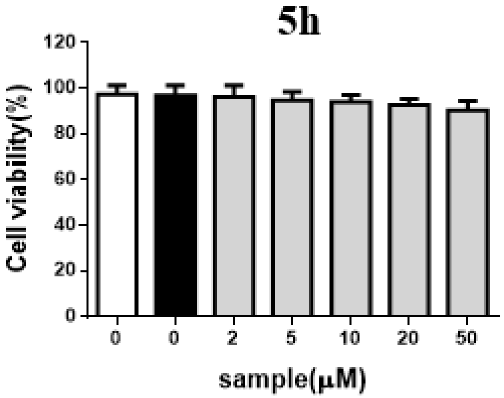
5h
120
100
80
60
40
20
0
Cell viability(%)
0
0
2
5
10
20
50
sample(μM)

5i

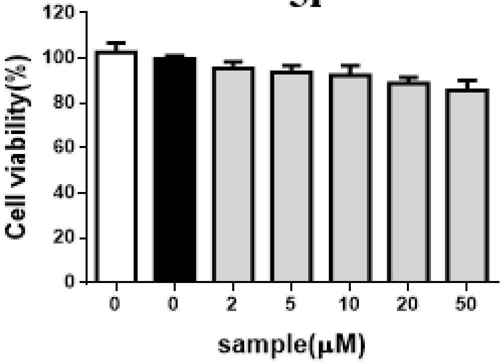
Cell viability(%)
0
20
40
60
80
100
120
0 0 2 5 10 20 50
sample(μM)

5j

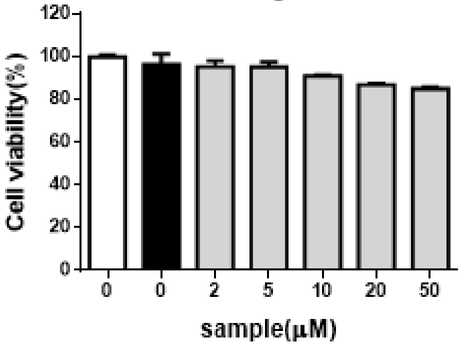
Cell viability(%)
0
20
40
60
80
100
120
0 0 2 5 10 20 50
sample(μM)

5k

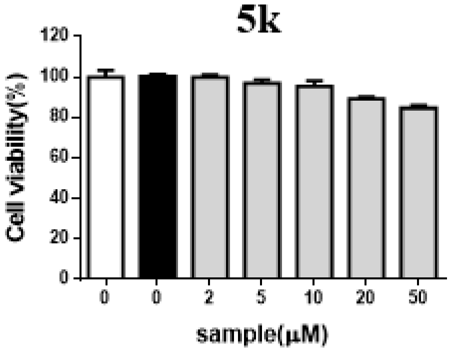
Cell viability(%)
0
20
40
60
80
100
120
0 0 2 5 10 20 50
sample(μM)

5l

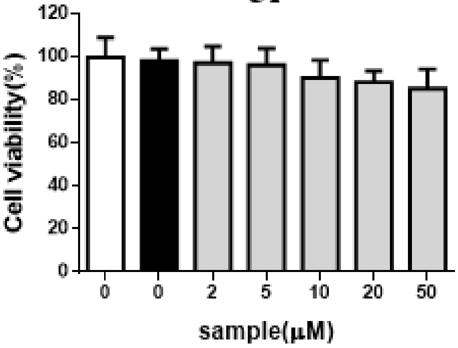
Cell viability(%)
0
20
40
60
80
100
120
0 0 2 5 10 20 50
sample(μM)

5m

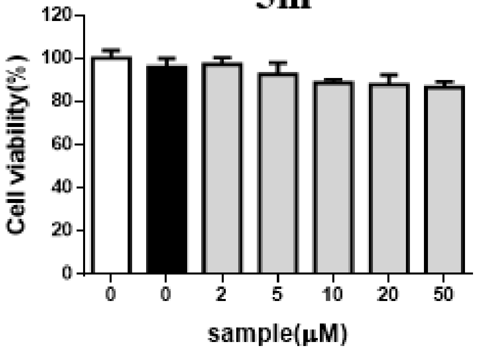

5n

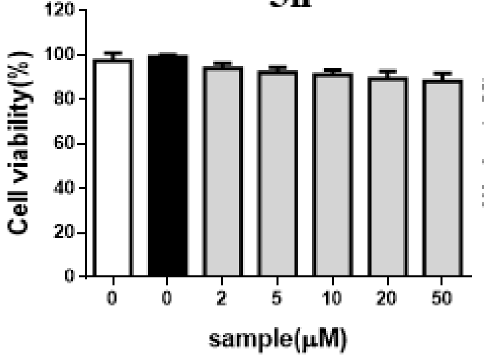

5o

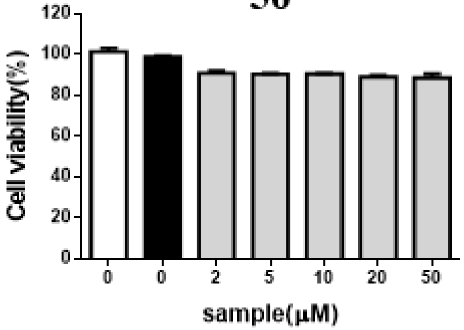

Figure S2. Nitric oxide inhibition assay (IC_{50}) of **5**.

5a

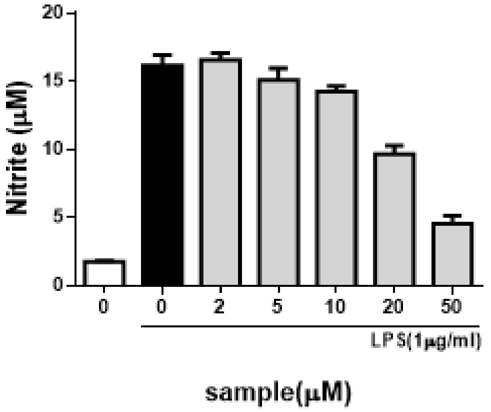
Nitrite (μM)
0
5
10
15
20
0
0
2
5
10
20
50
LPS(1μg/ml)
sample(μM)

5b

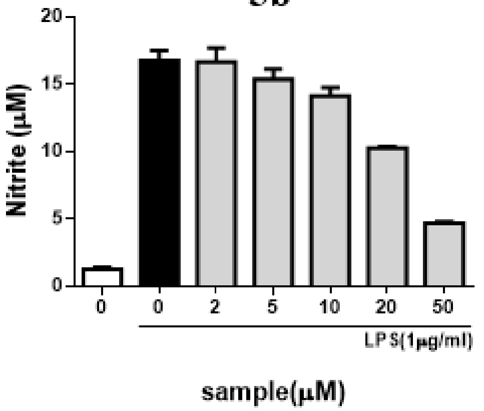
Nitrite (μM)
0
5
10
15
20
0
0
2
5
10
20
50
LPS(1μg/ml)
sample(μM)

5c

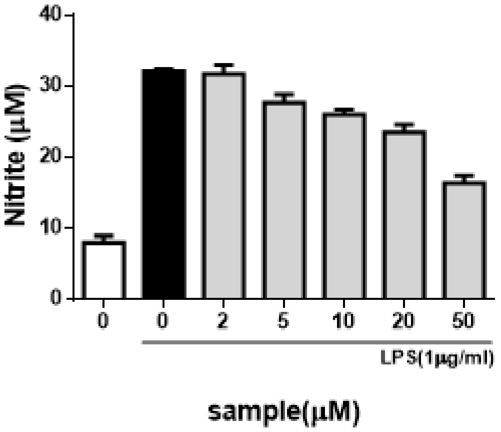
Nitrite (μM)
0
10
20
30
40
0
0
2
5
10
20
50
LPS(1μg/ml)
sample(μM)

5d

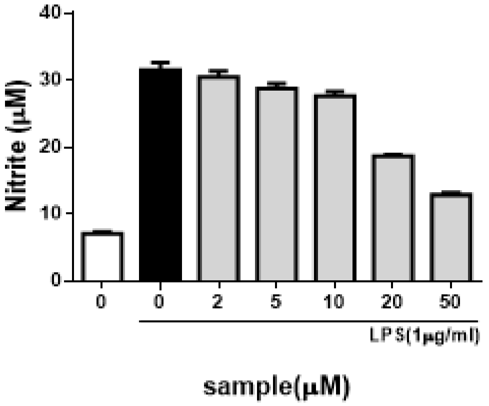
Nitrite (μM)
40
30
20
10
0
0
0
2
5
10
20
50
LPS(1μg/ml)
sample(μM)

5e

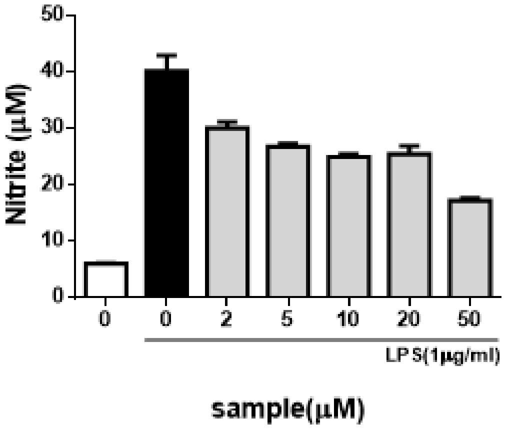
Nitrite (μM)
50
40
30
20
10
0
0
0
2
5
10
20
50
LPS(1μg/ml)
sample(μM)

5f

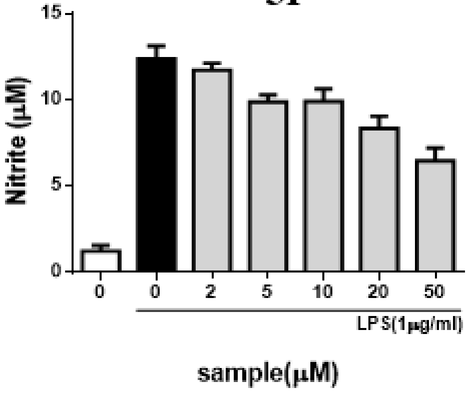
Nitrite (μM)
15
10
5
0
0
0
2
5
10
20
50
LPS(1μg/ml)
sample(μM)

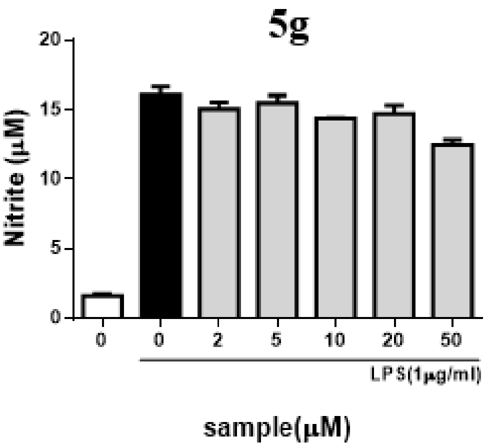
5g
Nitrite (μM)
20
15
10
5
0
0
0
2
5
10
20
50
LPS(1μg/ml)
sample(μM)

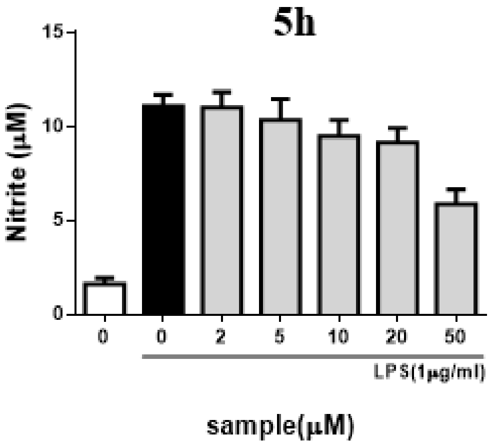
5h
Nitrite (μM)
15
10
5
0
0
0
2
5
10
20
50
LPS(1μg/ml)
sample(μM)

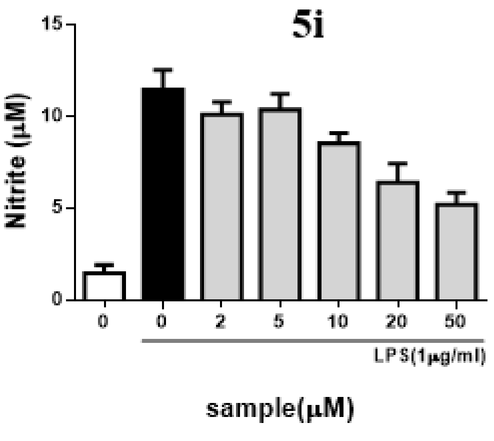
5i
Nitrite (μM)
15
10
5
0
0
0
2
5
10
20
50
LPS(1μg/ml)
sample(μM)

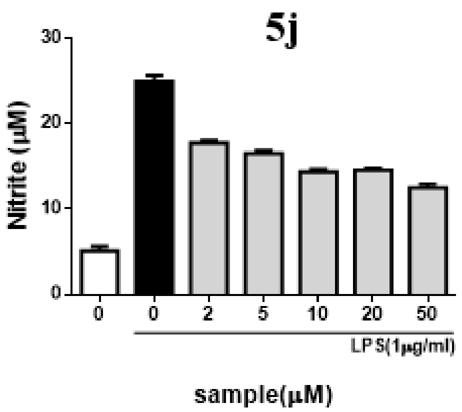
5j
Nitrite (μM)
0
10
20
30
0
0
2
5
10
20
50
LPS(1μg/ml)
sample(μM)

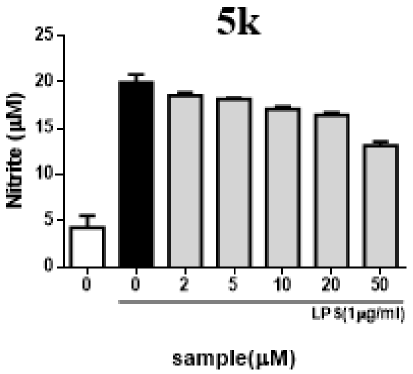
5k
Nitrite (μM)
0
5
10
15
20
25
0
0
2
5
10
20
50
LPS(1μg/ml)
sample(μM)

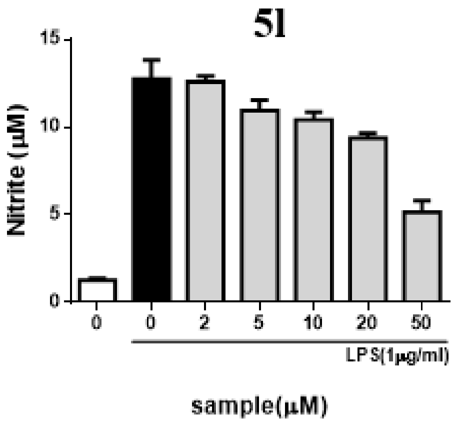
5l
Nitrite (μM)
0
5
10
15
0
0
2
5
10
20
50
LPS(1μg/ml)
sample(μM)

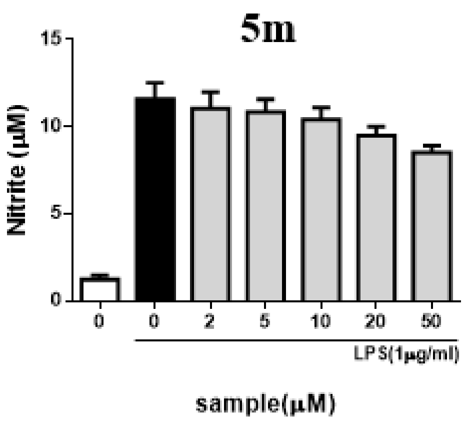
5m
Nitrite (μM)
0
5
10
15
0
0
2
5
10
20
50
LPS(1μg/ml)
sample(μM)

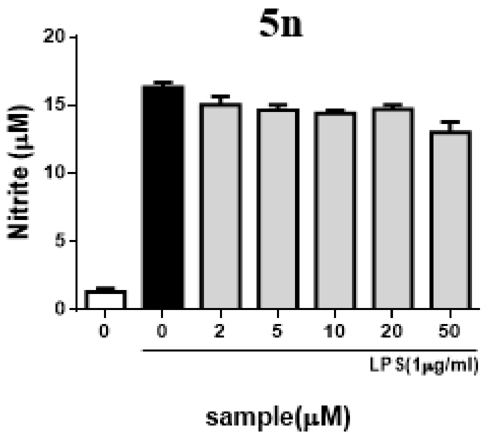

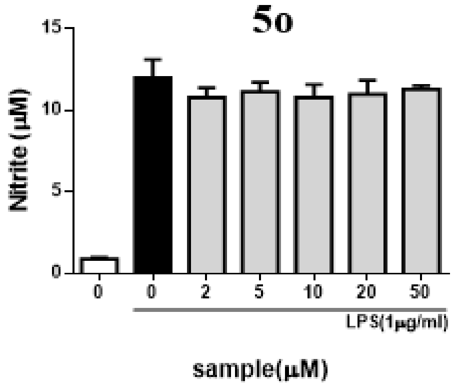

3. SCXRD (3a)

Single Crystal X-ray Diffraction. A colorless block crystal with a size of 0.488 mm x 0.388 mm x 0.300 mm for **3a** was used for single crystal X-ray diffraction analysis. The diffraction data were collected at 100 K with a Bruker D8 QUEST diffractometer (Mo Kα radiation). The data were integrated with the SAINT program [S1] and corrected by the SADABS program [S2]. The initial structure solution was obtained by a direct method using SHELXS-2013 [S3] and full-matrix least-squares refinement was performed using SHELXL-2013 [S4]. All atoms except for hydrogen were refined with anisotropic displacement parameters and converged for $I > 2\sigma(I)$. All calculations were performed with the WinGX-2014 crystallographic software package [S5]. Crystallographic data, atomic parameters, bond lengths, bond angles, and torsion angles of **3a** are listed in Tables S1-S5.

Table S1. Crystal data and structure refinement for **3a**.

Empirical formula	$C_{21}H_{18}N_2O$	
Formula weight	314.37	
Temperature	100(2) K	
Wavelength	0.71073 Å	
Crystal system	Monoclinic	
Space group	$P2_1/c$	
Unit cell dimensions	a = 13.5856(7) Å	α = 90°
	b = 10.5667(5) Å	β = 97.313(2)°
	c = 11.4992(5) Å	γ = 90°
Volume	1637.34(14) Å^3	
Z	4	
Density (calculated)	1.275 g/cm^3	
Absorption coefficient	0.079 mm^{-1}	
F(000)	664	
Crystal size	0.488 x 0.388 x 0.300 mm^3	
Theta range for data collection	2.628 to 28.355°	
Index ranges	$-18 \le h \le 18$, $-14 \le k \le 14$, $-15 \le l \le 15$	
Reflections collected	58173	
Independent reflections	4077 [R(int) = 0.0309]	
Completeness to theta = 25.242°	99.80%	
Refinement method	Full-matrix least-squares on F^2	
Data / restraints / parameters	4077 / 0 / 218	
Goodness-of-fit on F^2	1.025	
Final R indices [I > 2sigma(I)]	R_1 = 0.0379, wR_2 = 0.0960	
R indices (all data)	R_1 = 0.0444, wR_2 = 0.1004	
Extinction coefficient	n/a	
Largest diff. peak and hole	0.295 and -0.183 e.Å^{-3}	

Table S2. Atomic coordinates (×10^4) and equivalent isotropic displacement parameters (Å^2 x 10^3) for **3a**. *U*(eq) is defined as one third of the trace of the orthogonalized U^{ij} tensor.

	x	*y*	*z*	*U*(eq)
C(1)	8269(1)	5905(1)	8607(1)	16(1)
C(2)	9092(1)	6615(1)	8232(1)	17(1)
C(3)	10106(1)	6601(1)	8645(1)	20(1)
C(4)	10712(1)	7404(1)	8105(1)	24(1)
C(5)	10316(1)	8198(1)	7183(1)	25(1)
C(6)	9311(1)	8235(1)	6786(1)	22(1)
C(7)	8704(1)	7429(1)	7341(1)	17(1)
C(8)	7195(1)	7192(1)	5906(1)	20(1)
C(9)	7378(1)	5933(1)	5349(1)	21(1)
C(10)	6646(1)	5003(1)	5246(1)	27(1)
C(11)	6809(1)	3833(1)	4751(1)	34(1)
C(12)	7710(1)	3579(1)	4354(1)	34(1)
C(13)	8441(1)	4500(1)	4438(1)	32(1)
C(14)	8277(1)	5672(1)	4930(1)	26(1)
C(15)	6442(1)	6174(1)	8102(1)	16(1)
C(16)	6096(1)	4972(1)	8332(1)	19(1)
C(17)	5115(1)	4825(1)	8527(1)	20(1)
C(18)	4456(1)	5842(1)	8457(1)	20(1)
C(19)	4826(1)	7037(1)	8246(1)	21(1)
C(20)	5815(1)	7215(1)	8085(1)	19(1)
C(21)	3376(1)	5645(1)	8588(1)	27(1)
N(1)	7440(1)	6327(1)	7885(1)	17(1)
N(2)	7665(1)	7333(1)	7140(1)	18(1)
O(1)	8251(1)	5130(1)	9405(1)	20(1)

Table S3. Bond lengths [Å] for **3a**.

C(1)-O(1)	1.2322(12)	C(10)-C(11)	1.3904(18)
C(1)-N(1)	1.3861(12)	C(11)-C(12)	1.3853(19)
C(1)-C(2)	1.4561(13)	C(12)-C(13)	1.3858(19)
C(2)-C(7)	1.3900(13)	C(13)-C(14)	1.3916(17)
C(2)-C(3)	1.3986(13)	C(15)-C(20)	1.3888(14)
C(3)-C(4)	1.3828(15)	C(15)-C(16)	1.3914(14)
C(4)-C(5)	1.4045(16)	C(15)-N(1)	1.4191(12)
C(5)-C(6)	1.3843(15)	C(16)-C(17)	1.3880(14)
C(6)-C(7)	1.3952(14)	C(17)-C(18)	1.3948(15)
C(7)-N(2)	1.4038(12)	C(18)-C(19)	1.3912(15)
C(8)-N(2)	1.4879(12)	C(18)-C(21)	1.5080(14)
C(8)-C(9)	1.5100(15)	C(19)-C(20)	1.3922(14)
C(9)-C(10)	1.3927(15)	N(1)-N(2)	1.4221(11)
C(9)-C(14)	1.3953(15)		

Table S4. Bond angles [°] for **3a**.

O(1)-C(1)-N(1)	124.66(9)	C(11)-C(12)-C(13)	119.74(12)
O(1)-C(1)-C(2)	130.63(9)	C(12)-C(13)-C(14)	120.18(11)
N(1)-C(1)-C(2)	104.66(8)	C(13)-C(14)-C(9)	120.62(11)
C(7)-C(2)-C(3)	121.43(9)	C(20)-C(15)-C(16)	120.26(9)
C(7)-C(2)-C(1)	107.66(8)	C(20)-C(15)-N(1)	120.33(9)
C(3)-C(2)-C(1)	130.88(9)	C(16)-C(15)-N(1)	119.41(9)
C(4)-C(3)-C(2)	117.27(10)	C(17)-C(16)-C(15)	119.19(9)
C(3)-C(4)-C(5)	120.85(10)	C(16)-C(17)-C(18)	121.77(9)
C(6)-C(5)-C(4)	122.21(10)	C(19)-C(18)-C(17)	117.74(9)
C(5)-C(6)-C(7)	116.57(10)	C(19)-C(18)-C(21)	121.40(10)
C(2)-C(7)-C(6)	121.63(9)	C(17)-C(18)-C(21)	120.85(10)
C(2)-C(7)-N(2)	110.84(8)	C(18)-C(19)-C(20)	121.49(9)
C(6)-C(7)-N(2)	127.50(9)	C(15)-C(20)-C(19)	119.43(9)
N(2)-C(8)-C(9)	114.78(8)	C(1)-N(1)-C(15)	125.43(8)
C(10)-C(9)-C(14)	118.53(10)	C(1)-N(1)-N(2)	112.35(8)
C(10)-C(9)-C(8)	120.21(10)	C(15)-N(1)-N(2)	118.39(8)
C(14)-C(9)-C(8)	121.26(10)	C(7)-N(2)-N(1)	104.13(7)
C(11)-C(10)-C(9)	120.86(11)	C(7)-N(2)-C(8)	117.67(8)
C(12)-C(11)-C(10)	120.07(11)	N(1)-N(2)-C(8)	113.61(8)

Table S5. Torsion angles [°] for **3a**.

O(1)-C(1)-C(2)-C(7)	-175.93(10)	C(15)-C(16)-C(17)-C(18)	2.41(15)
N(1)-C(1)-C(2)-C(7)	1.46(10)	C(16)-C(17)-C(18)-C(19)	-3.49(15)
O(1)-C(1)-C(2)-C(3)	1.92(18)	C(16)-C(17)-C(18)-C(21)	175.68(9)
N(1)-C(1)-C(2)-C(3)	179.30(10)	C(17)-C(18)-C(19)-C(20)	1.37(15)
C(7)-C(2)-C(3)-C(4)	-1.99(15)	C(21)-C(18)-C(19)-C(20)	-177.80(10)
C(1)-C(2)-C(3)-C(4)	-179.58(10)	C(16)-C(15)-C(20)-C(19)	-2.91(14)
C(2)-C(3)-C(4)-C(5)	0.25(16)	N(1)-C(15)-C(20)-C(19)	177.00(9)
C(3)-C(4)-C(5)-C(6)	1.06(17)	C(18)-C(19)-C(20)-C(15)	1.78(15)
C(4)-C(5)-C(6)-C(7)	-0.61(16)	O(1)-C(1)-N(1)-C(15)	15.27(15)
C(3)-C(2)-C(7)-C(6)	2.51(15)	C(2)-C(1)-N(1)-C(15)	-162.32(9)
C(1)-C(2)-C(7)-C(6)	-179.40(9)	O(1)-C(1)-N(1)-N(2)	172.72(9)
C(3)-C(2)-C(7)-N(2)	-175.65(9)	C(2)-C(1)-N(1)-N(2)	-4.87(10)
C(1)-C(2)-C(7)-N(2)	2.43(11)	C(20)-C(15)-N(1)-C(1)	126.74(10)
C(5)-C(6)-C(7)-C(2)	-1.15(15)	C(16)-C(15)-N(1)-C(1)	-53.35(13)
C(5)-C(6)-C(7)-N(2)	176.69(10)	C(20)-C(15)-N(1)-N(2)	-29.48(13)
N(2)-C(8)-C(9)-C(10)	101.43(11)	C(16)-C(15)-N(1)-N(2)	150.43(9)
N(2)-C(8)-C(9)-C(14)	-78.36(12)	C(2)-C(7)-N(2)-N(1)	-5.18(10)
C(14)-C(9)-C(10)-C(11)	0.74(16)	C(6)-C(7)-N(2)-N(1)	176.79(10)
C(8)-C(9)-C(10)-C(11)	-179.06(10)	C(2)-C(7)-N(2)-C(8)	-131.93(9)
C(9)-C(10)-C(11)-C(12)	0.18(18)	C(6)-C(7)-N(2)-C(8)	50.04(14)
C(10)-C(11)-C(12)-C(13)	-0.89(18)	C(1)-N(1)-N(2)-C(7)	6.29(10)
C(11)-C(12)-C(13)-C(14)	0.67(18)	C(15)-N(1)-N(2)-C(7)	165.48(8)
C(12)-C(13)-C(14)-C(9)	0.26(17)	C(1)-N(1)-N(2)-C(8)	135.53(8)
C(10)-C(9)-C(14)-C(13)	-0.96(16)	C(15)-N(1)-N(2)-C(8)	-65.28(11)
C(8)-C(9)-C(14)-C(13)	178.84(10)	C(9)-C(8)-N(2)-C(7)	69.83(11)
C(20)-C(15)-C(16)-C(17)	0.86(14)	C(9)-C(8)-N(2)-N(1)	-52.18(11)
N(1)-C(15)-C(16)-C(17)	-179.05(9)		

4. References

S1. SAINT: Area-Detector Integration Software, Siemens Industrial Automation, Inc.; Madison: **1996**.

S2. SADABS: Area-Detector Absorption Correction, Siemens Industrial Automation, Inc.: Madison: **1995**.

S3. Sheldrick, G. M. SHELXS-2013 - A Program for Automatic Solution of Crystal Structures; University of Goettingen: Goettingen, Germany, **2013**.

S4. Sheldrick, G. M. SHELXL-2013 - A Program for Crystal Structure Refinement; University of Goettingen: Goettingen, Germany, **2013**.

S5. Farrugia, L. J. *J. Appl. Cryst.* **2012**, *45*, 849-854.

5. ^{1}H and ^{13}C NMR Spectra of Compounds

Figure S3. ^{1}H and ^{13}C NMR Spectra of Compounds **3a**.

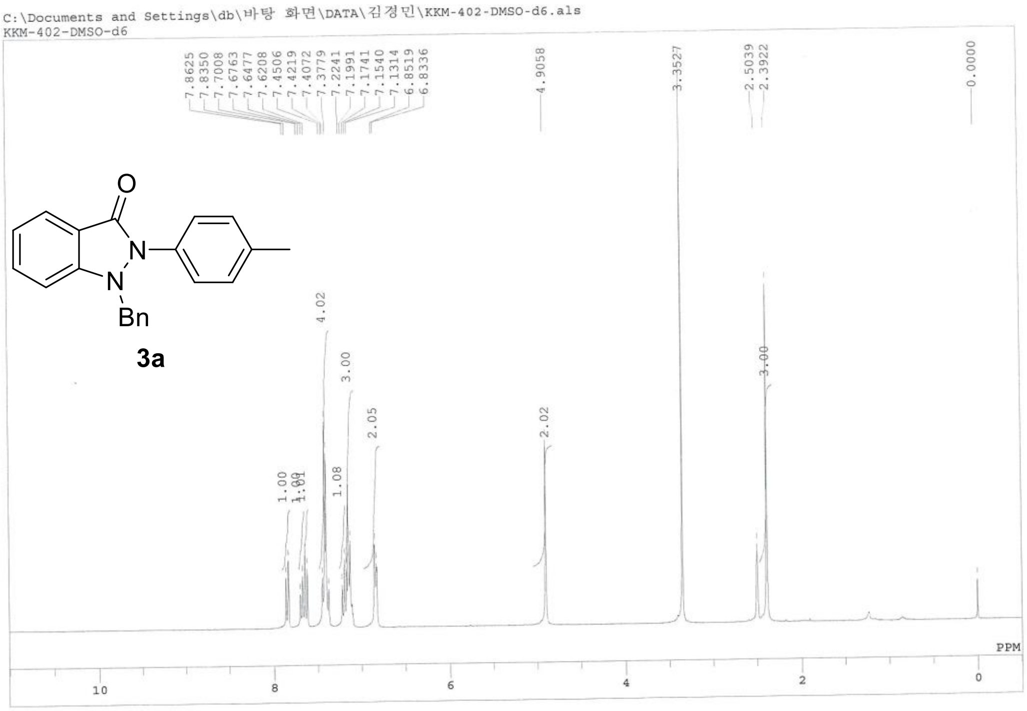

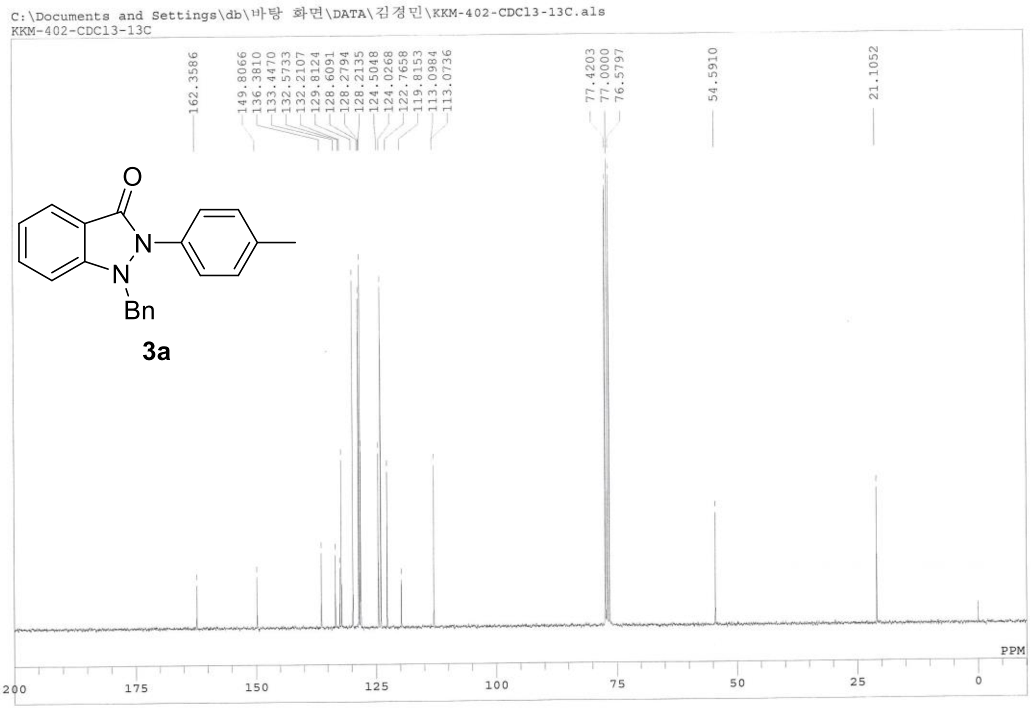

Figure S4. ^{1}H and ^{13}C NMR Spectra of Compounds **3b**.

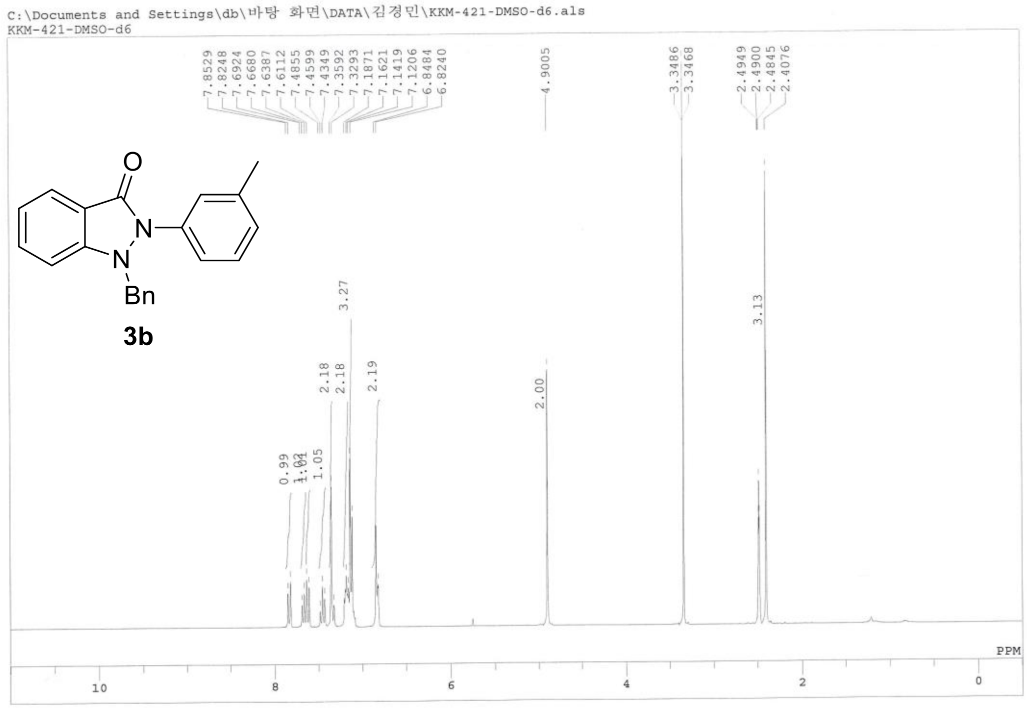

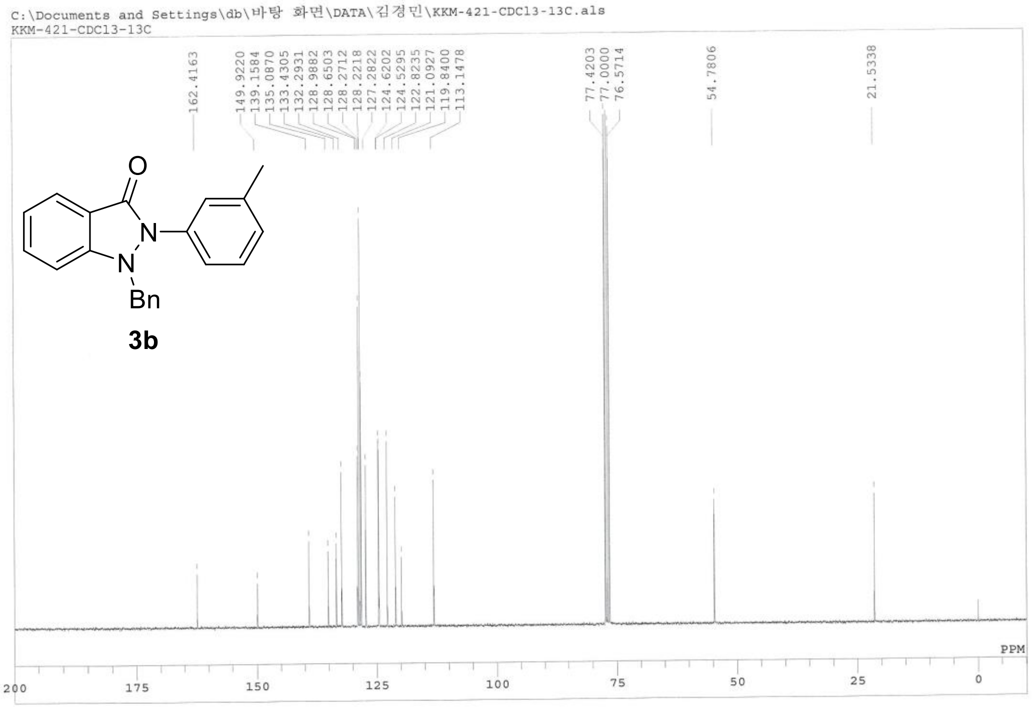

Figure S5. ^{1}H and ^{13}C NMR Spectra of Compounds **3c**.

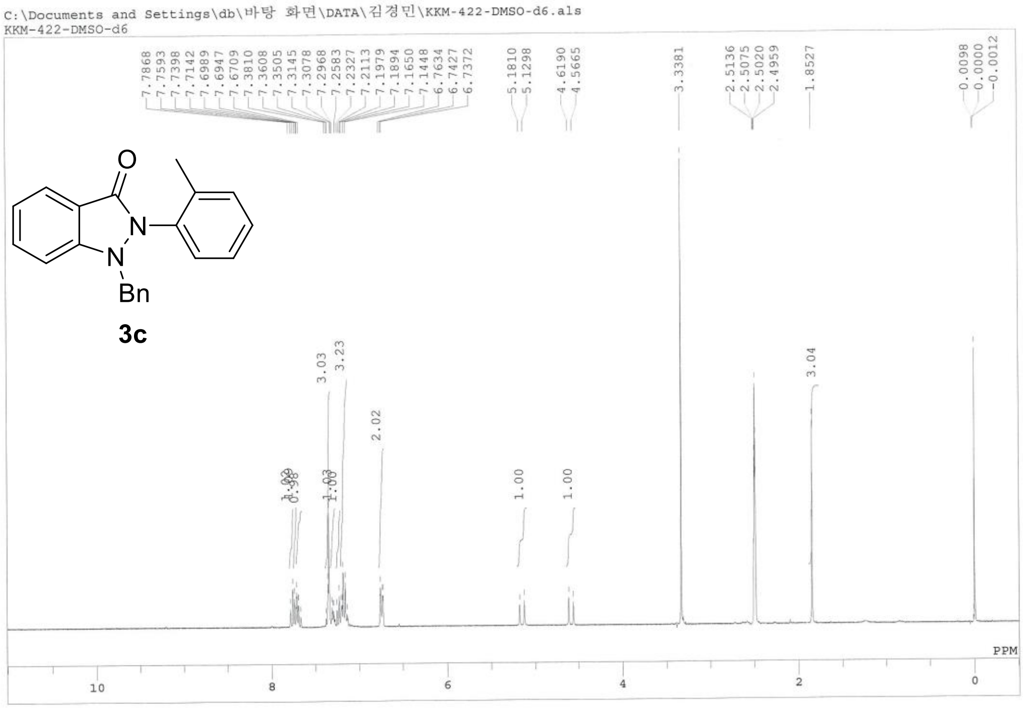

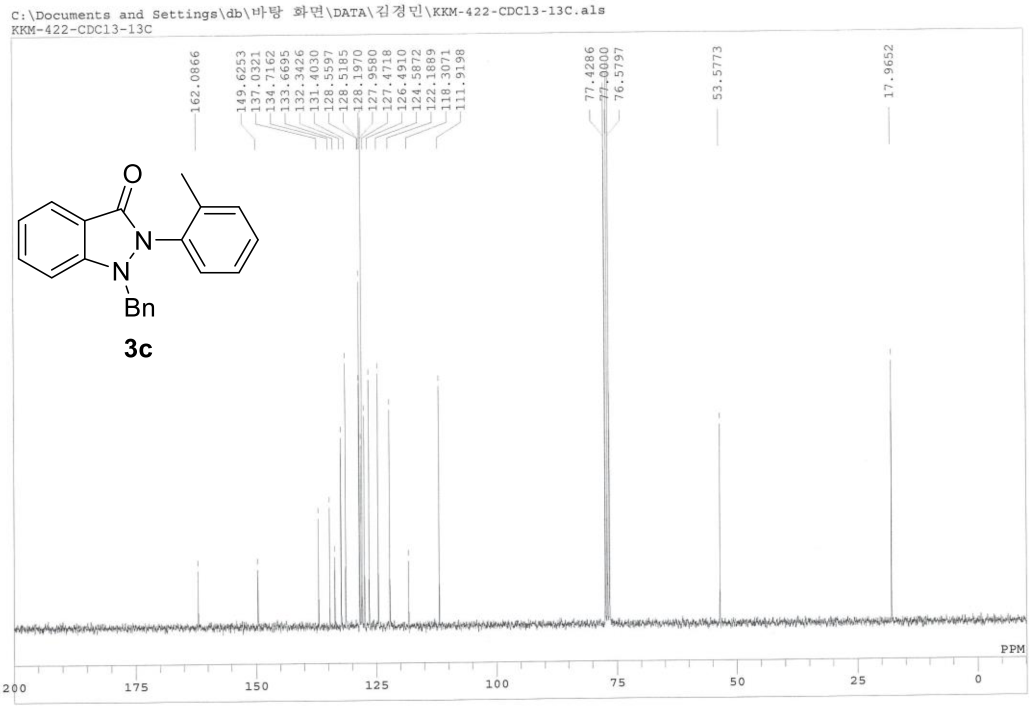

Figure S6. ^{1}H and ^{13}C NMR Spectra of Compounds **3d**.

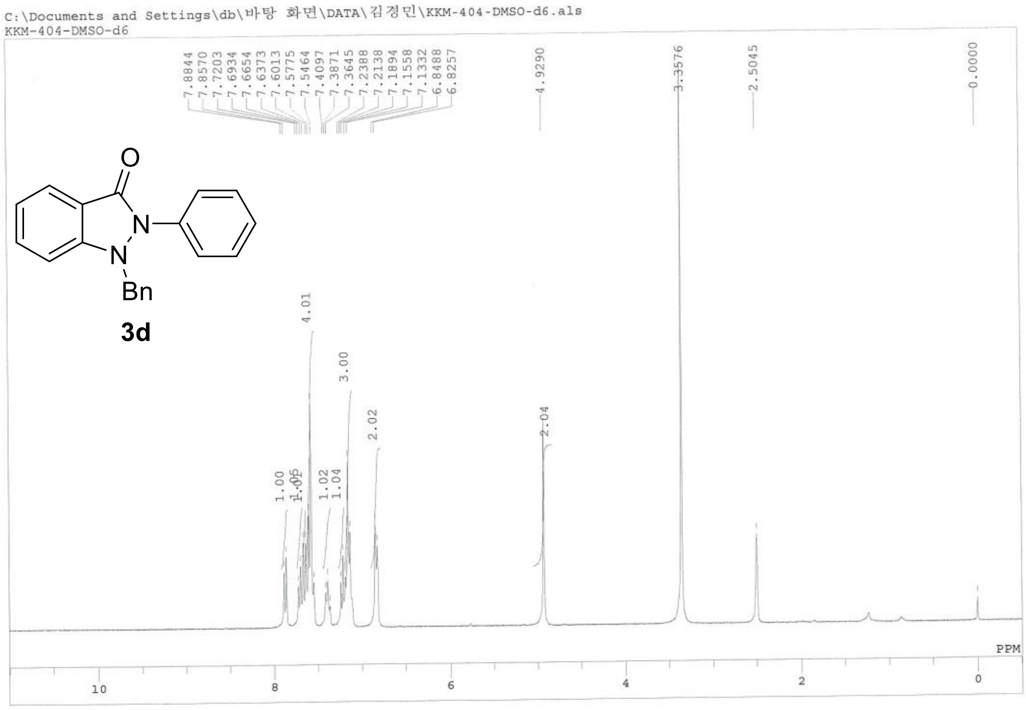

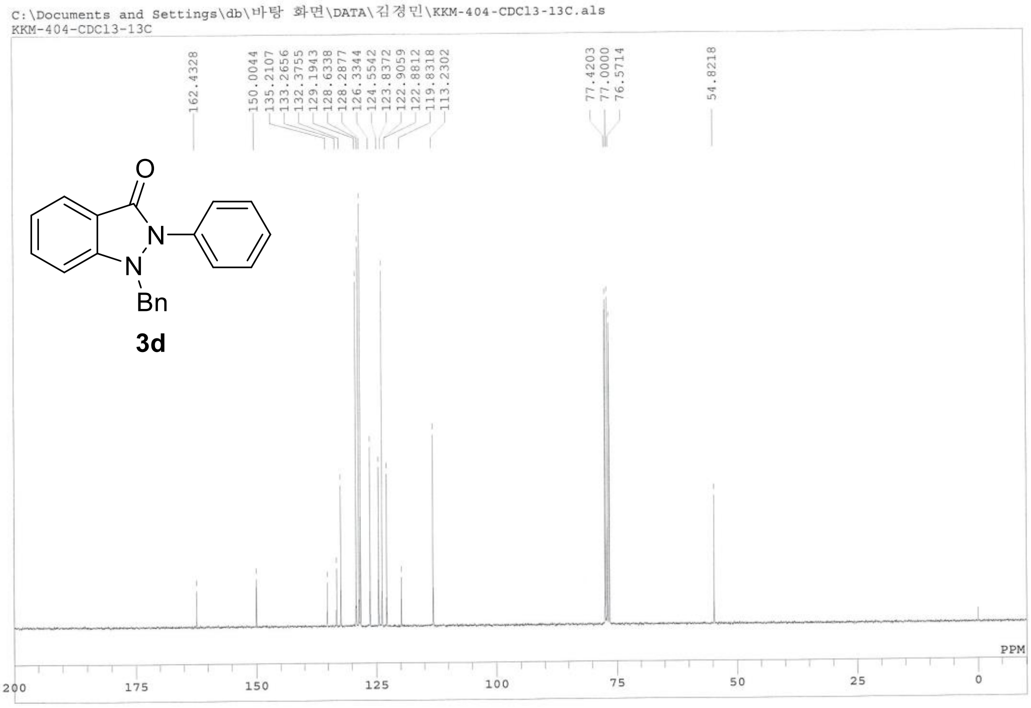

Figure S7. ^{1}H and ^{13}C NMR Spectra of Compounds **3e**.

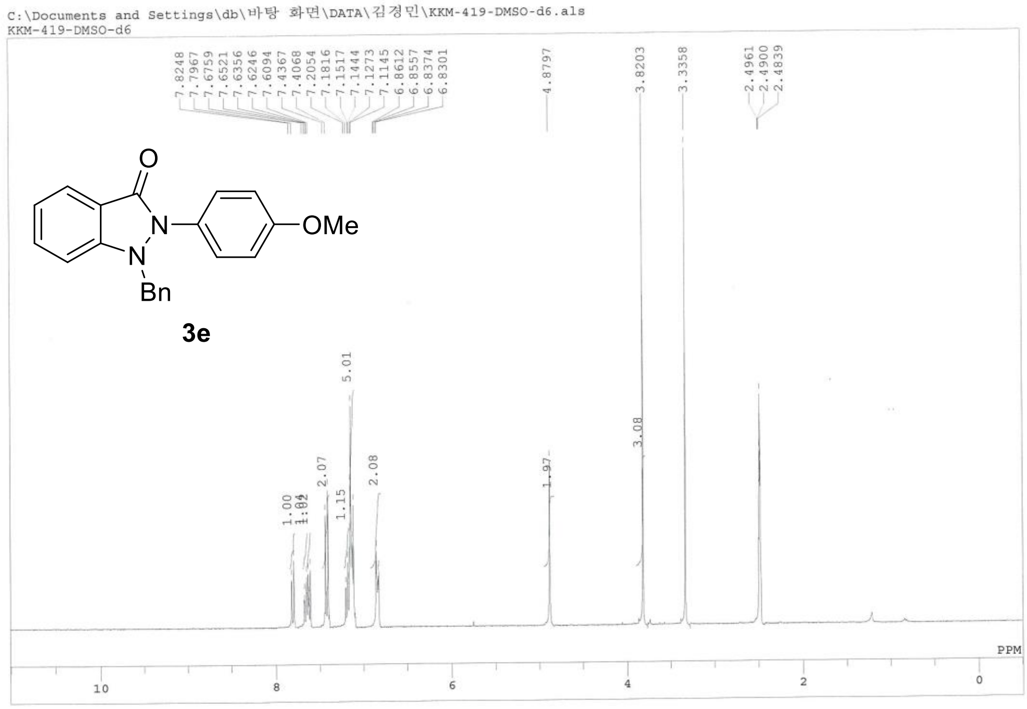

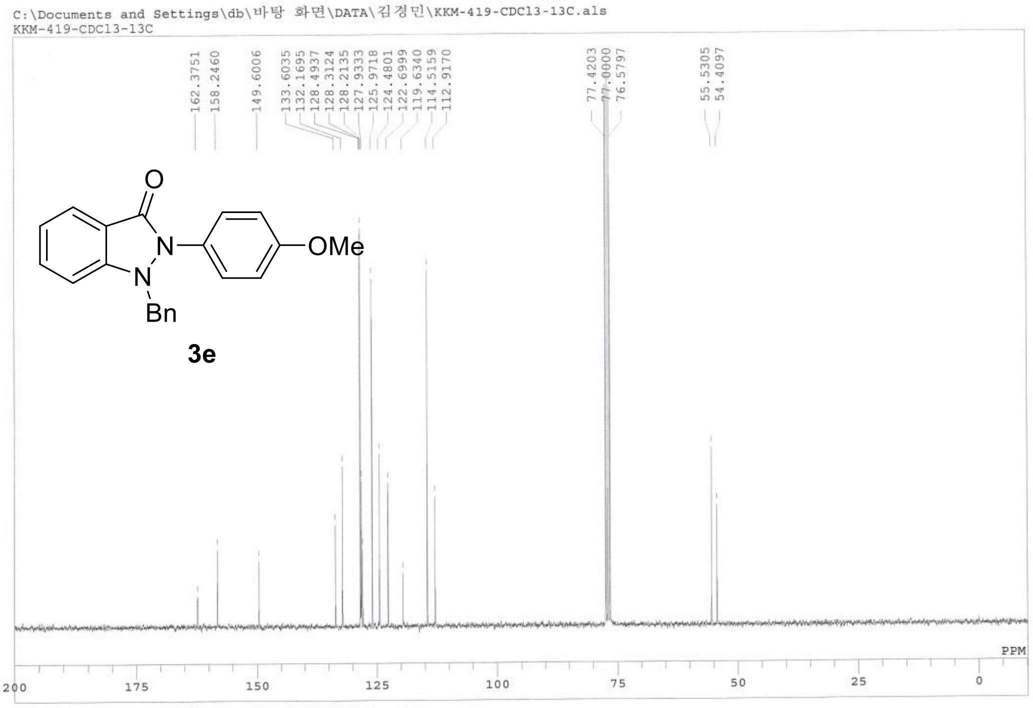

Figure S8. ^{1}H and ^{13}C NMR Spectra of Compounds **3f**.

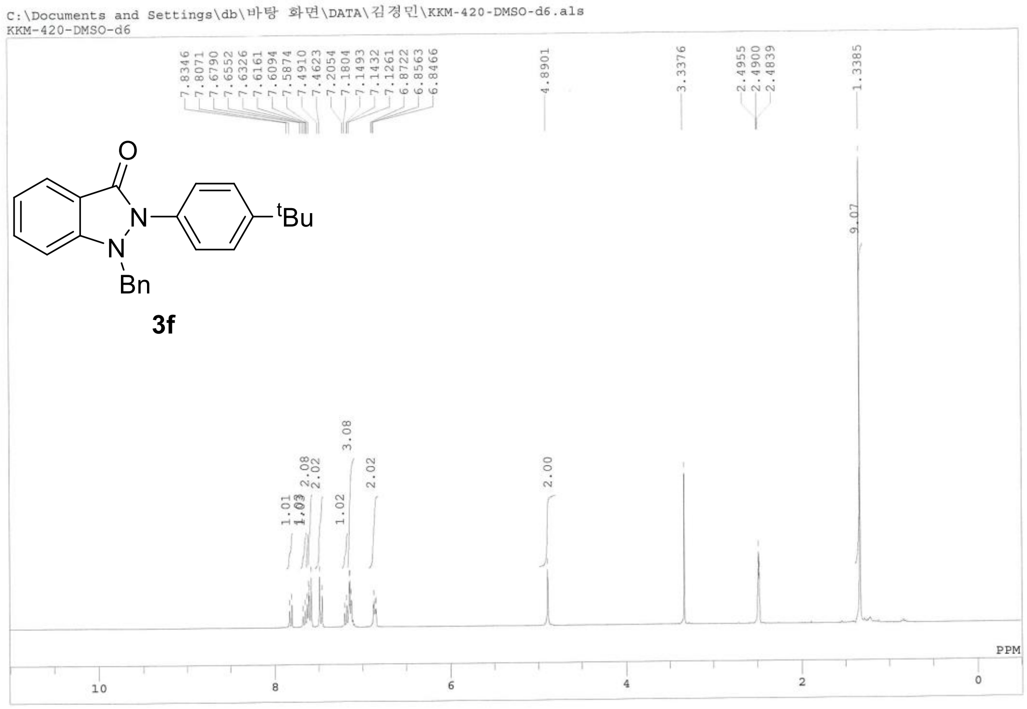

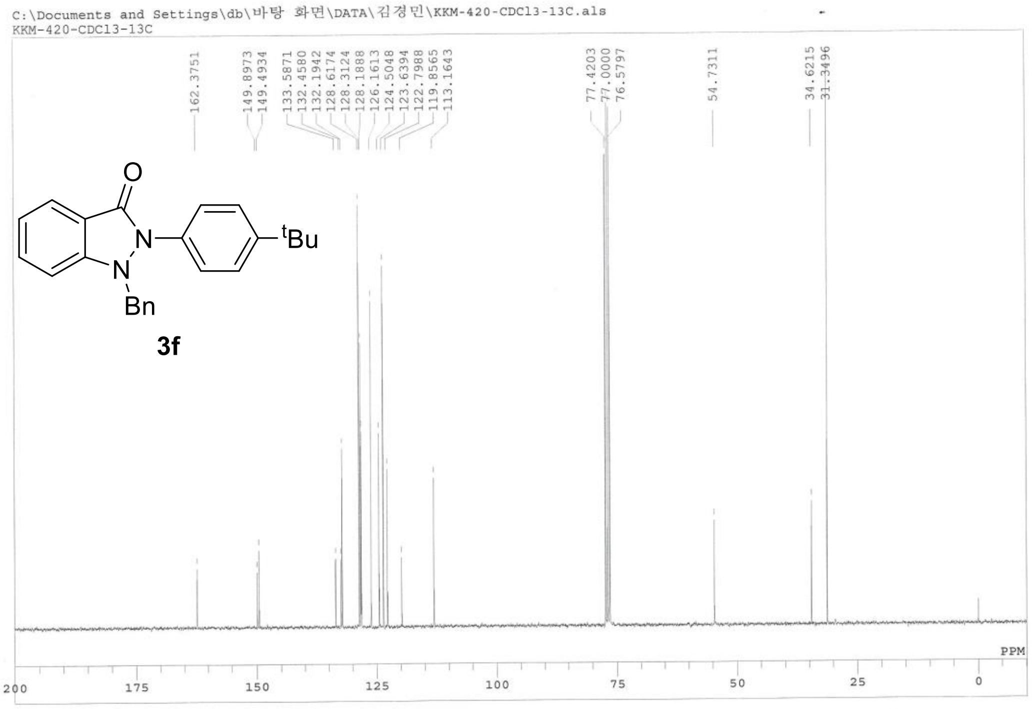

Figure S9. ^{1}H and ^{13}C NMR Spectra of Compounds **3g**.

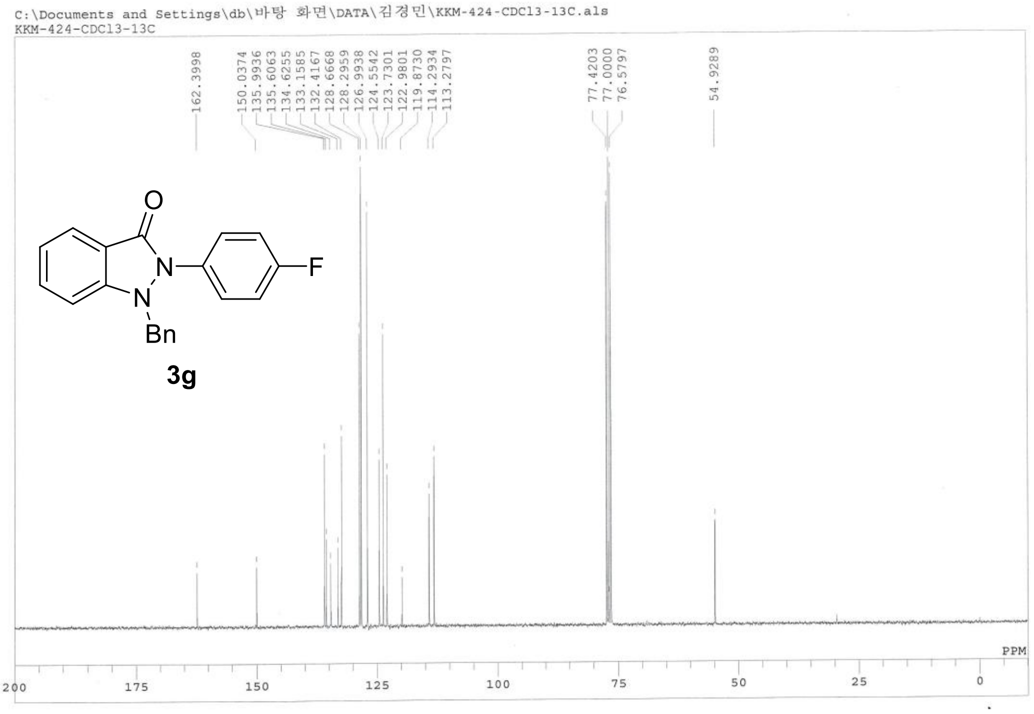

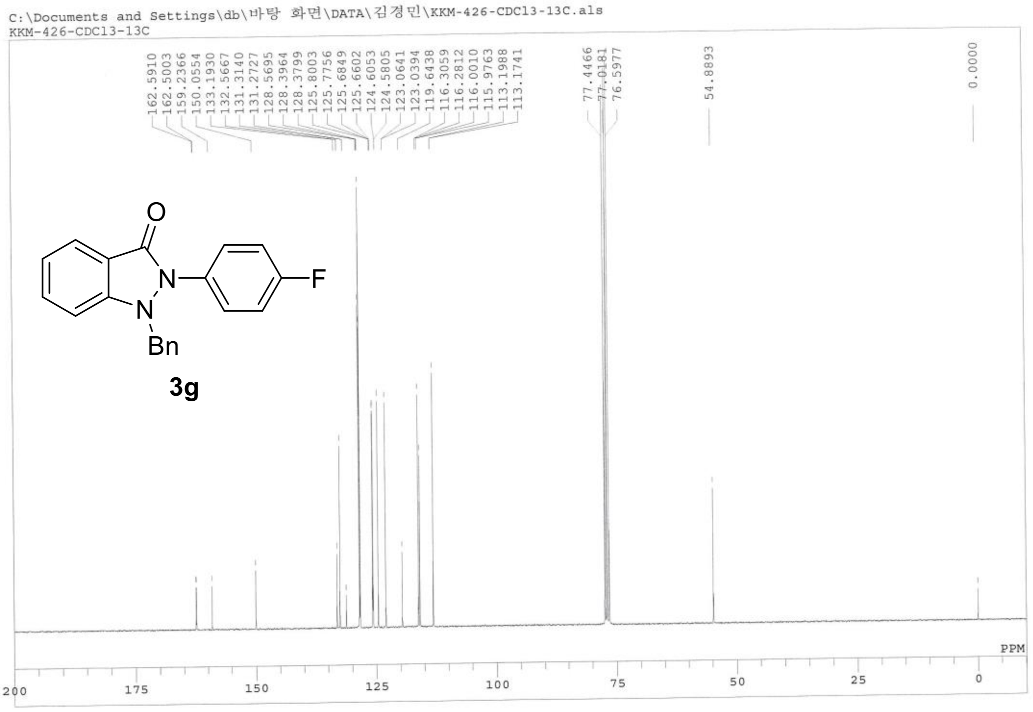

Figure S10. ^{1}H and ^{13}C NMR Spectra of Compounds **3h**.

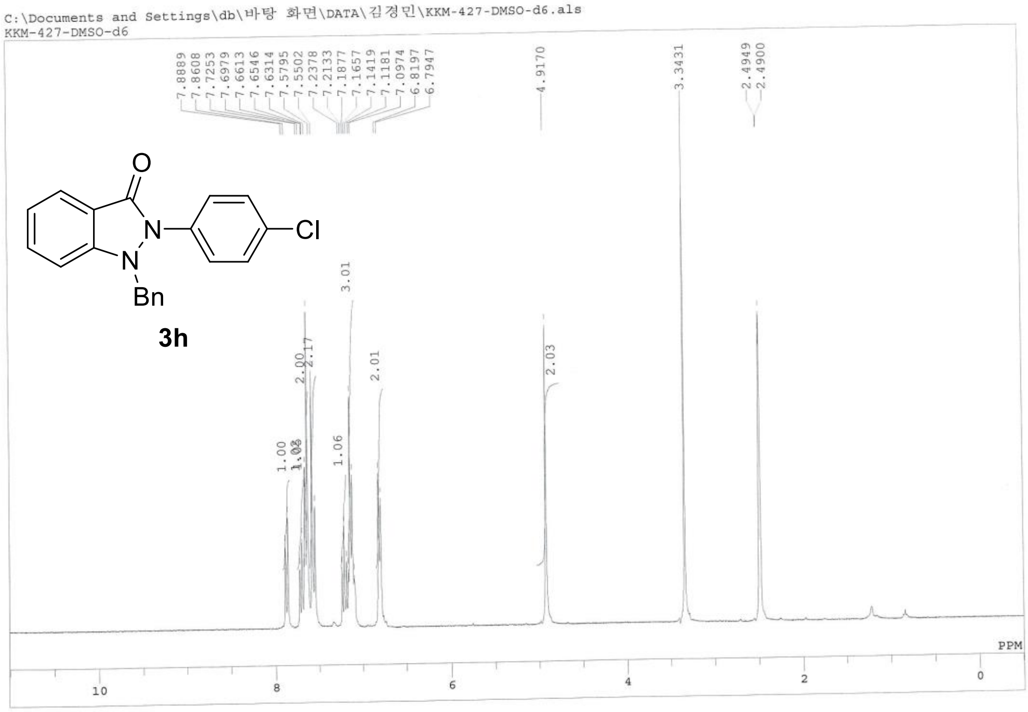

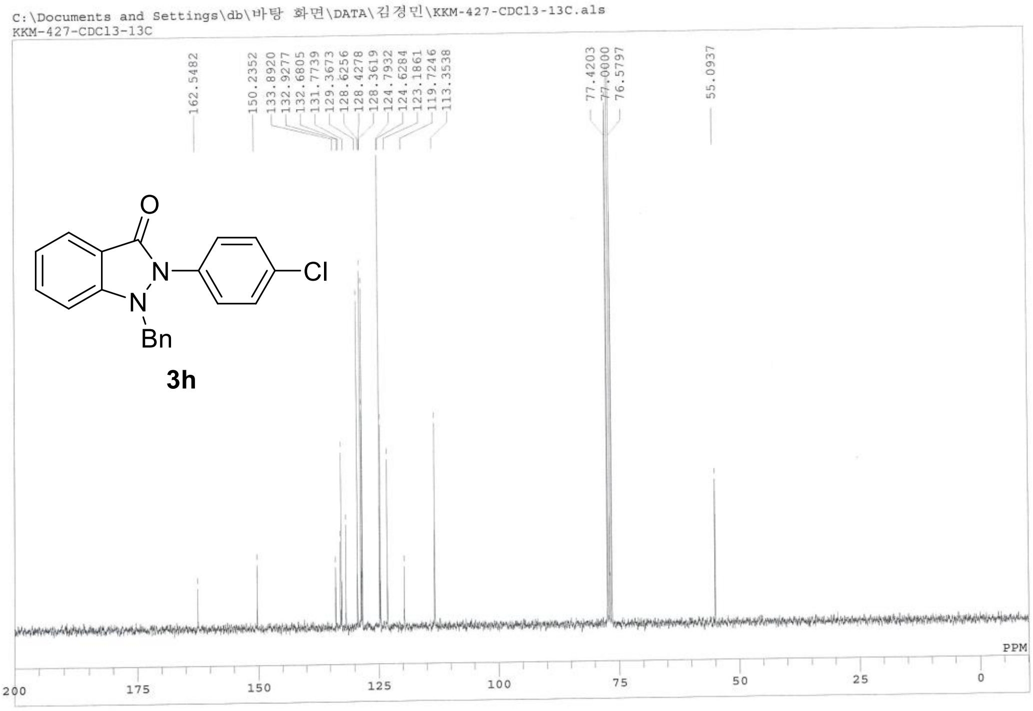

Figure S11. ^{1}H and ^{13}C NMR Spectra of Compounds **3i**.

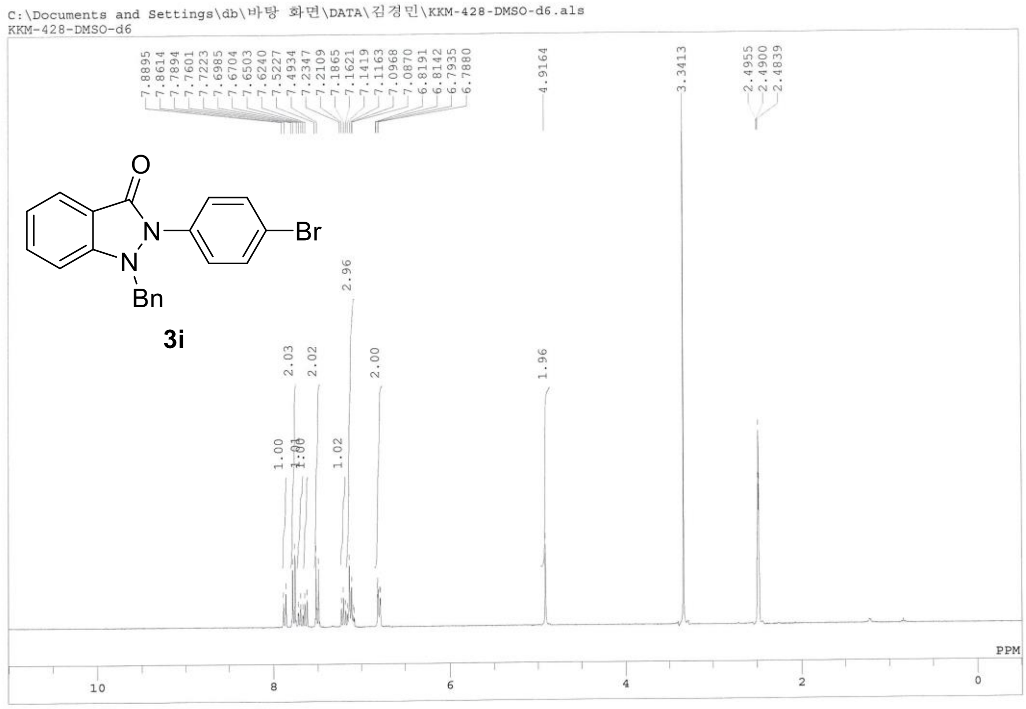

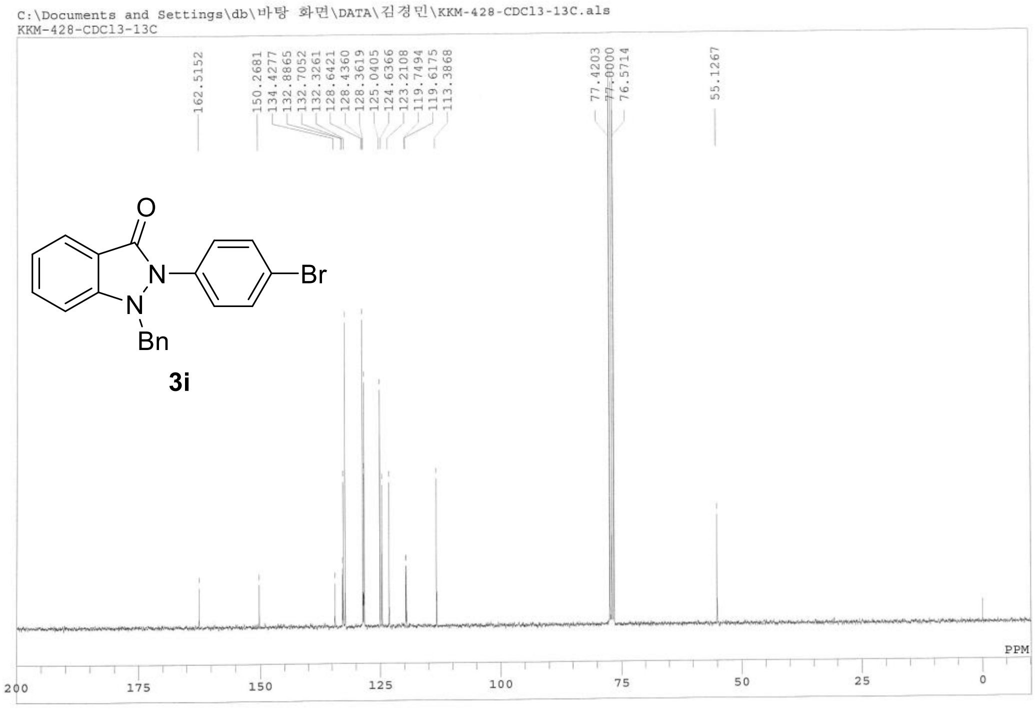

Figure S12. ^{1}H and ^{13}C NMR Spectra of Compounds **3j**.

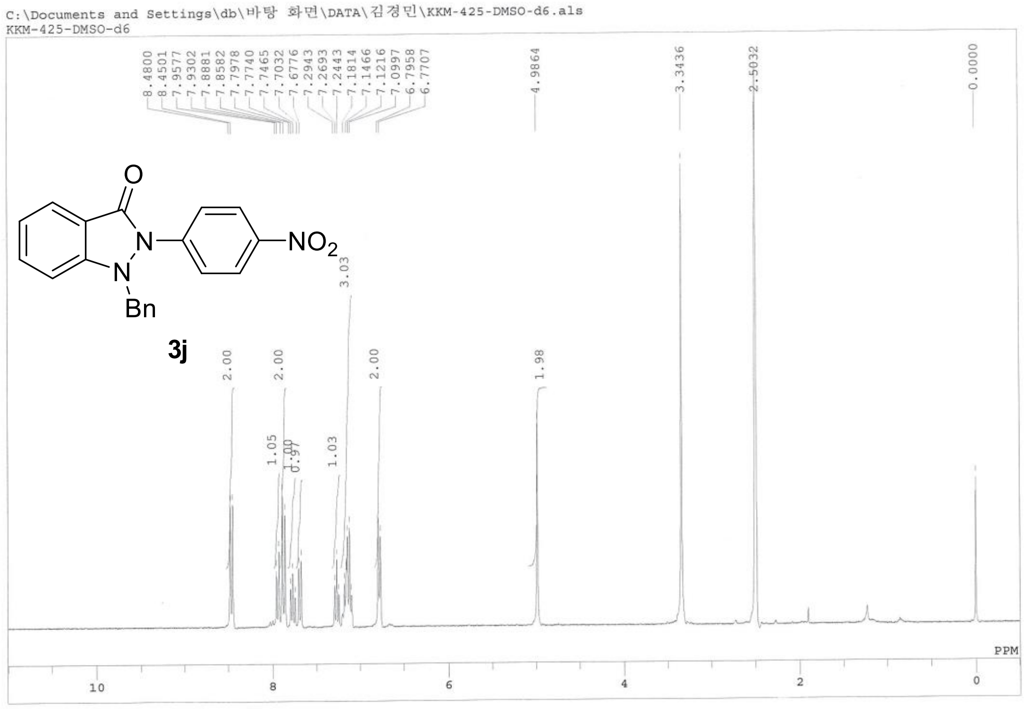

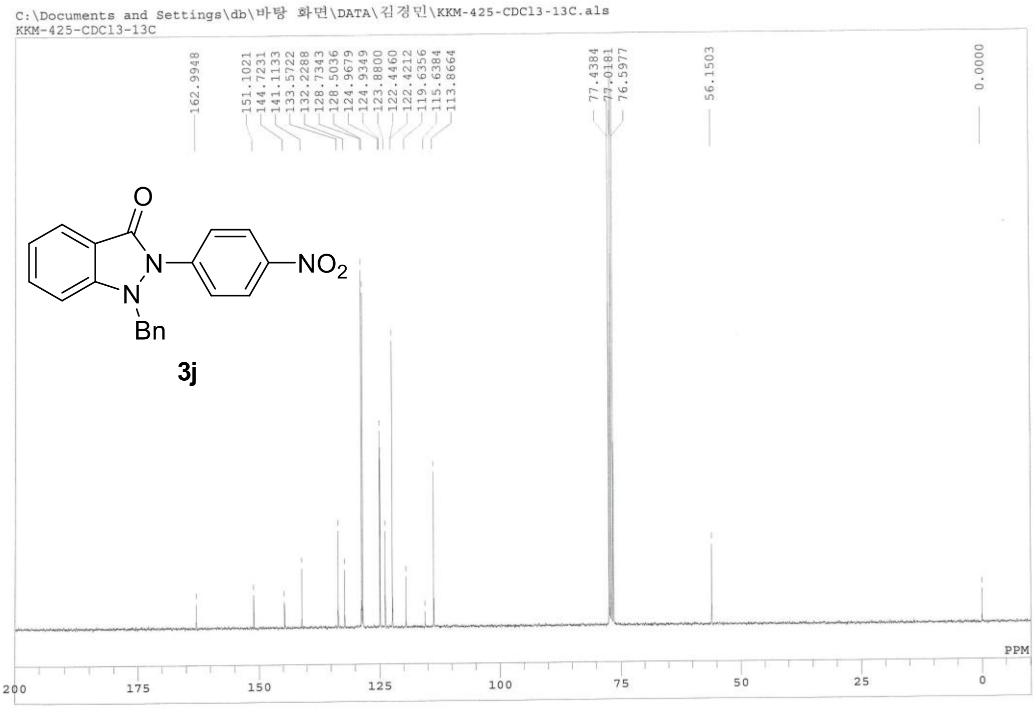

Figure S13. ^{1}H and ^{13}C NMR Spectra of Compounds **4j**.

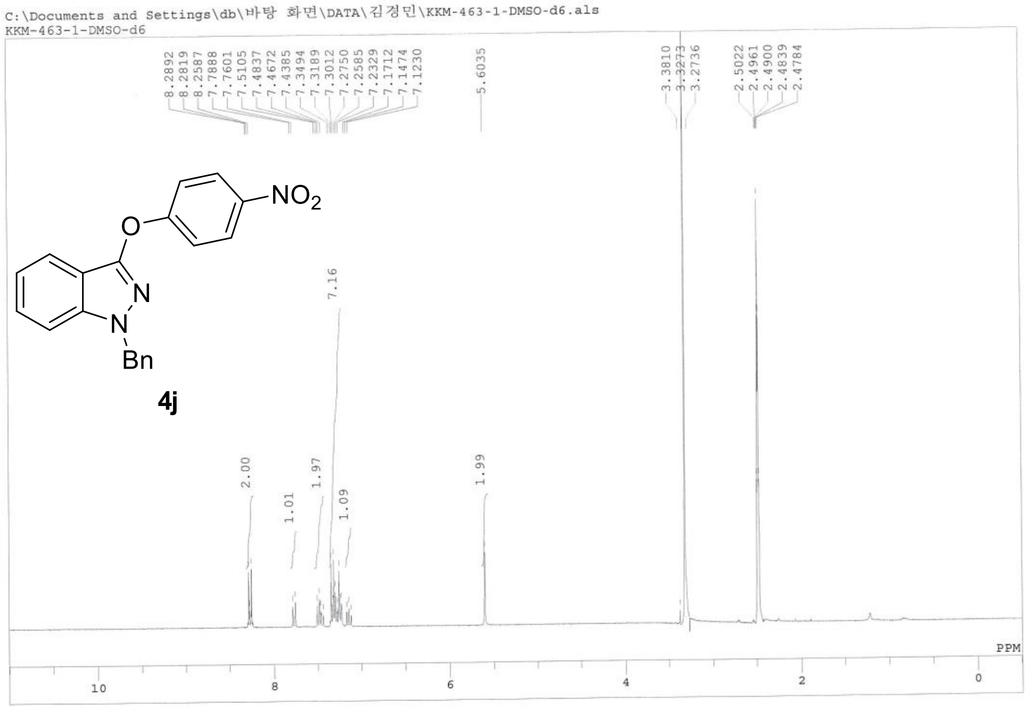

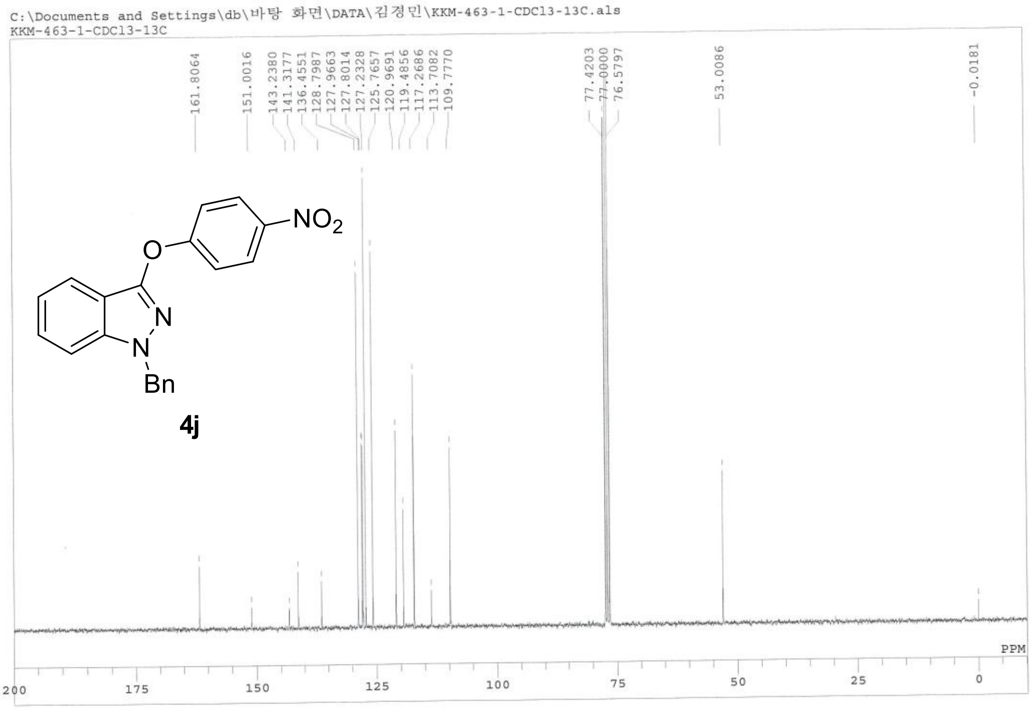

Figure S14. ^{1}H and ^{13}C NMR Spectra of Compounds **3k**.

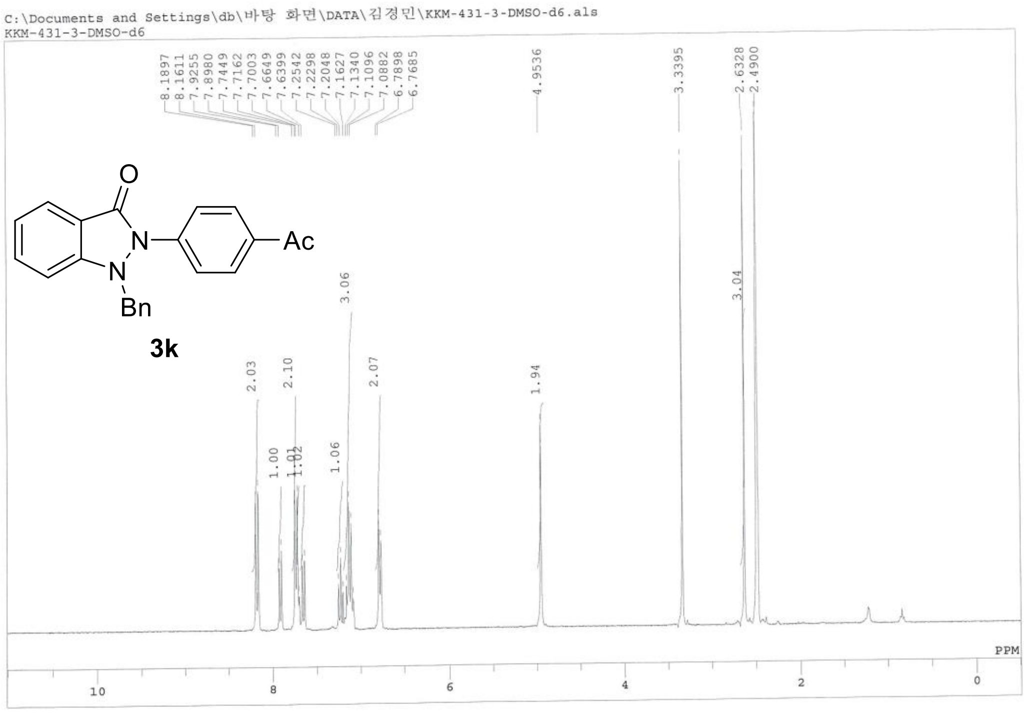

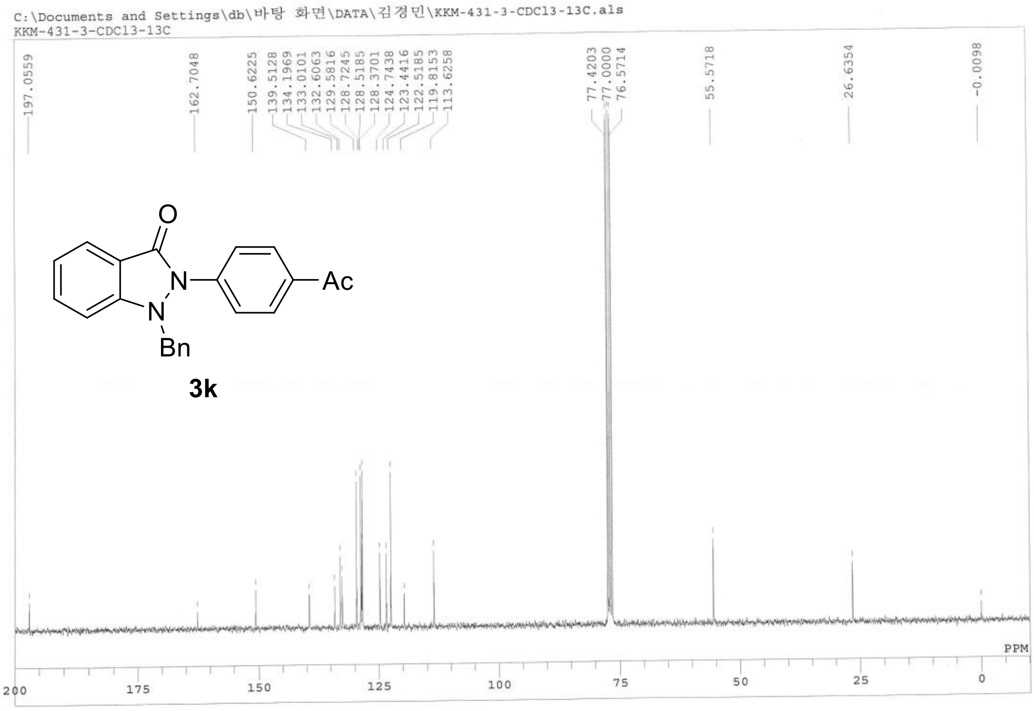

Figure S15. ^{1}H and ^{13}C NMR Spectra of Compounds **4k**.

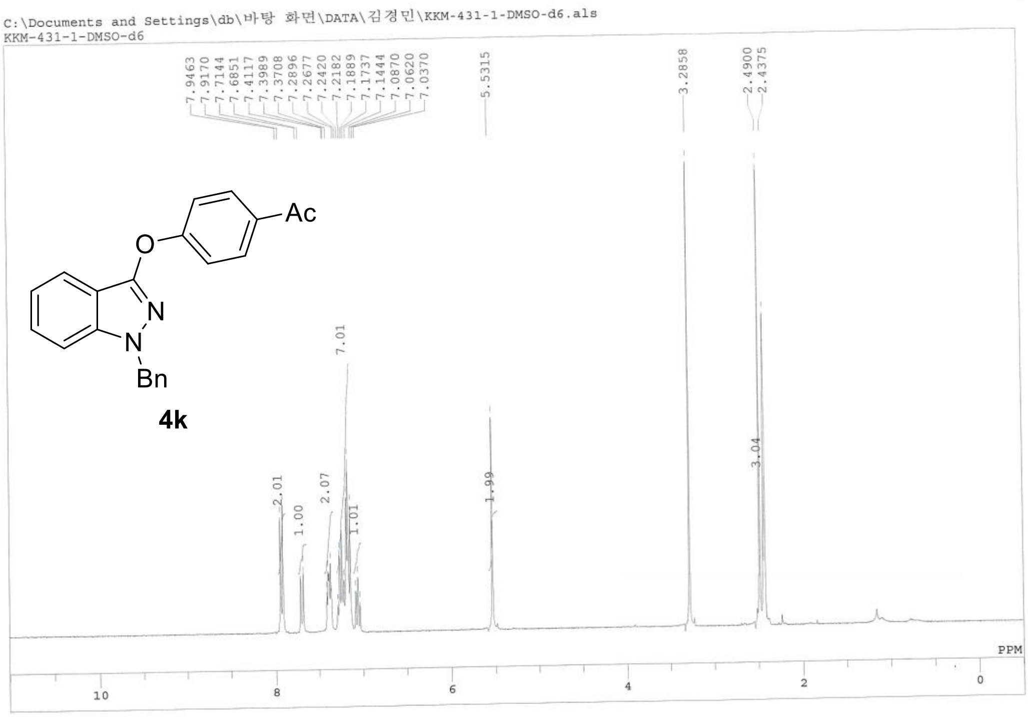

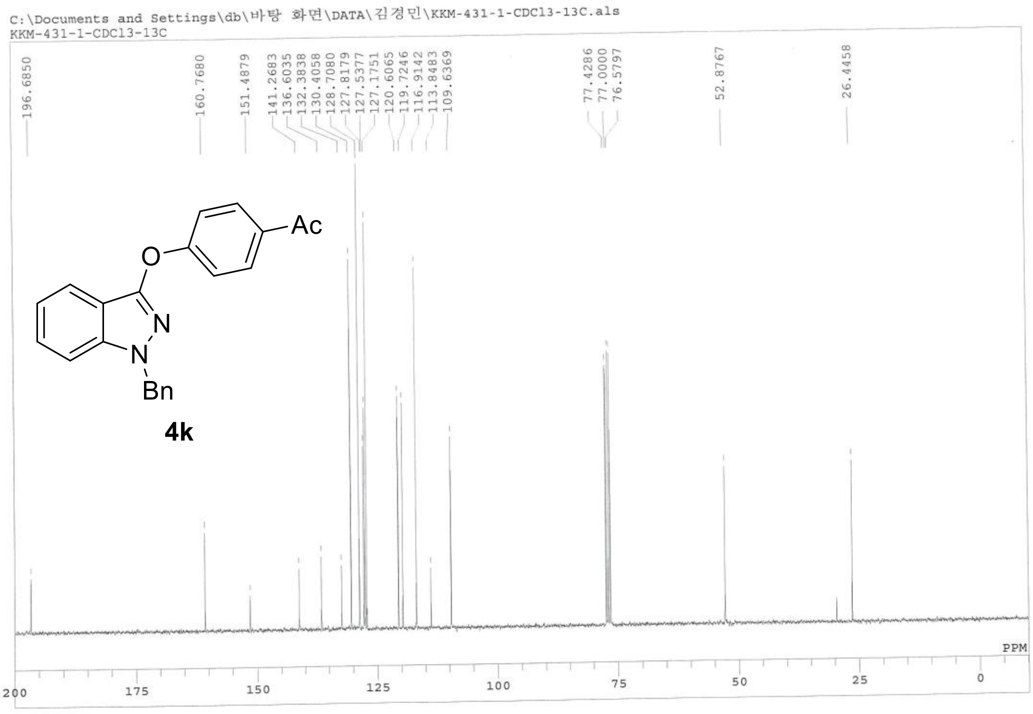

Figure S16. ^{1}H and ^{13}C NMR Spectra of Compounds **3l**.

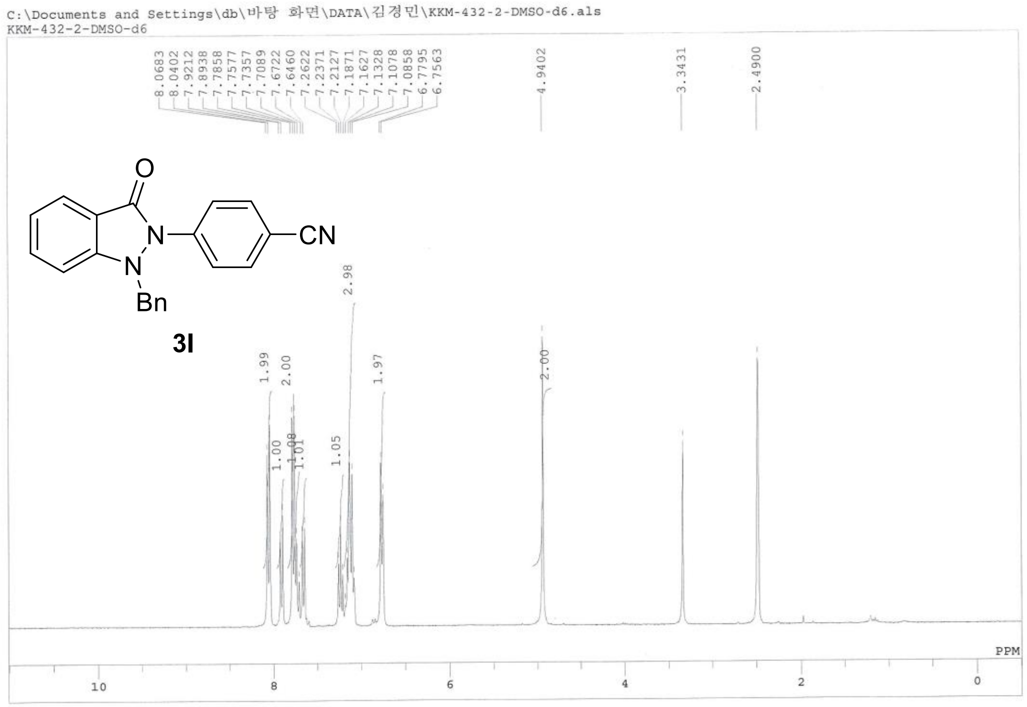

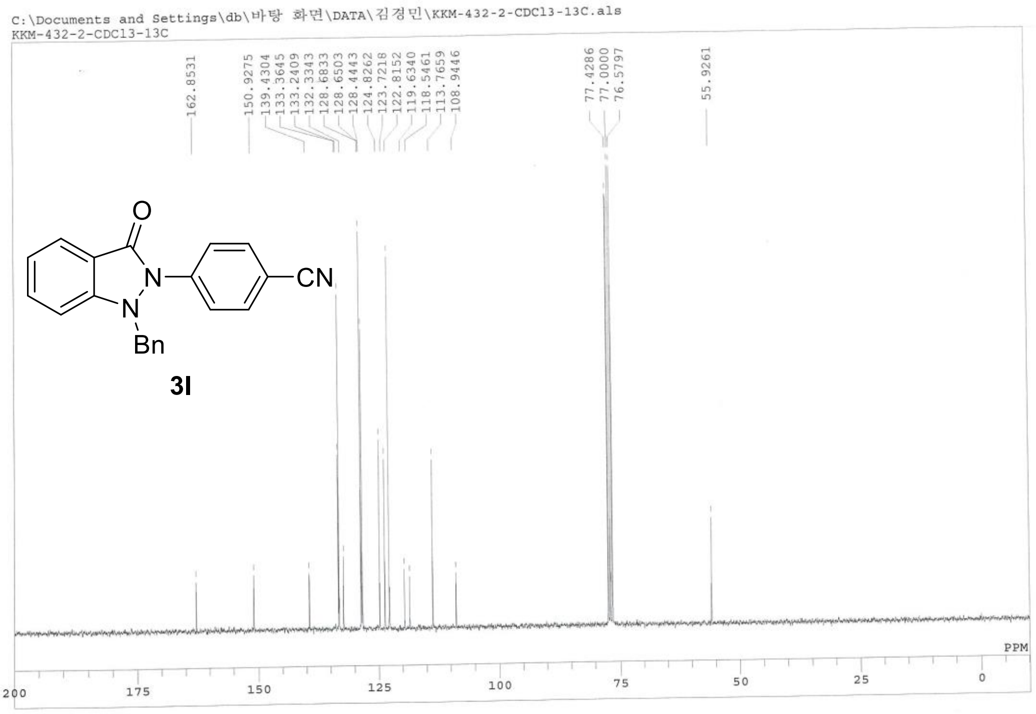

Figure S17. ^{1}H and ^{13}C NMR Spectra of Compounds **4l**.

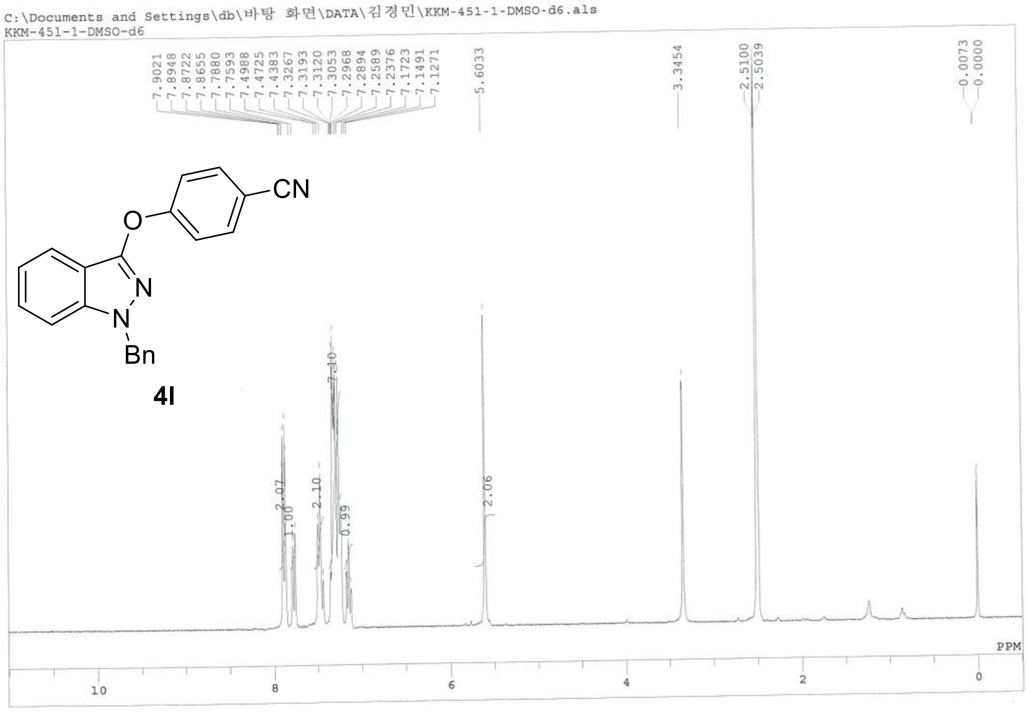

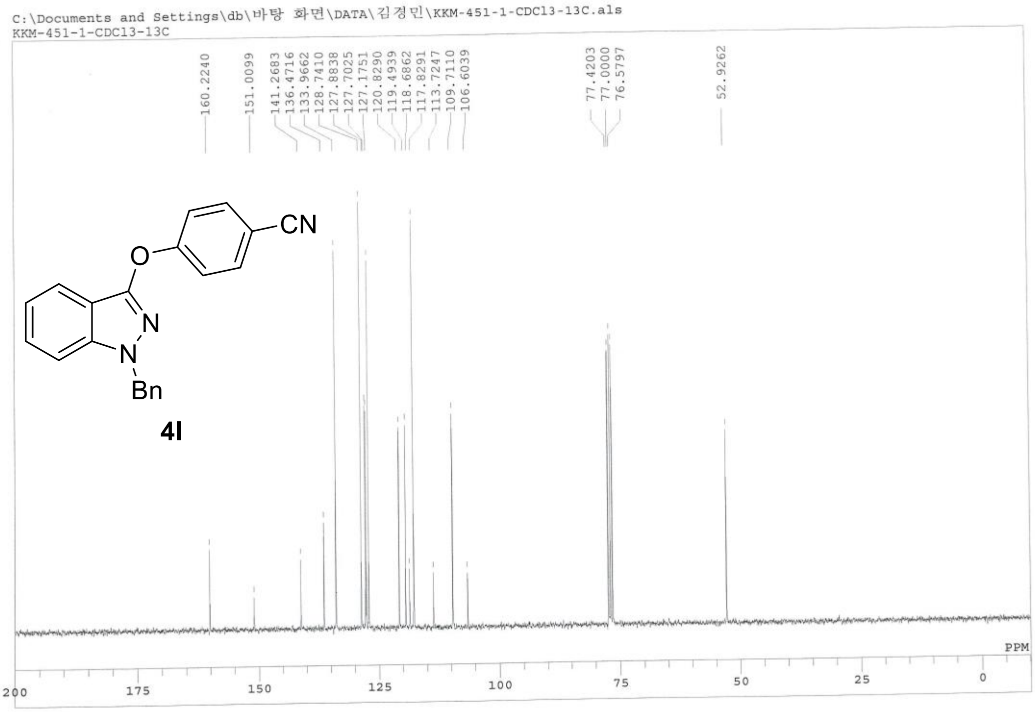

Figure S18. ^{1}H and ^{13}C NMR Spectra of Compounds **3m**.

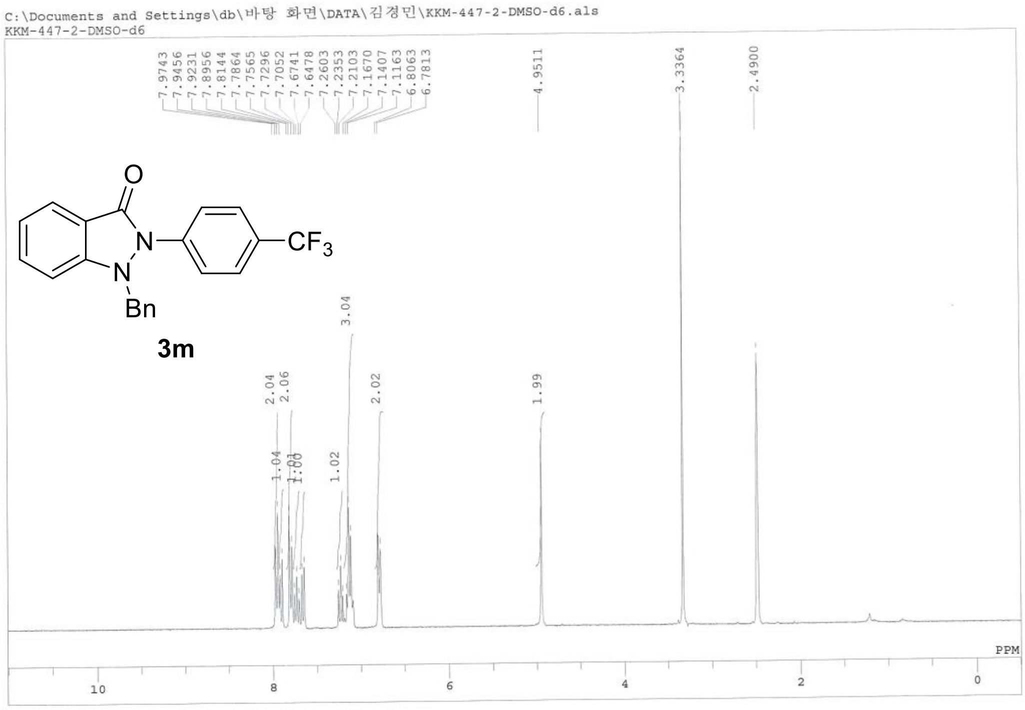

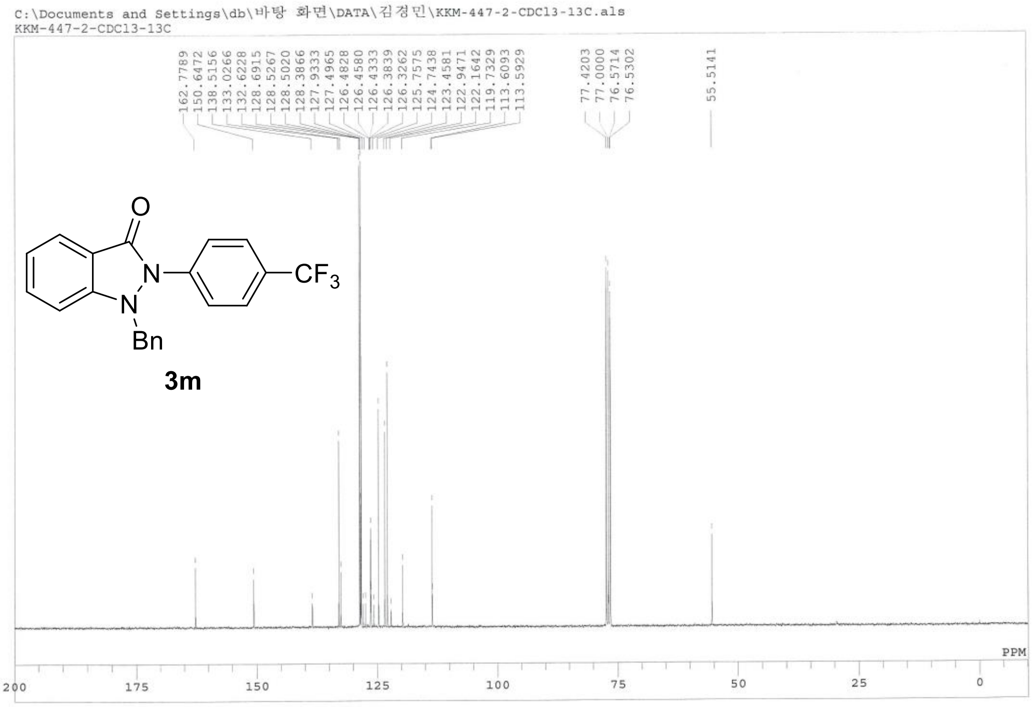

Figure S19. ^{1}H and ^{13}C NMR Spectra of Compounds **3m**.

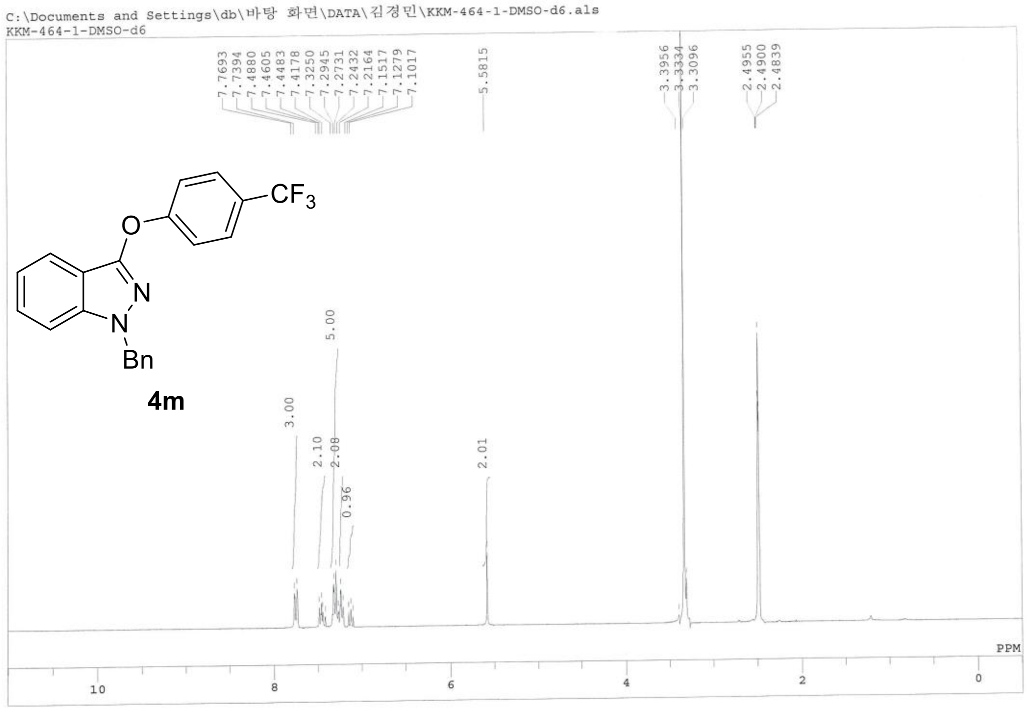

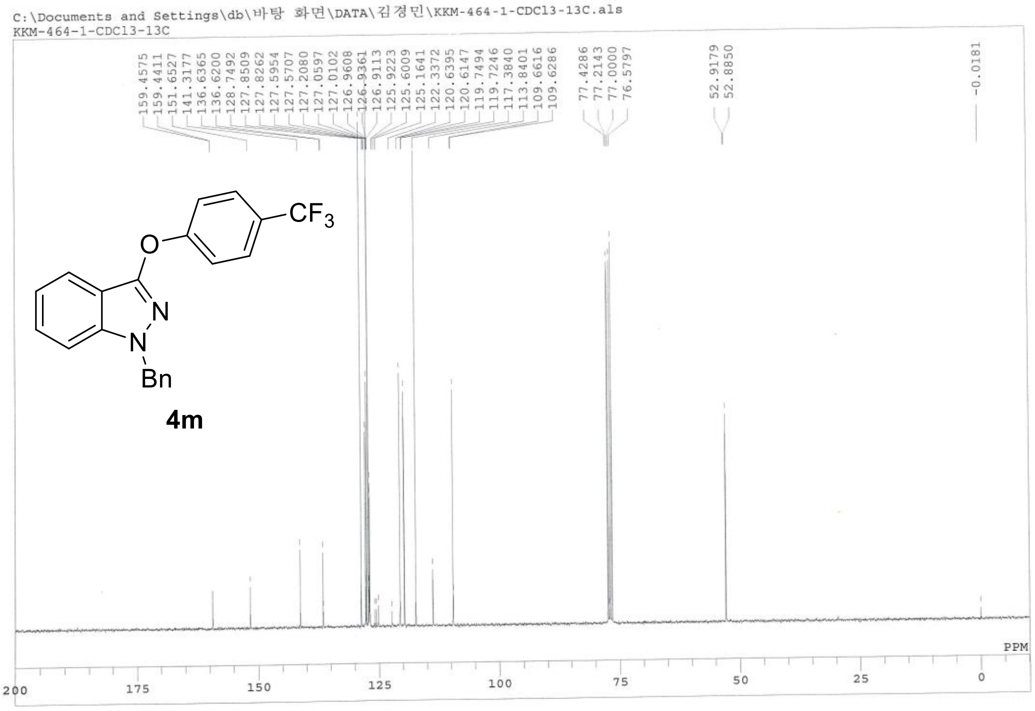

Figure S20. ^{1}H and ^{13}C NMR Spectra of Compounds **3n**.

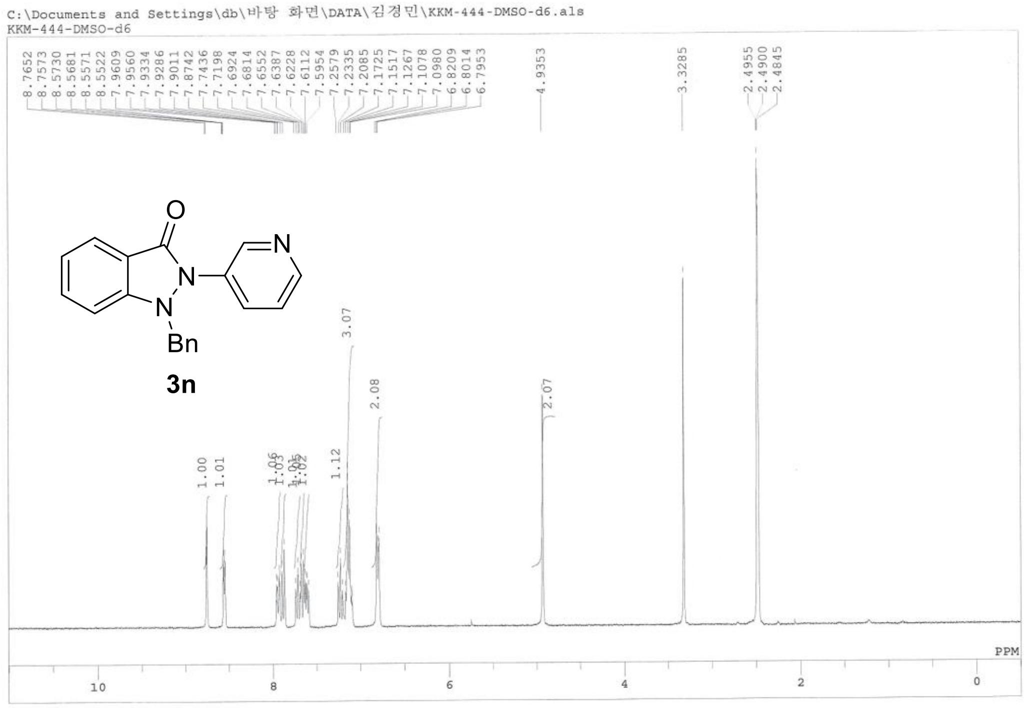

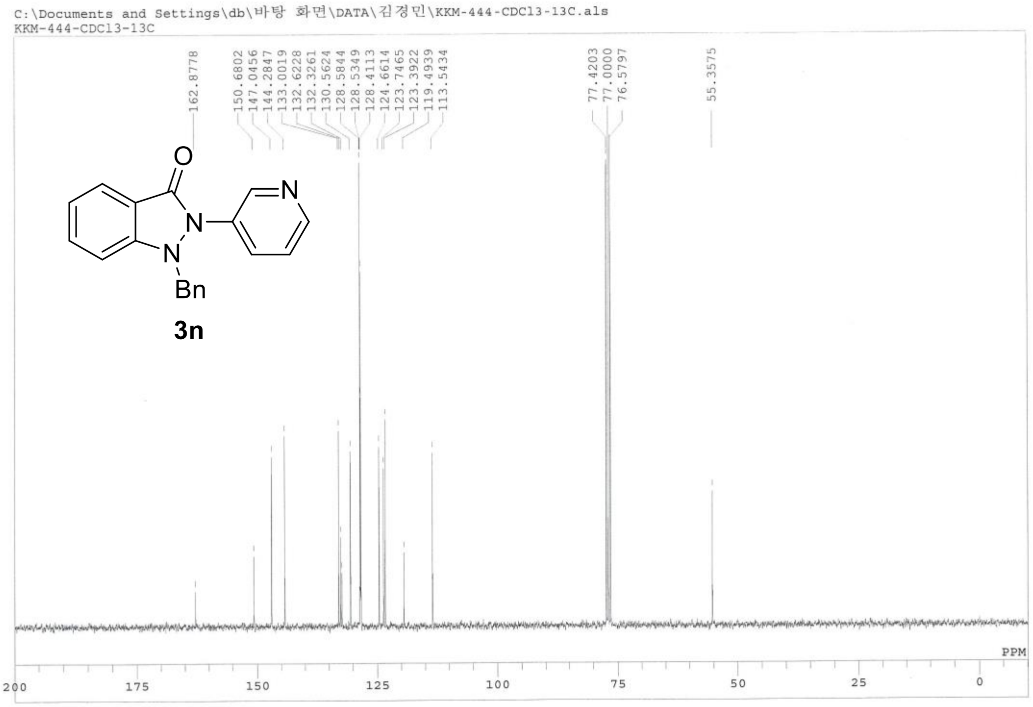

Figure S21. ^{1}H and ^{13}C NMR Spectra of Compounds **3o**.

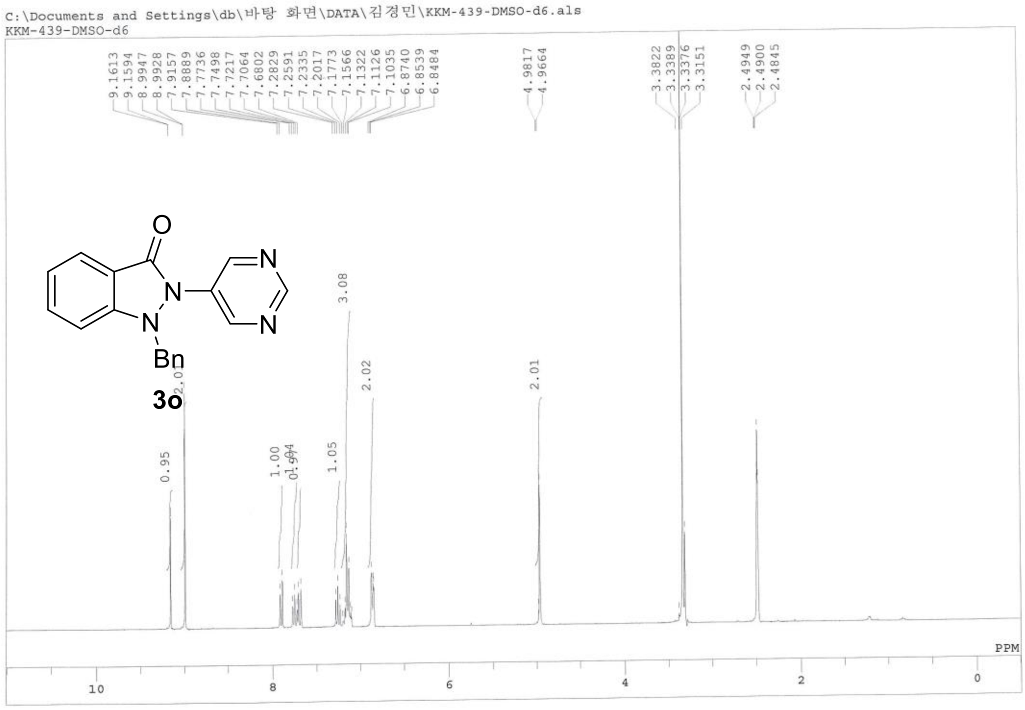

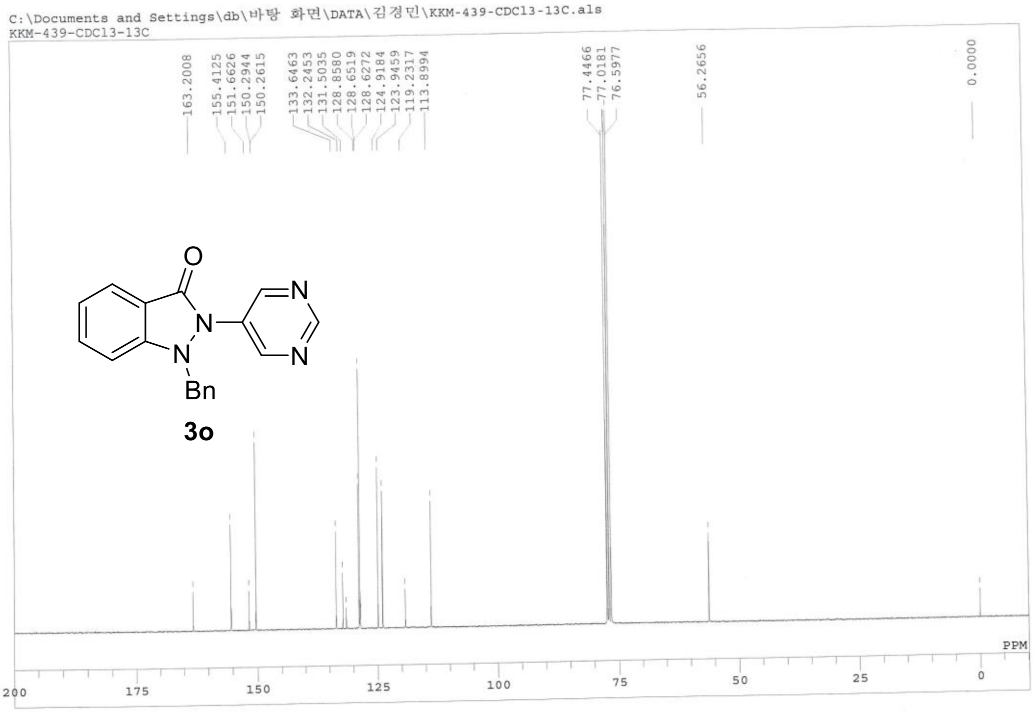

Figure S22. ^{1}H and ^{13}C NMR Spectra of Compounds **3a**.

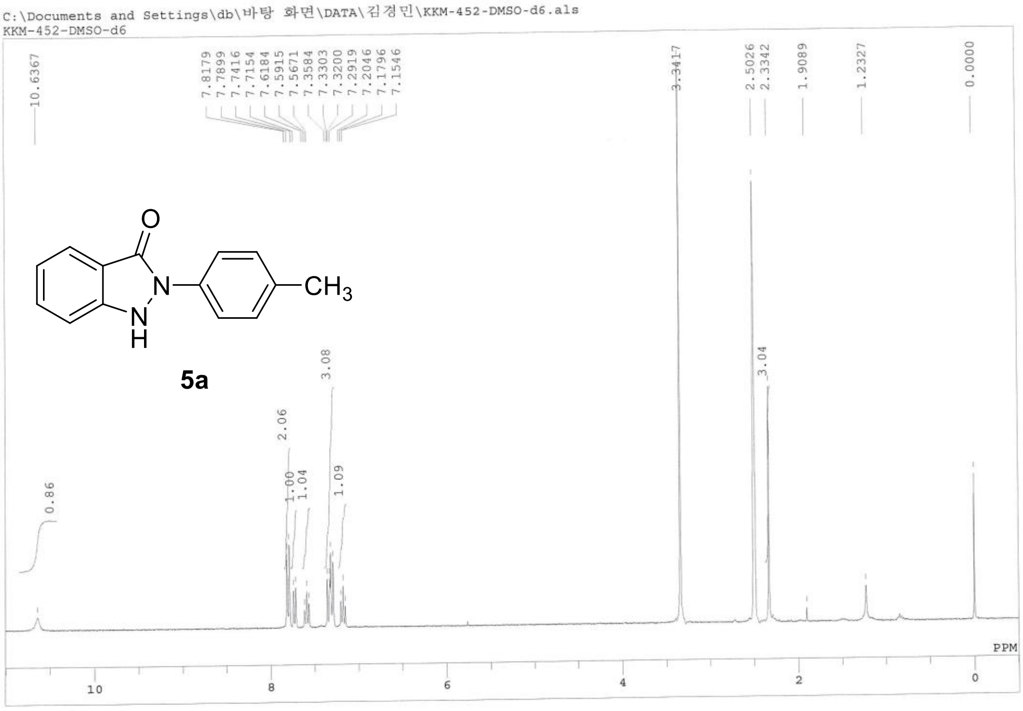

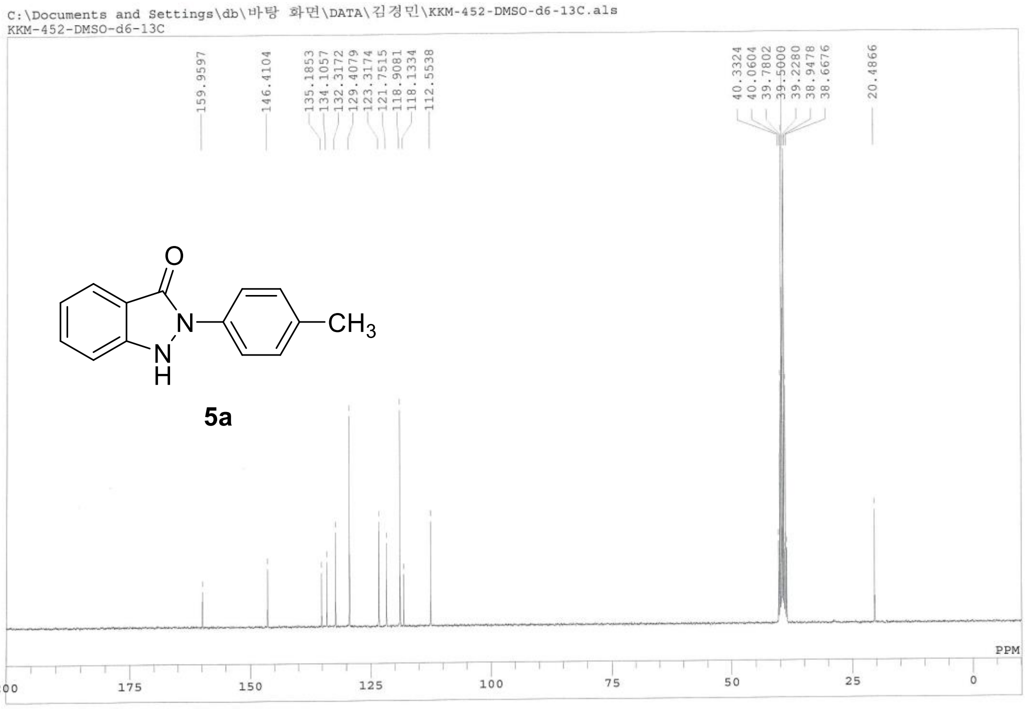

Figure S23. ^{1}H and ^{13}C NMR Spectra of Compounds **5b**.

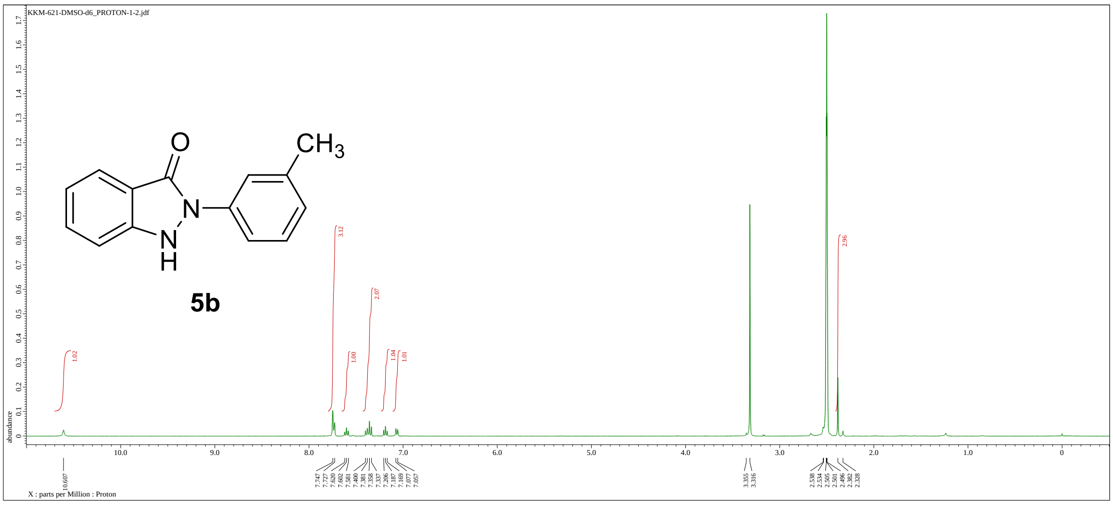

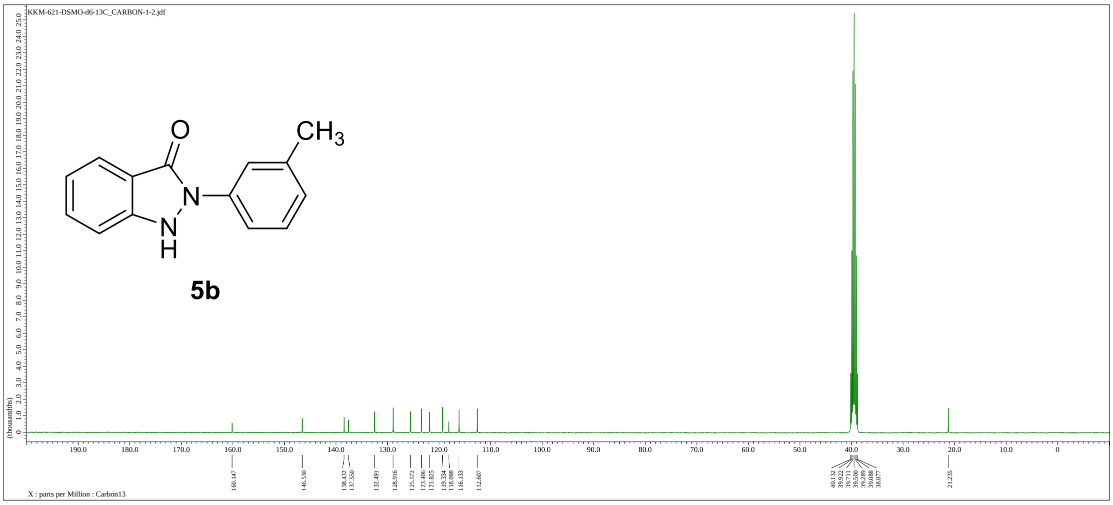

Figure S24. ^{1}H and ^{13}C NMR Spectra of Compounds **5c**.

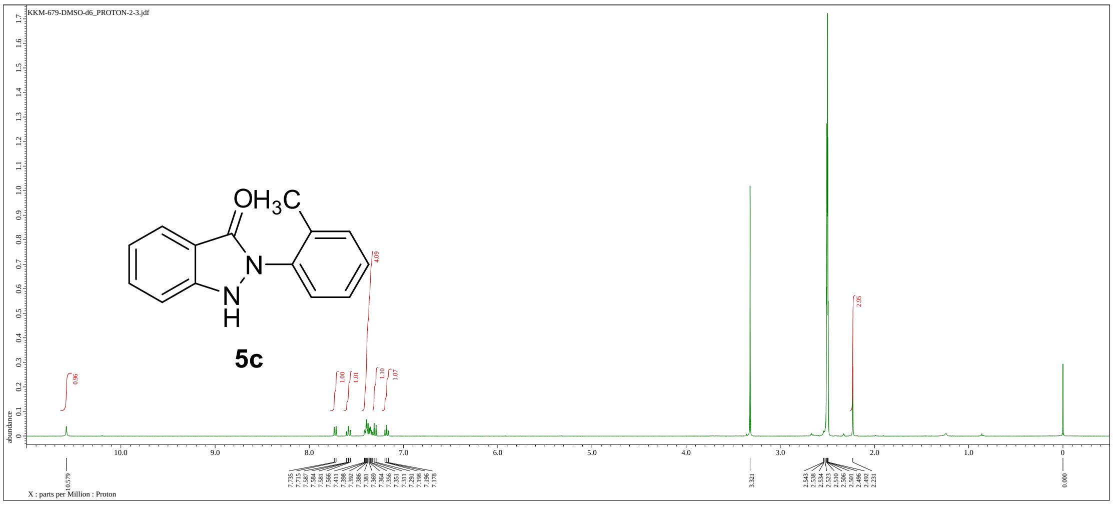

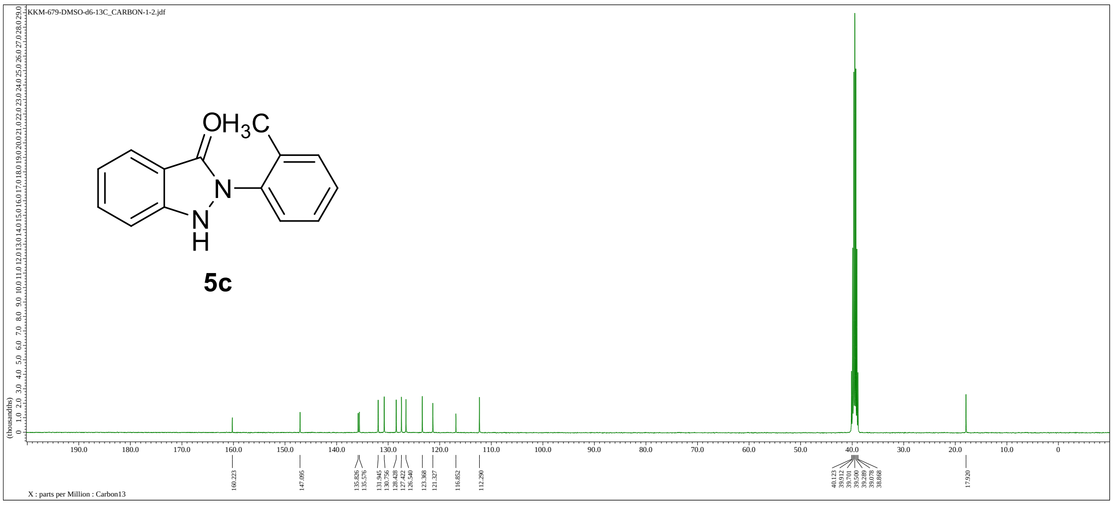

Figure S25. ^{1}H and ^{13}C NMR Spectra of Compounds **5d**.

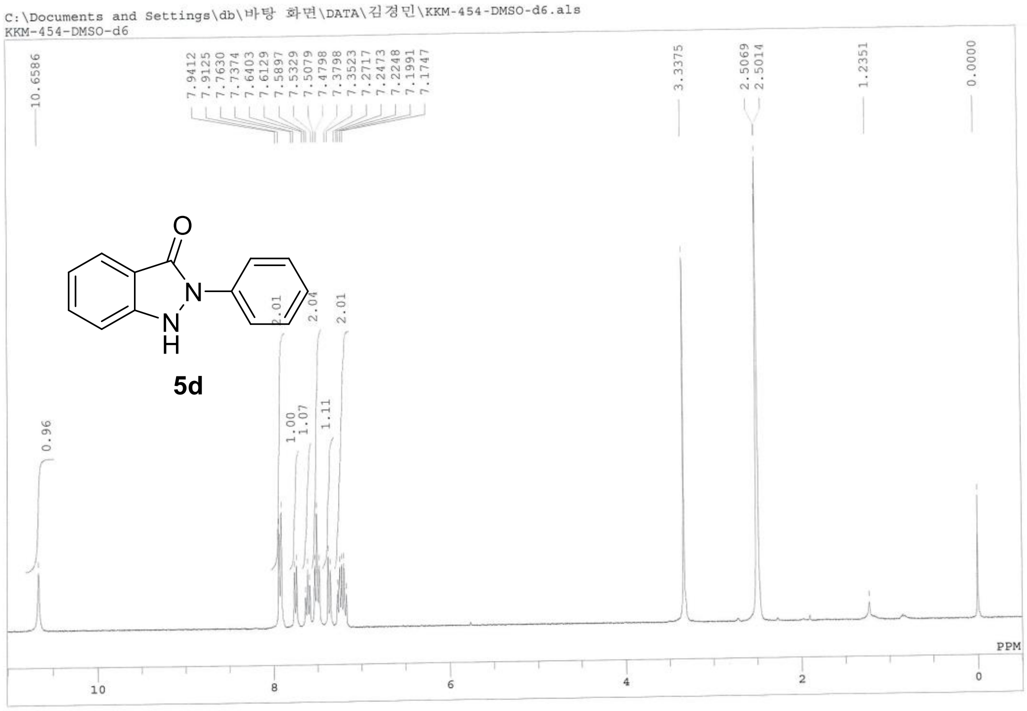

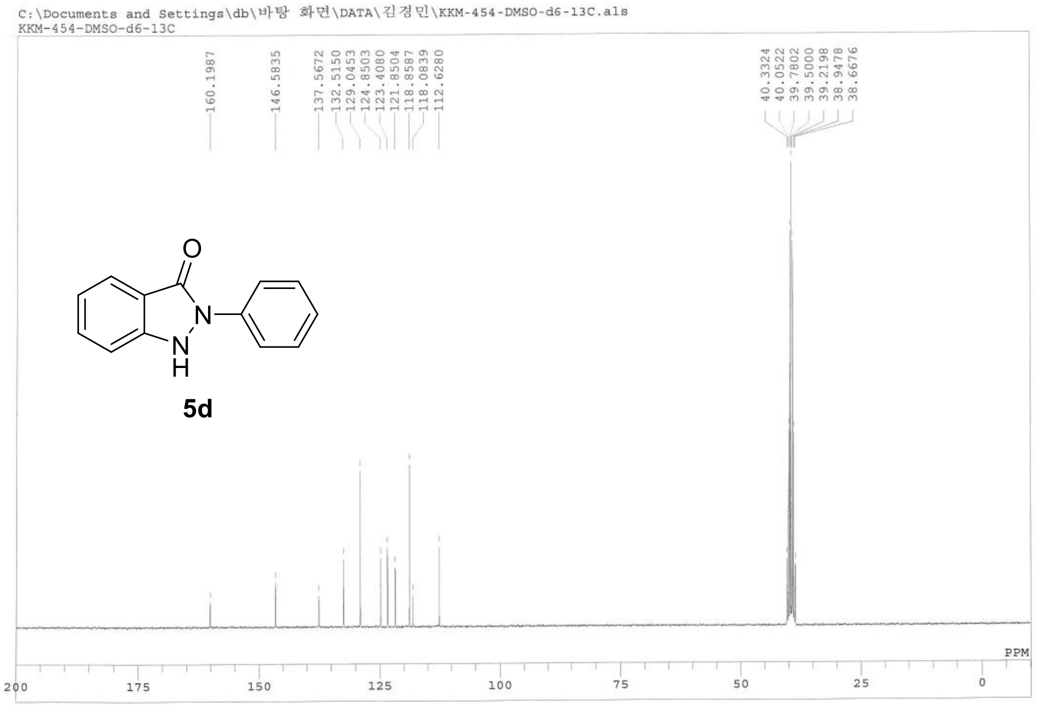

Figure S26. ^{1}H and ^{13}C NMR Spectra of Compounds **5e**.

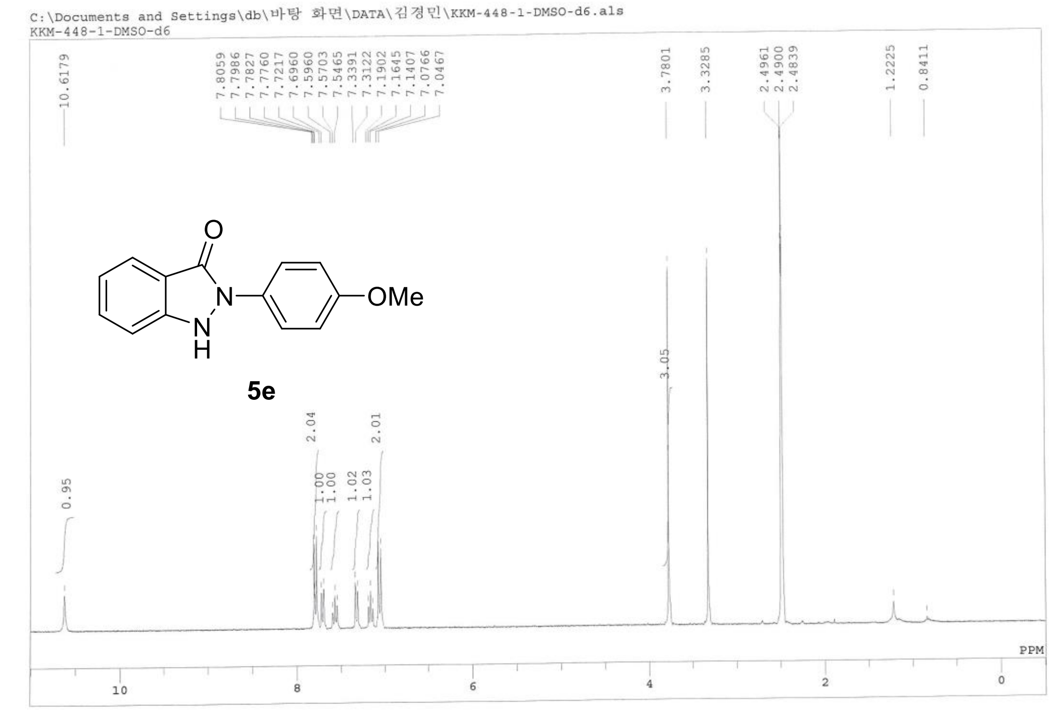

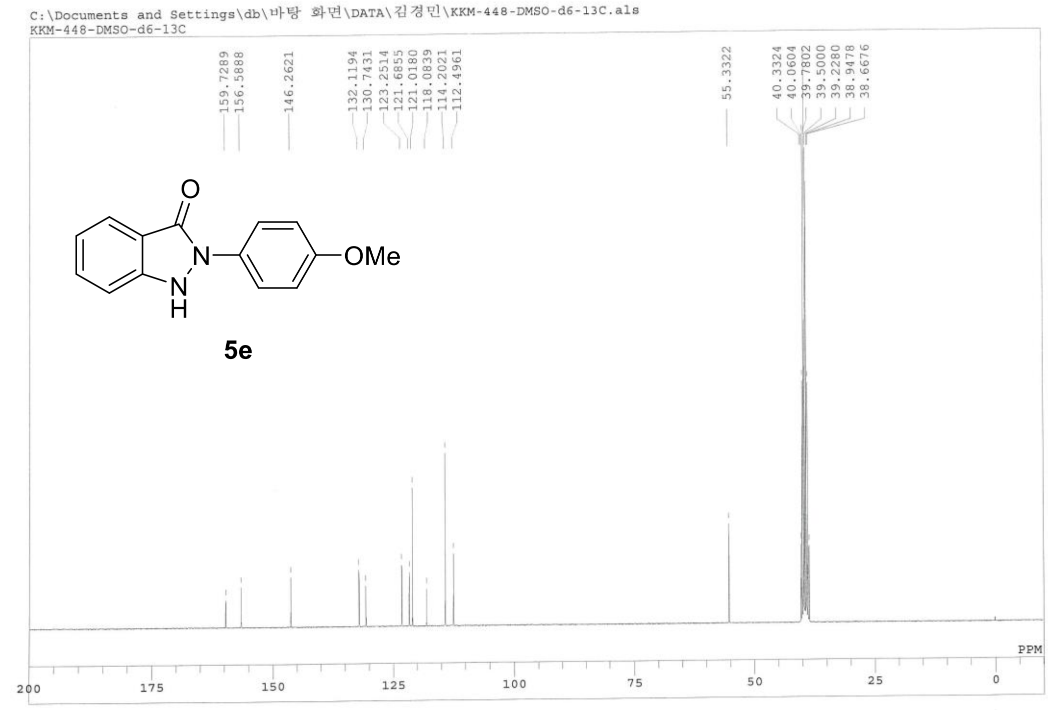

Figure S27. ^{1}H and ^{13}C NMR Spectra of Compounds **5f**.

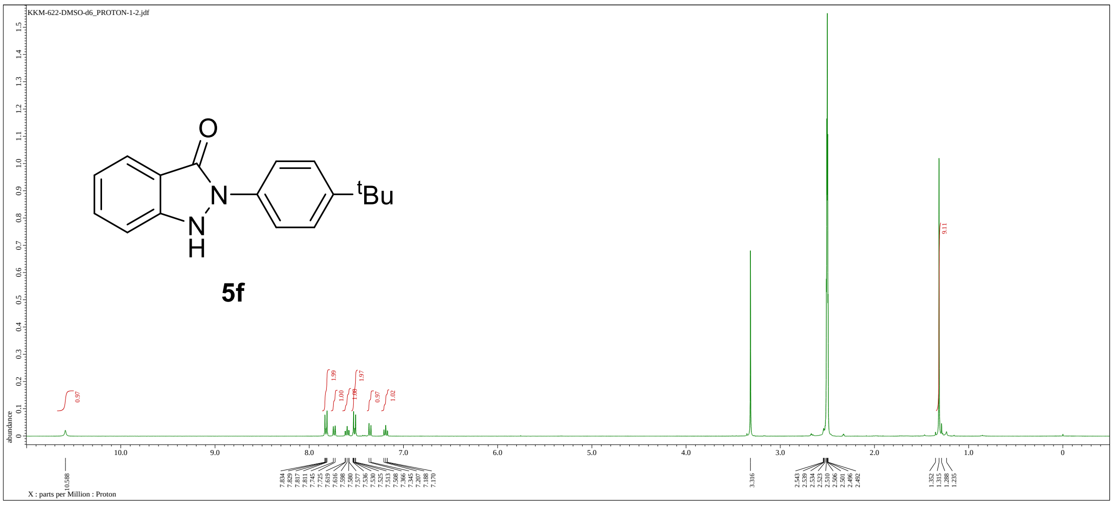

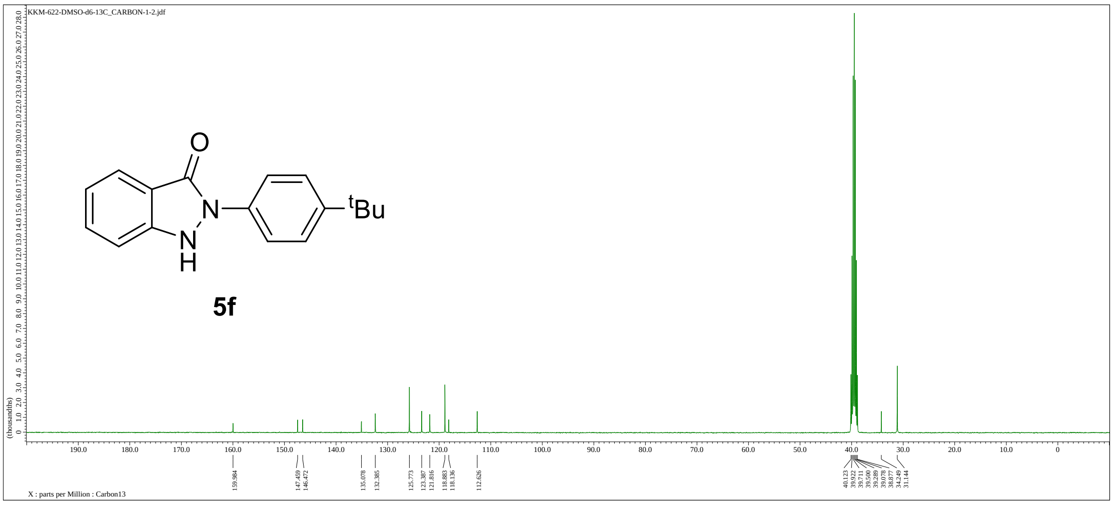

Figure S28. ^{1}H and ^{13}C NMR Spectra of Compounds **5g**.

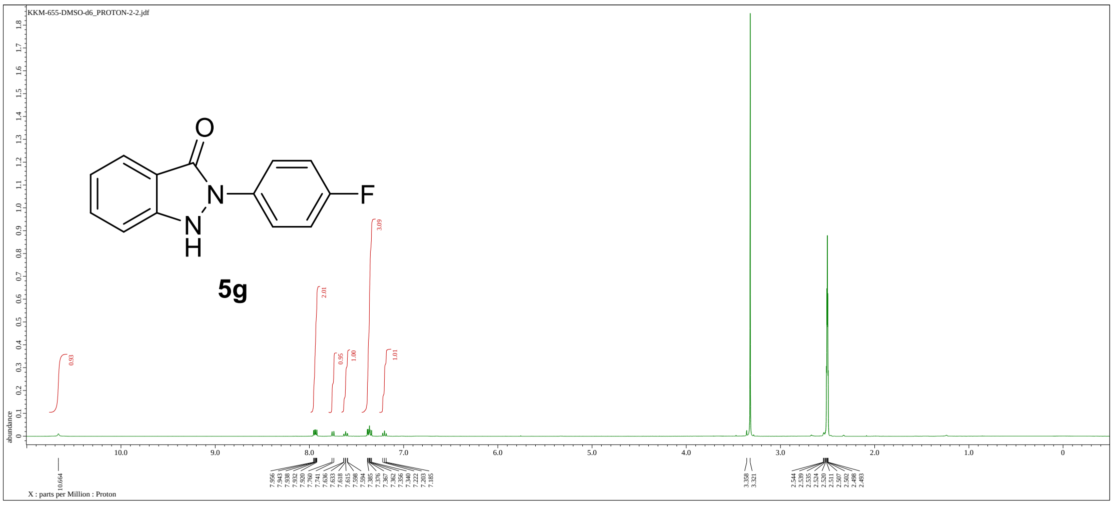

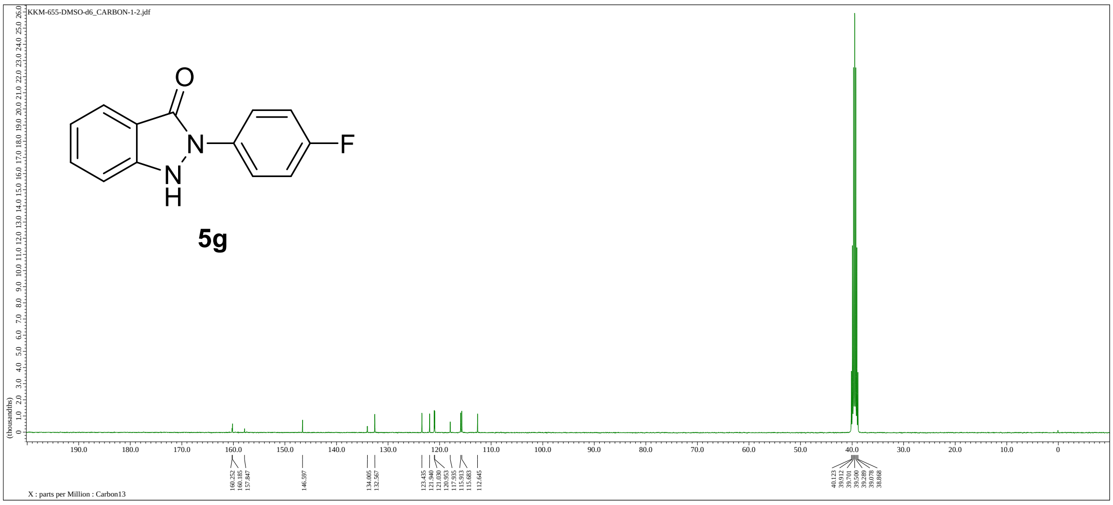

Figure S29. ^{1}H and ^{13}C NMR Spectra of Compounds **5h**.

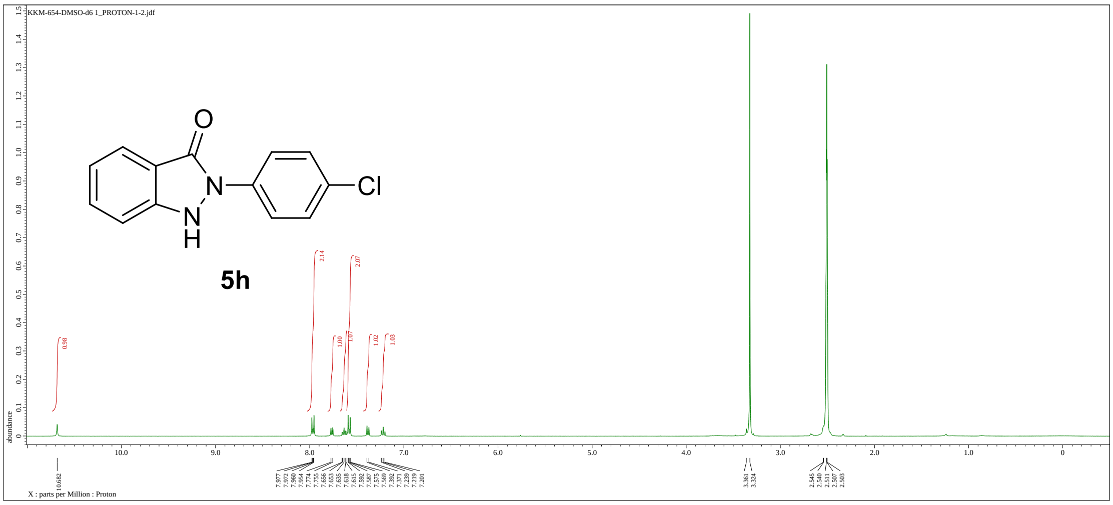

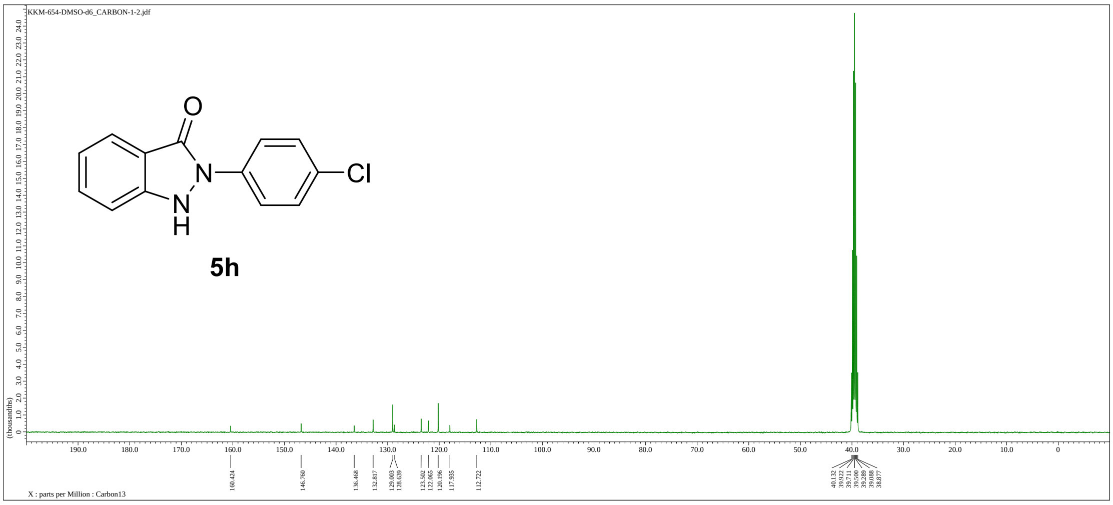

Figure S30. ^{1}H and ^{13}C NMR Spectra of Compounds **5i**.

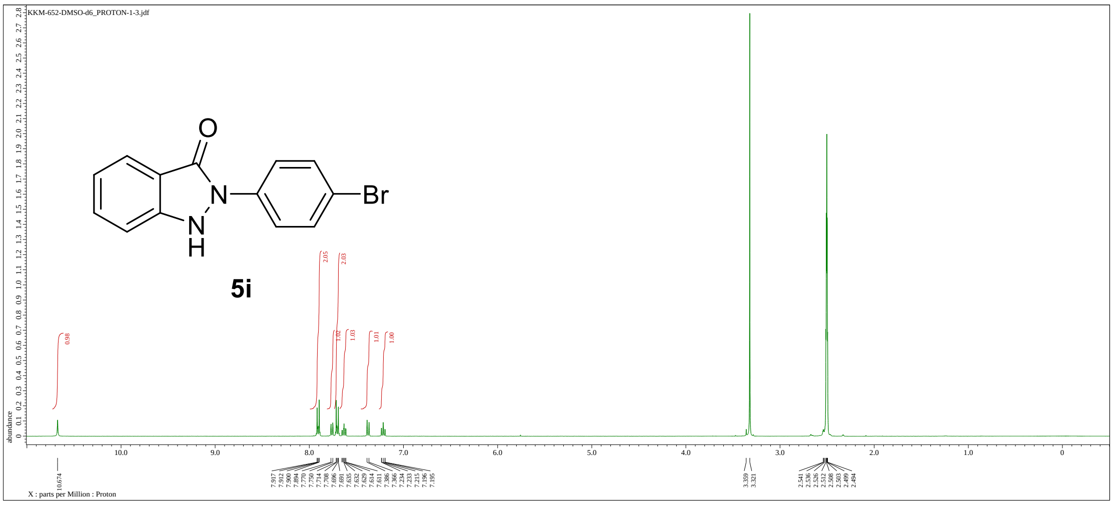

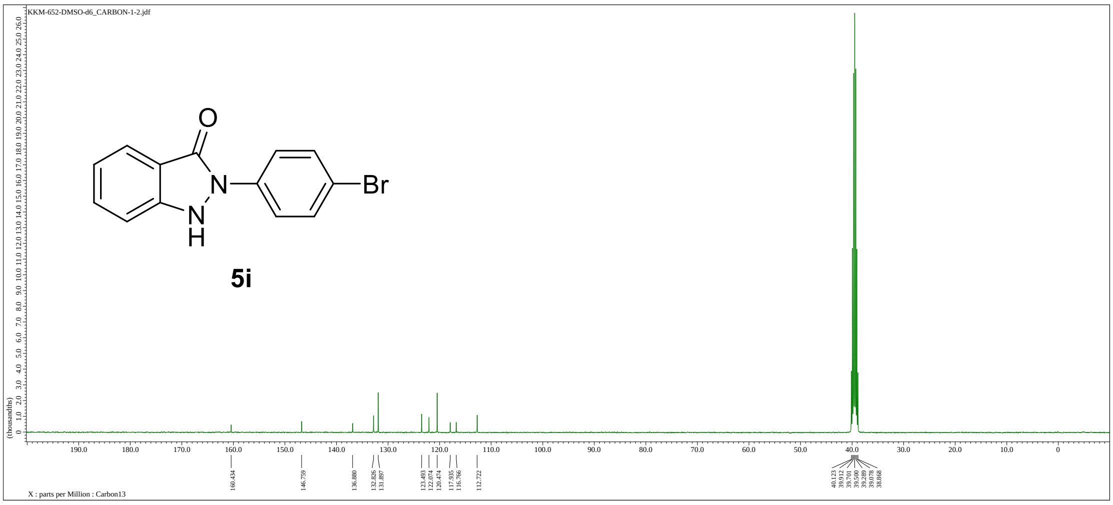

Figure S31. ^{1}H and ^{13}C NMR Spectra of Compounds **5j**.

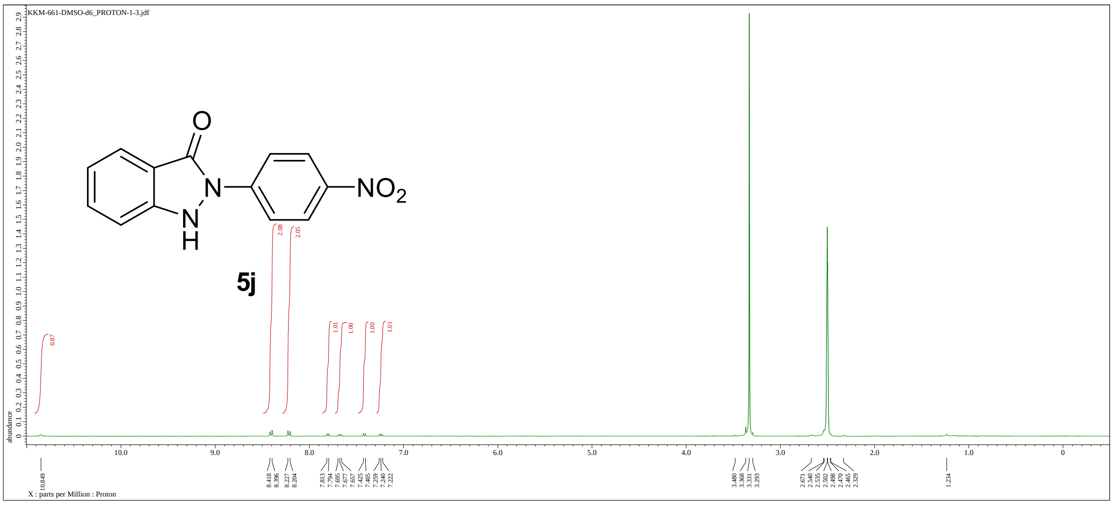

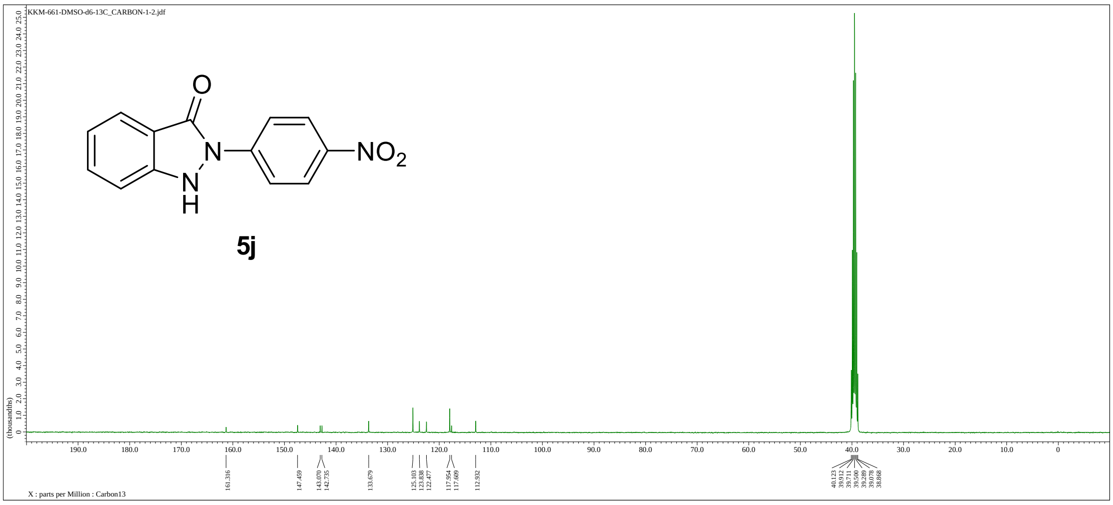

Figure S32. ^{1}H and ^{13}C NMR Spectra of Compounds **5k**.

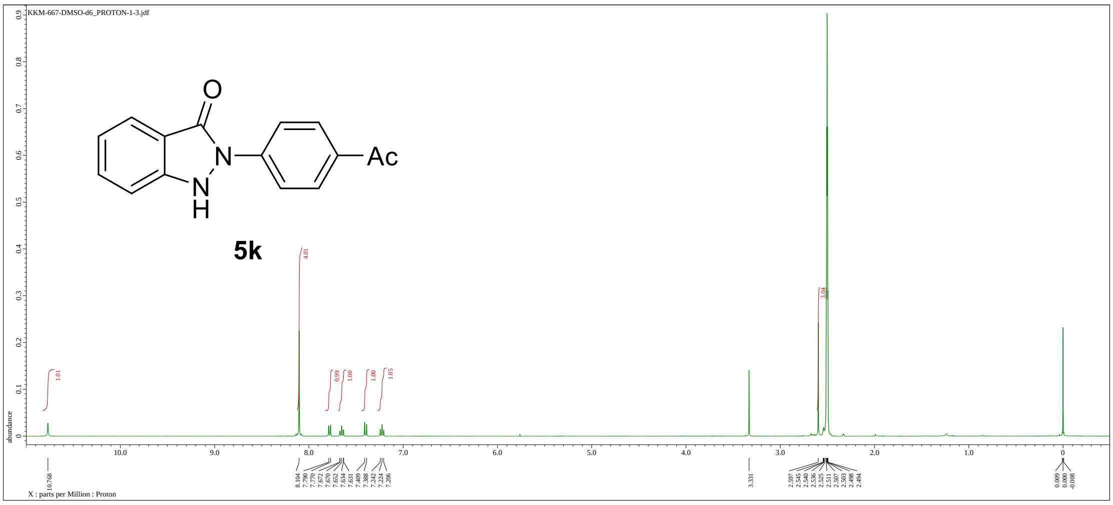

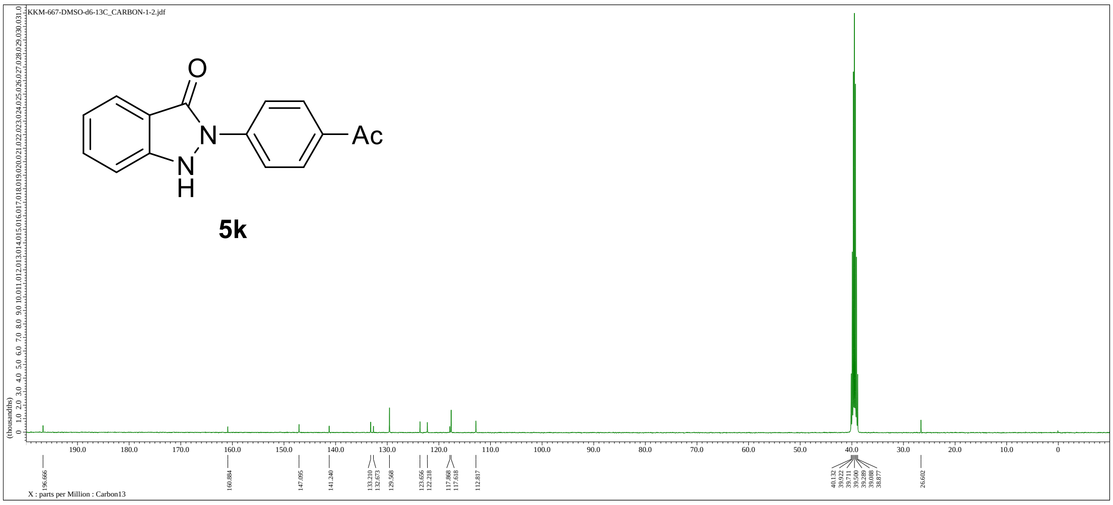

Figure S33. ^{1}H and ^{13}C NMR Spectra of Compounds **5l**.

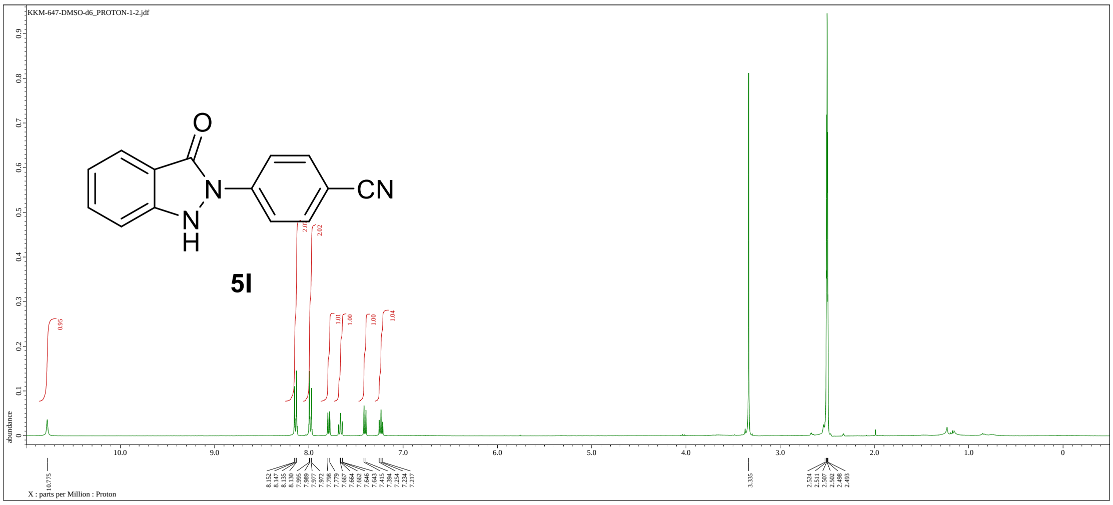

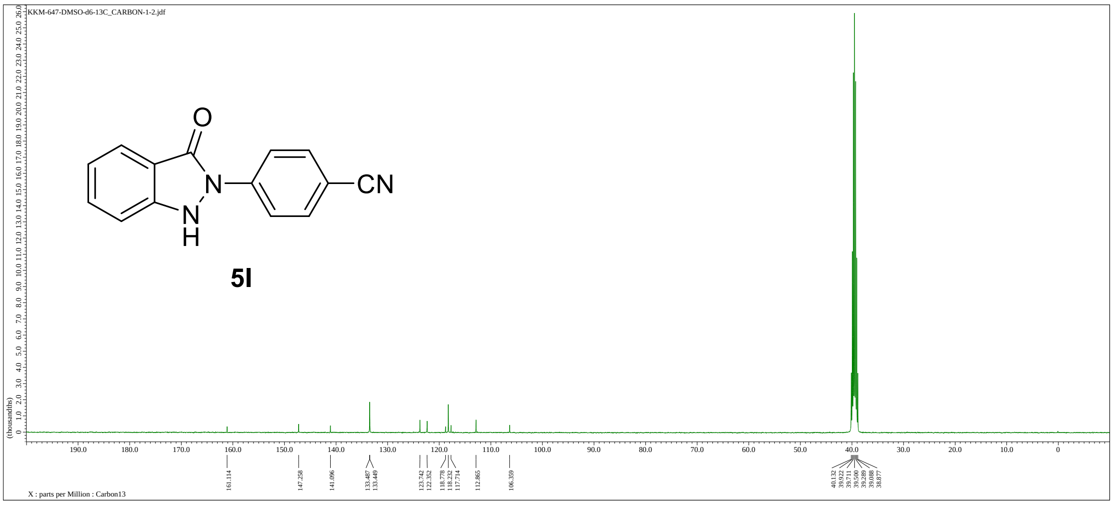

Figure S34. ^{1}H and ^{13}C NMR Spectra of Compounds **5m**.

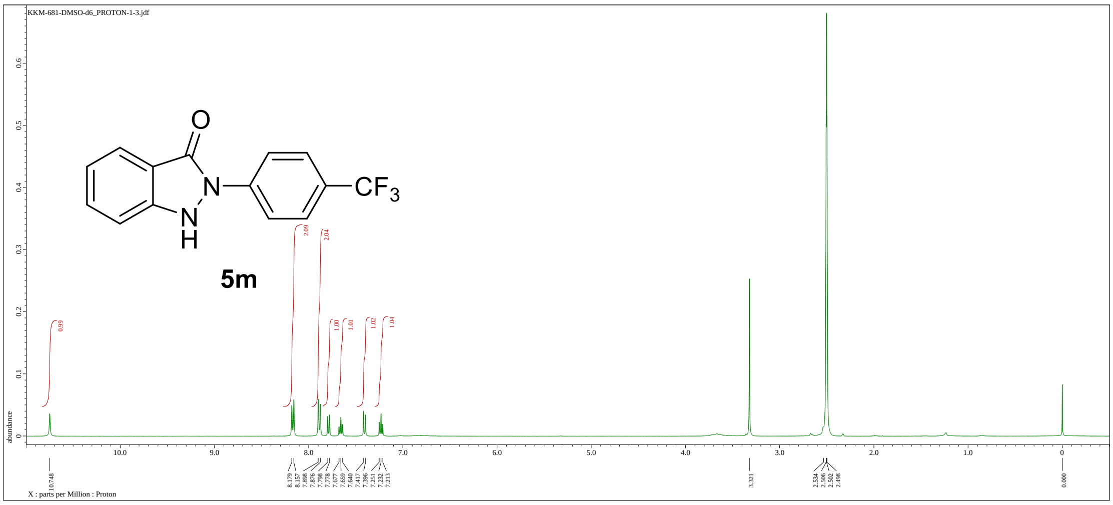

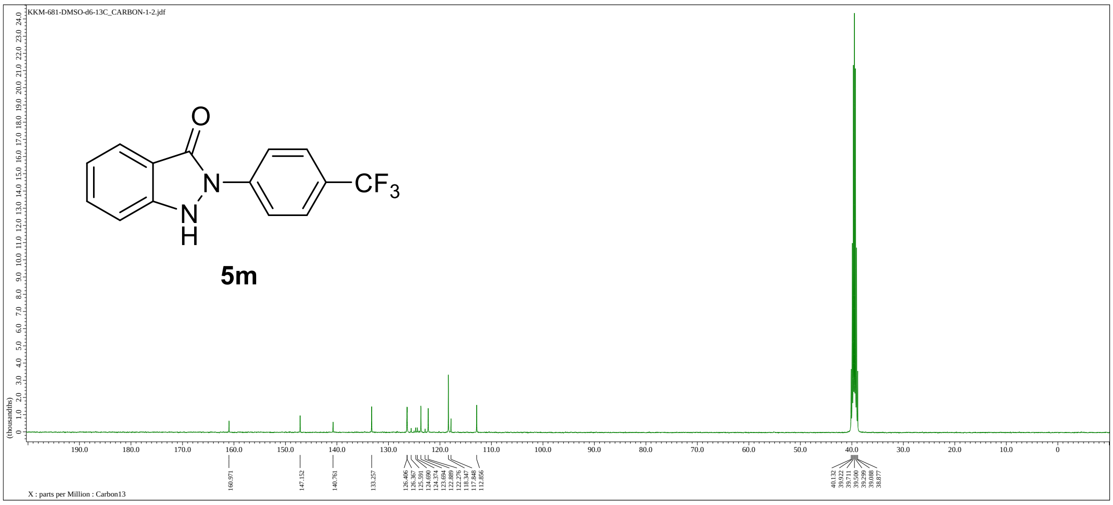

Figure S35. ^{1}H and ^{13}C NMR Spectra of Compounds **5n**.

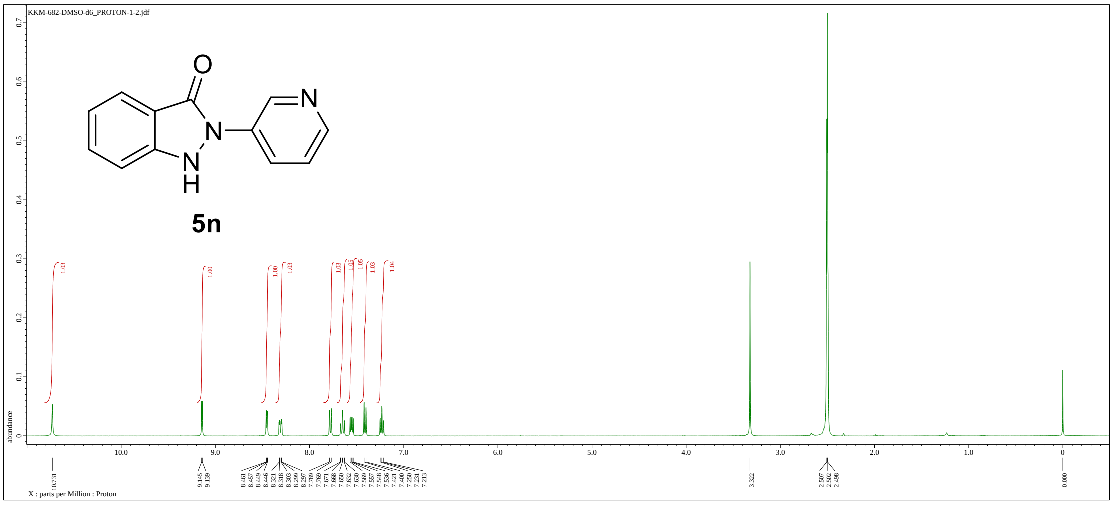

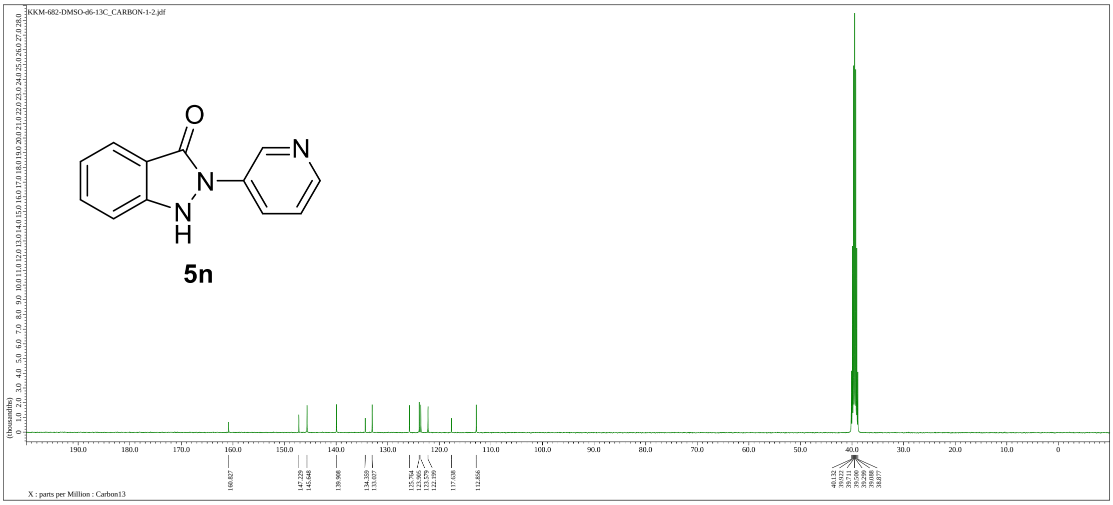

Figure S36. ^{1}H and ^{13}C NMR Spectra of Compounds **5o**.

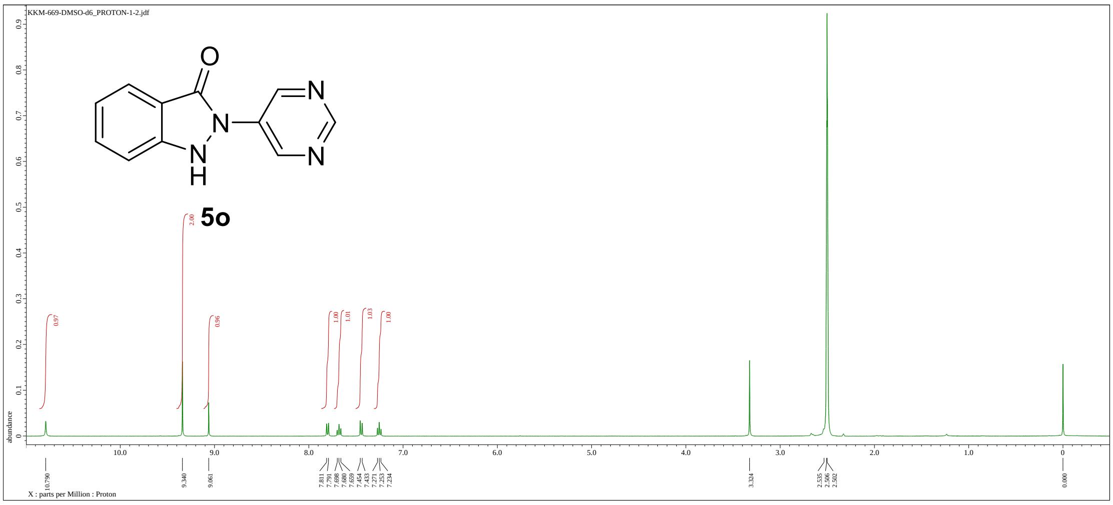

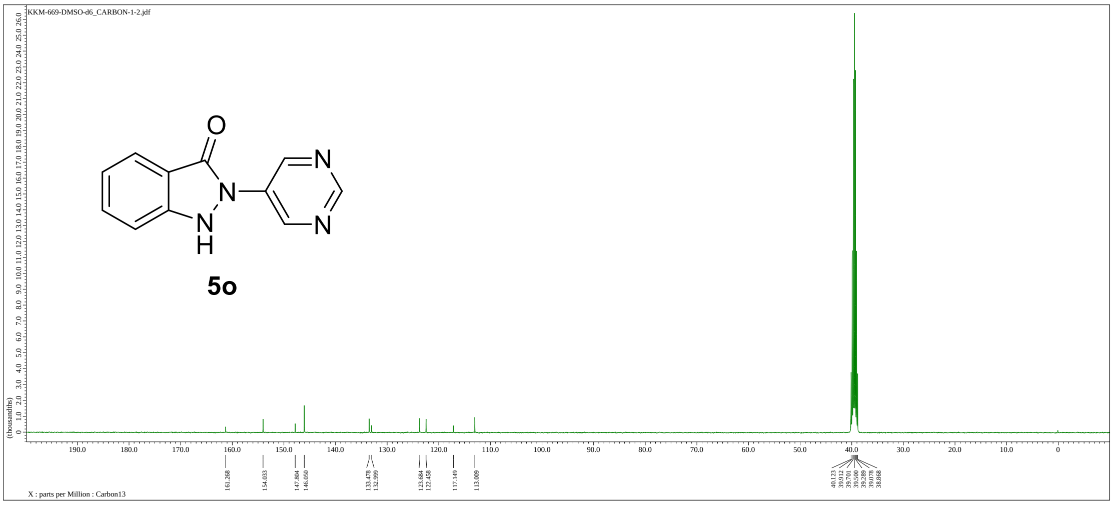

Figure S37. ^{1}H and ^{13}C NMR Spectra of Compounds **6**.

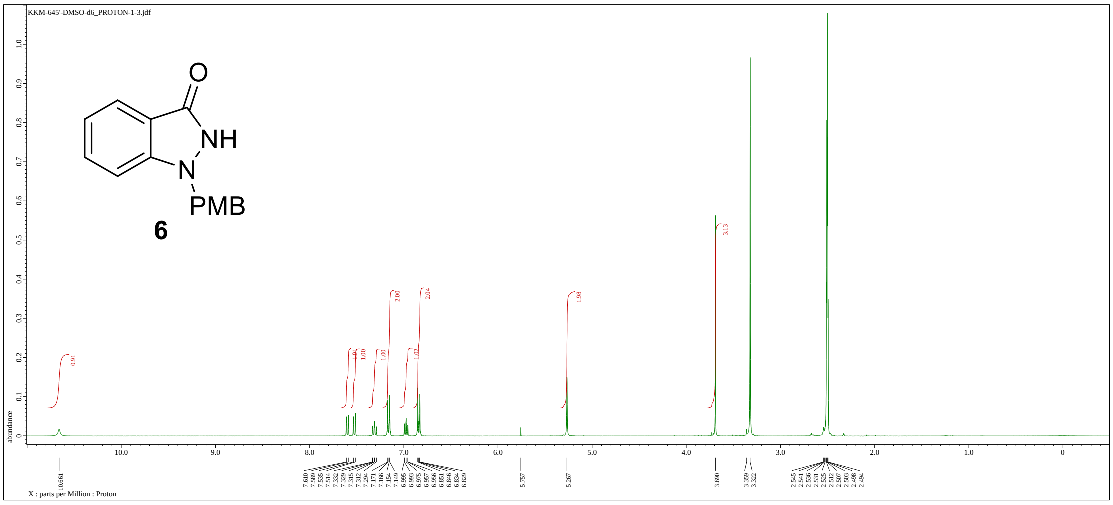

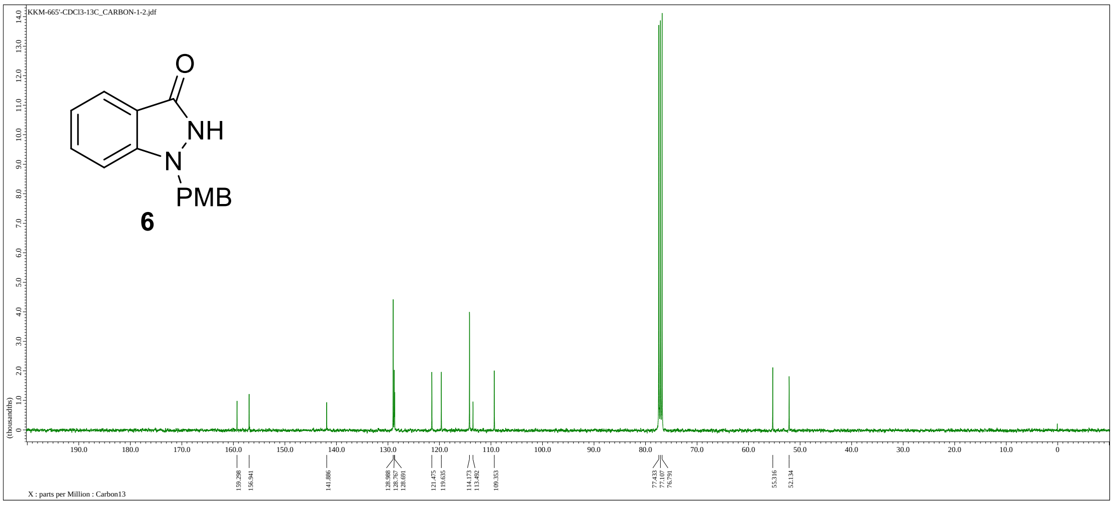

Figure S38. ^{1}H and ^{13}C NMR Spectra of Compounds **7c**.

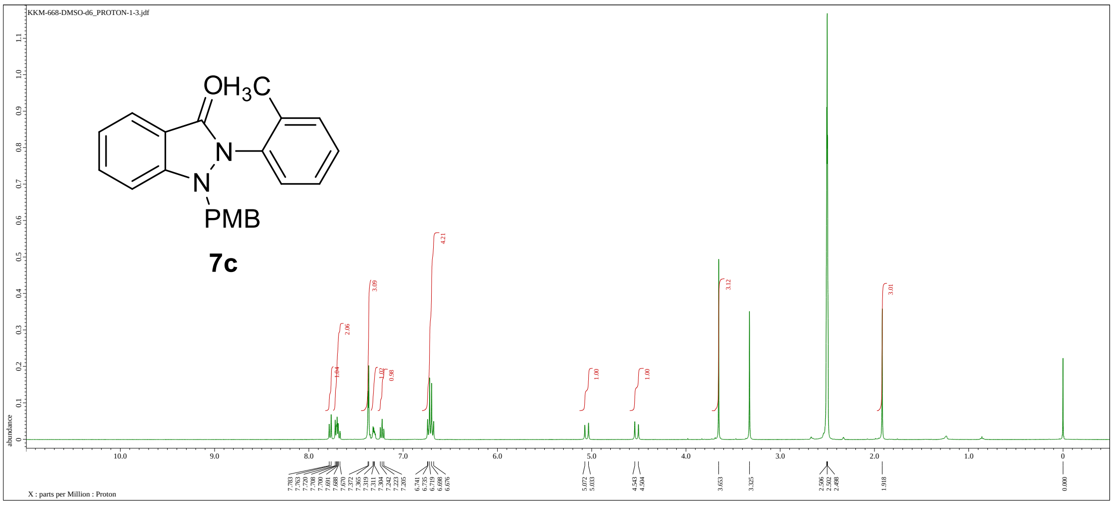

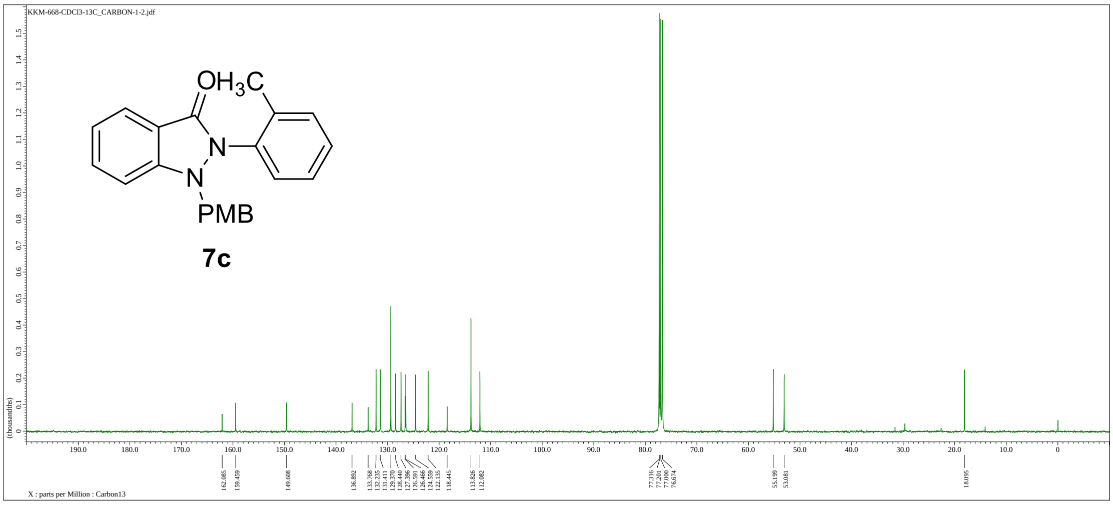

Figure S39. ^{1}H and ^{13}C NMR Spectra of Compounds **7g**.

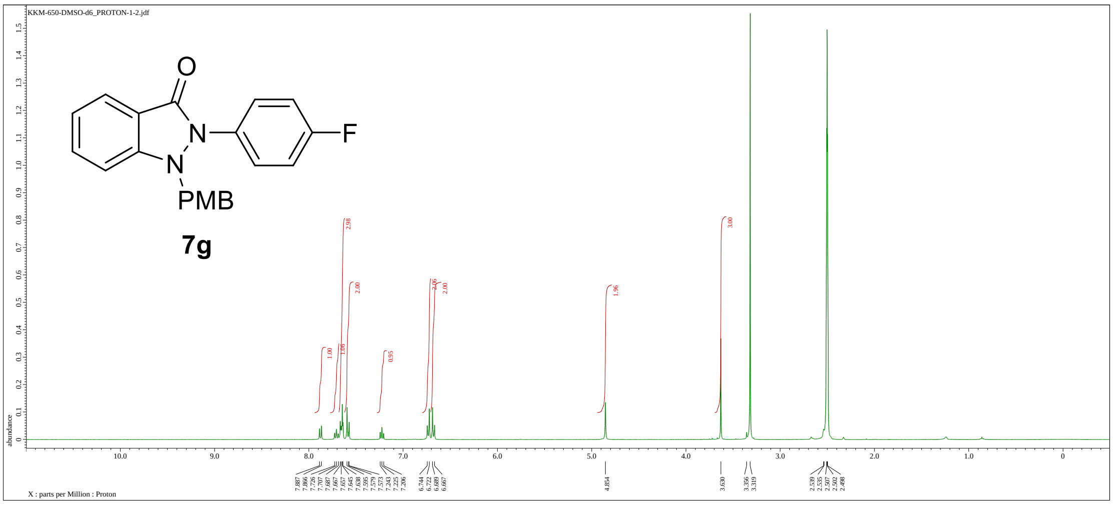

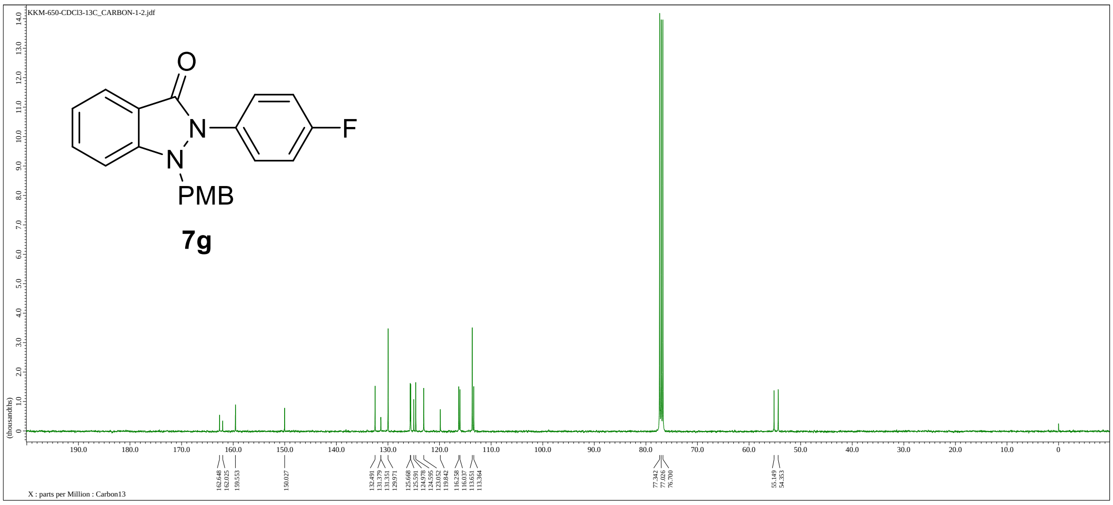

Figure S40. ^{1}H and ^{13}C NMR Spectra of Compounds **7h**.

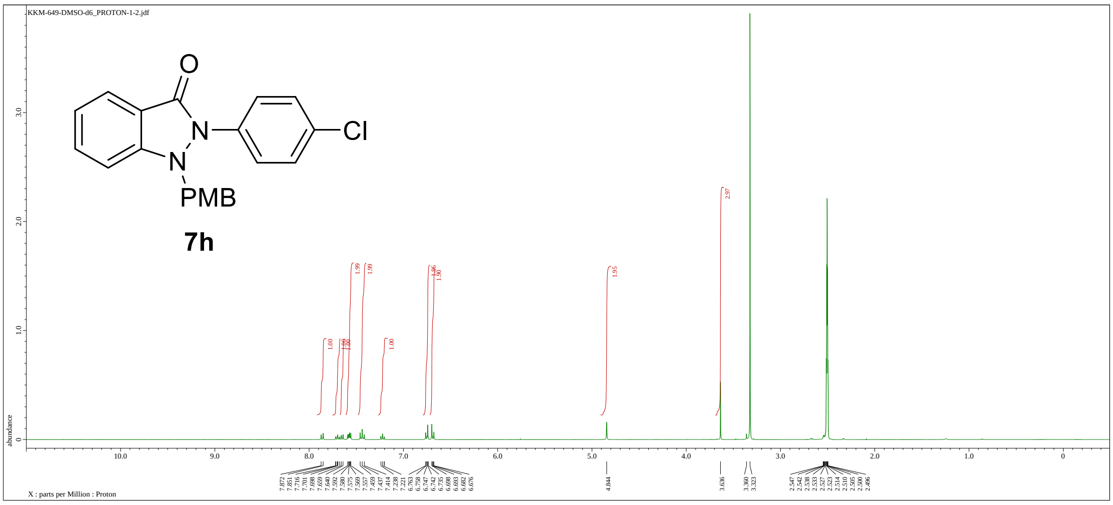

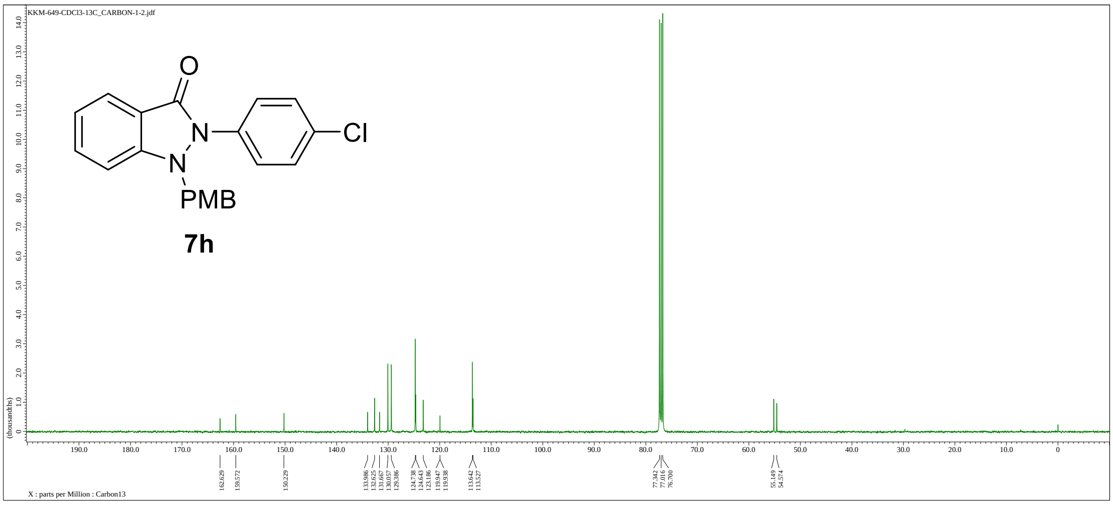

Figure S41. ^{1}H and ^{13}C NMR Spectra of Compounds **7i**.

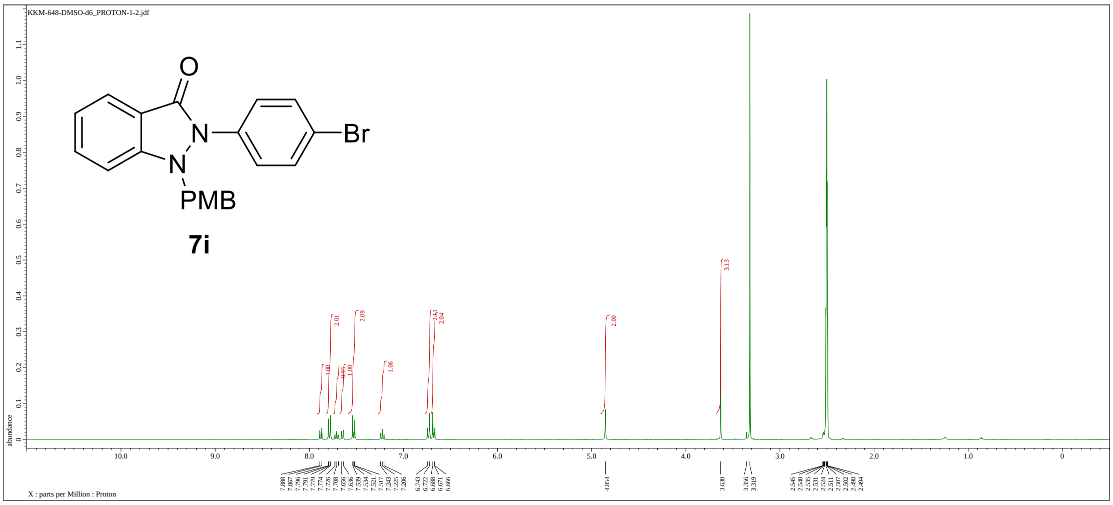

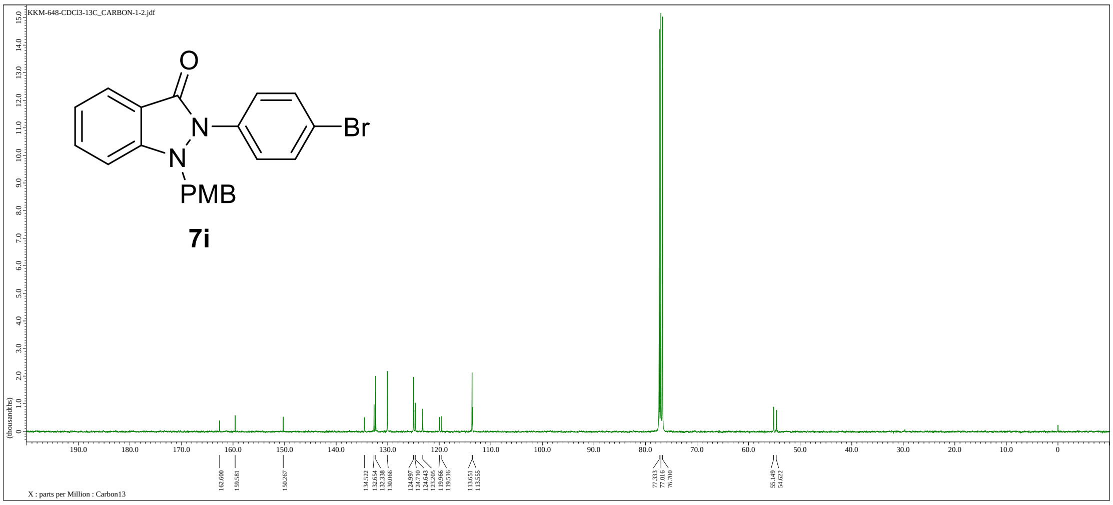

Figure S42. ^{1}H and ^{13}C NMR Spectra of Compounds **7j**.

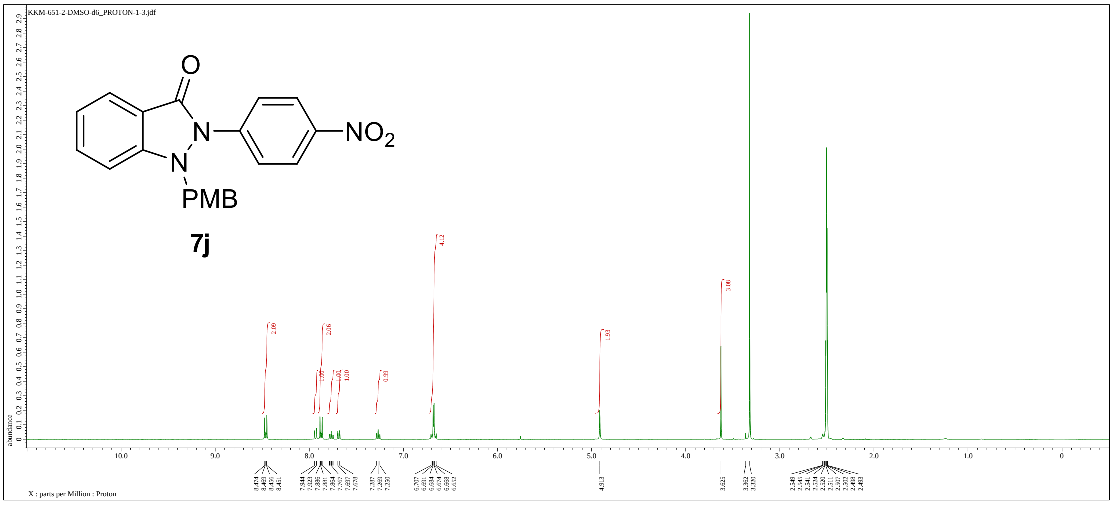

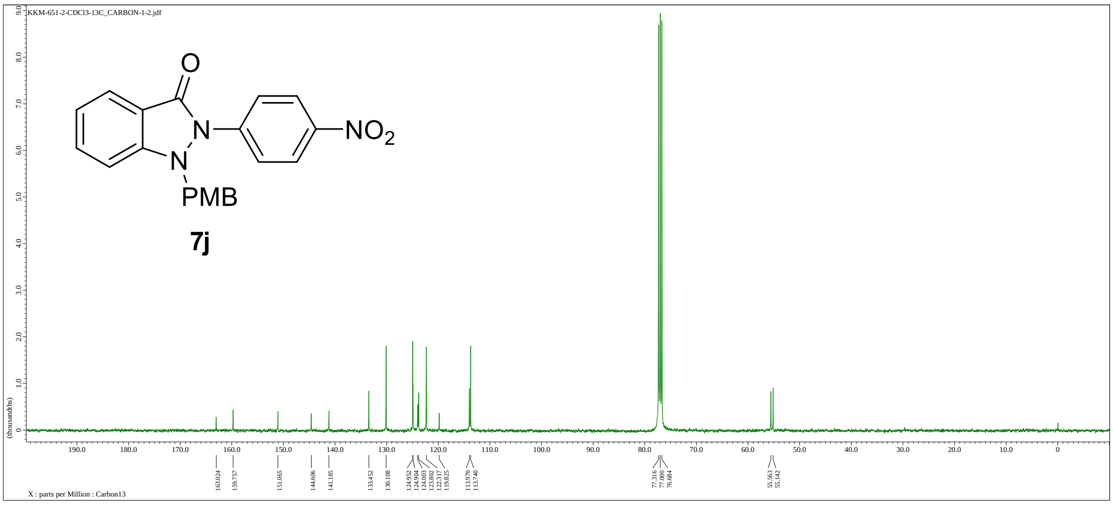

Figure S43. ^{1}H and ^{13}C NMR Spectra of Compounds **8j**.

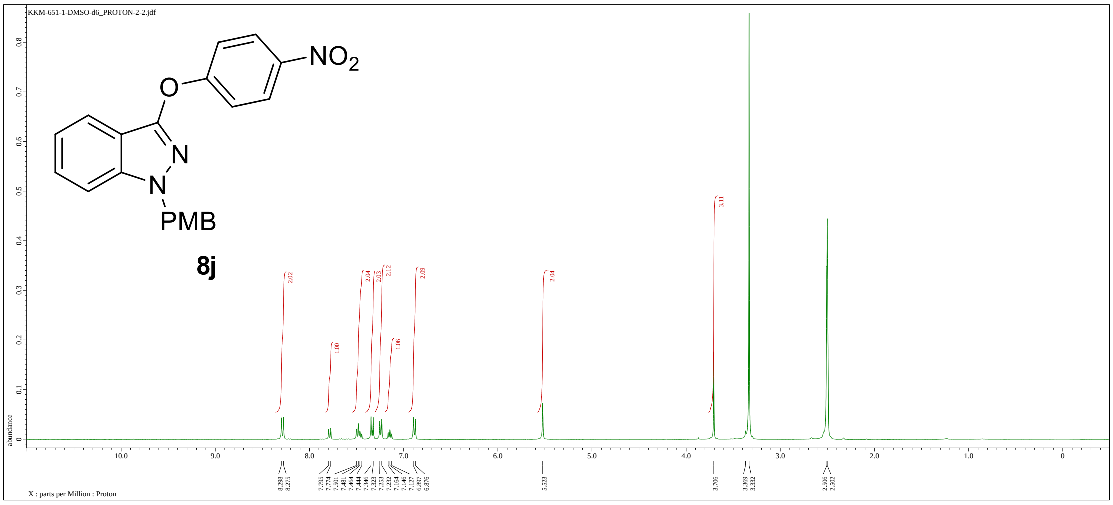

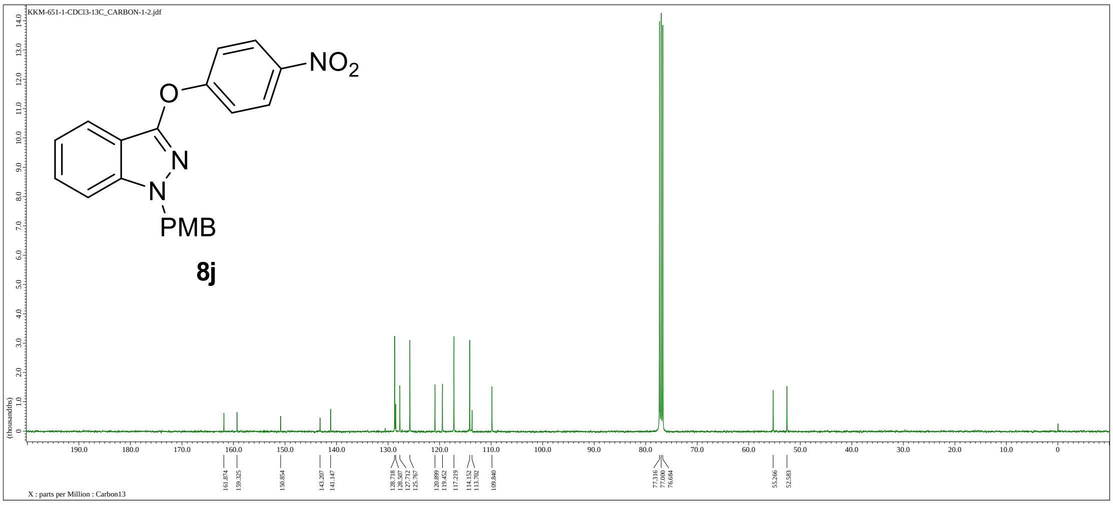

Figure S44. ^{1}H and ^{13}C NMR Spectra of Compounds **7k**.

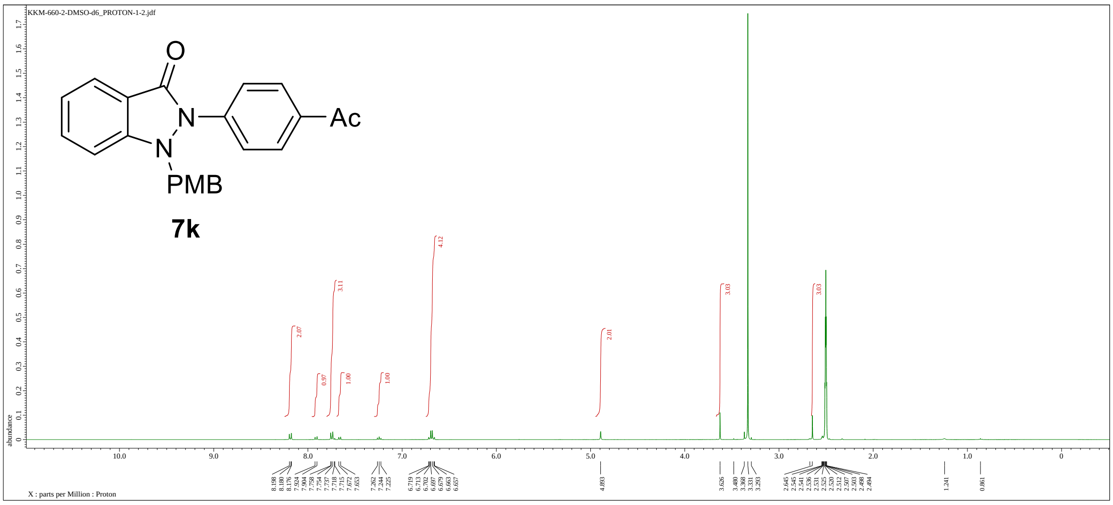

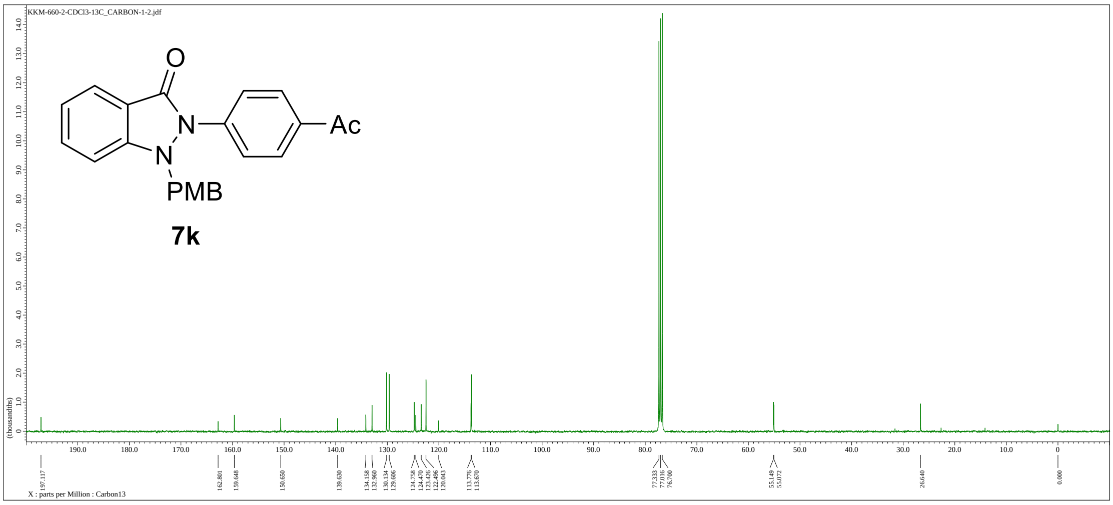

Figure S45. ^{1}H and ^{13}C NMR Spectra of Compounds **8k**.

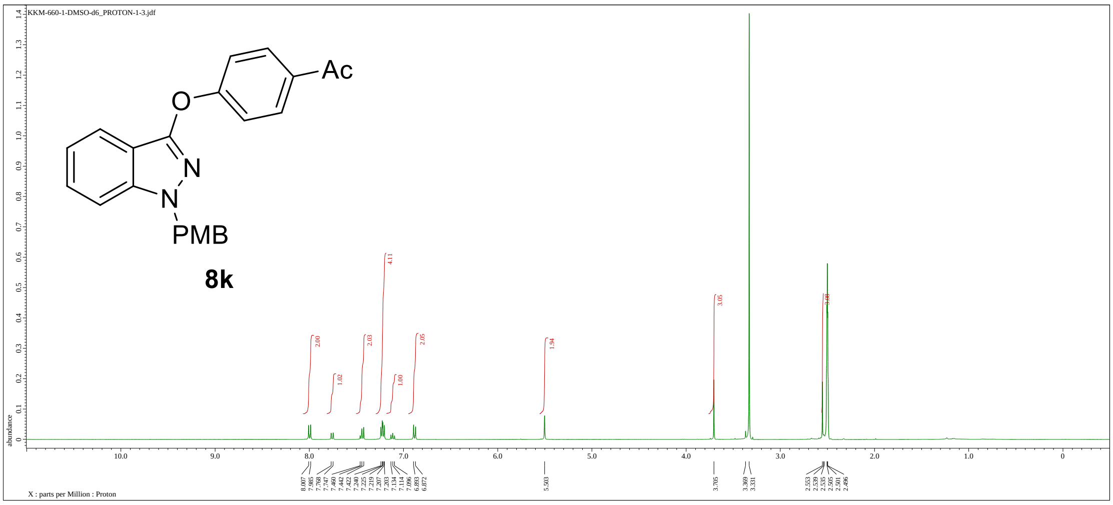

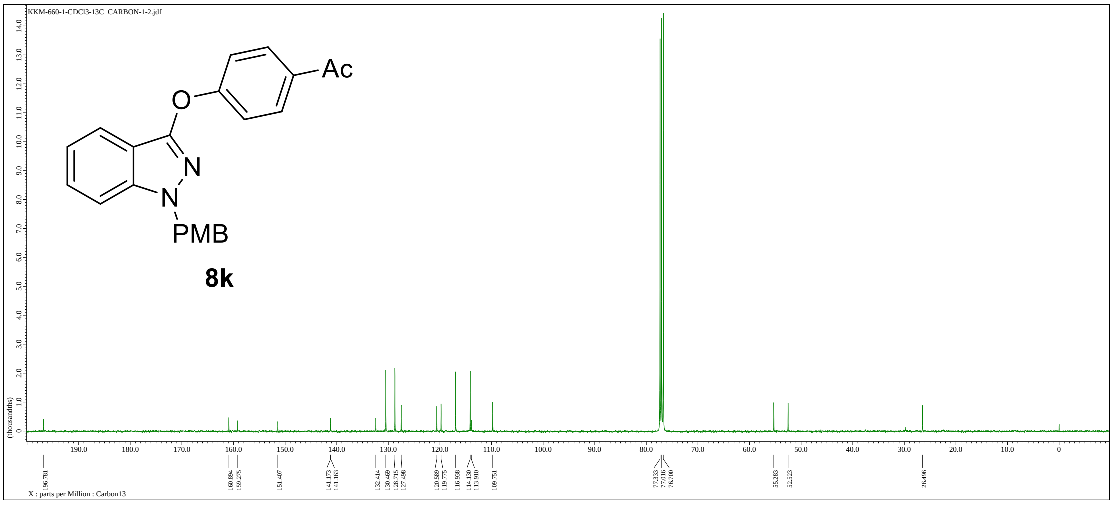

Figure S46. ^{1}H and ^{13}C NMR Spectra of Compounds **7l**.

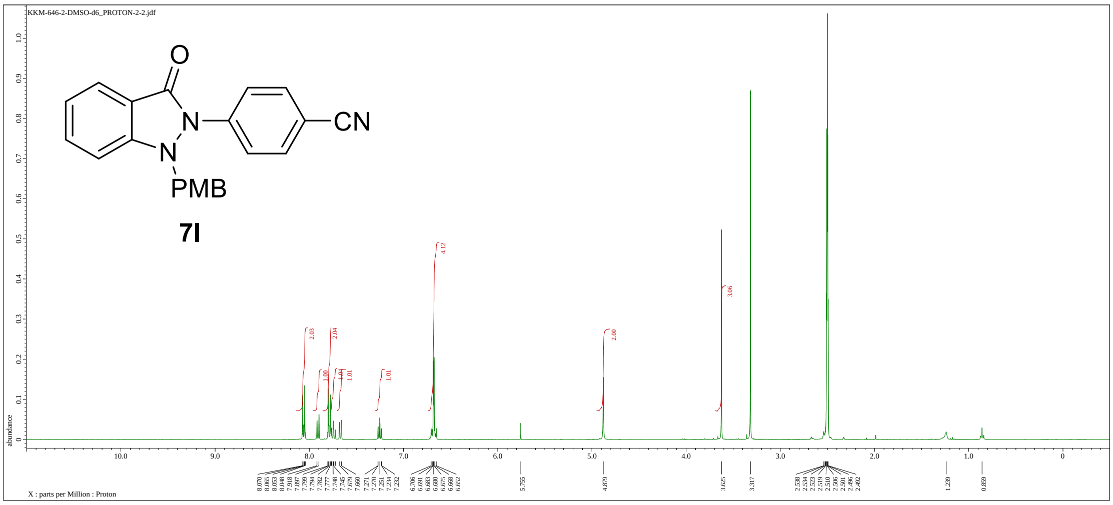

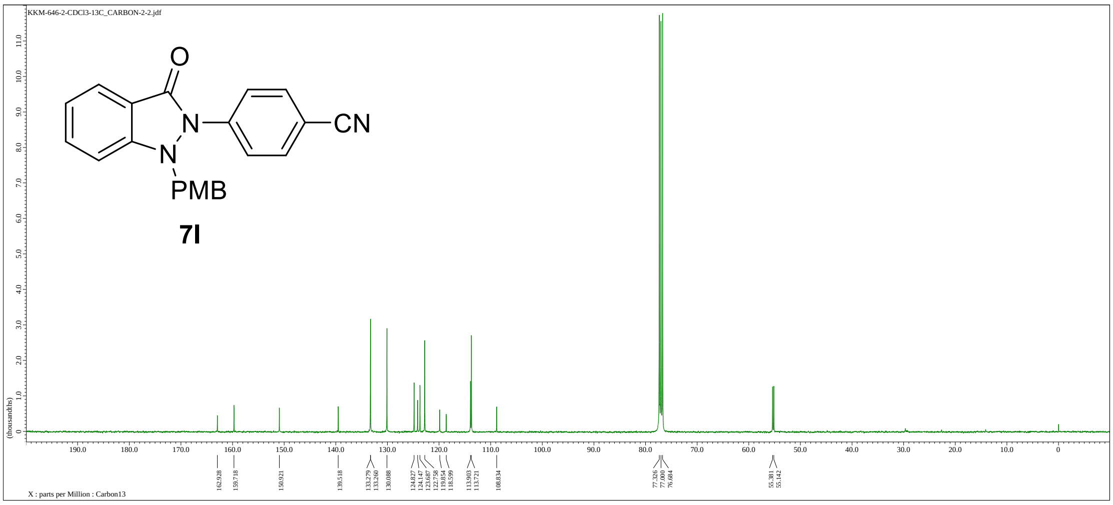

Figure S47. ^{1}H and ^{13}C NMR Spectra of Compounds **8l**.

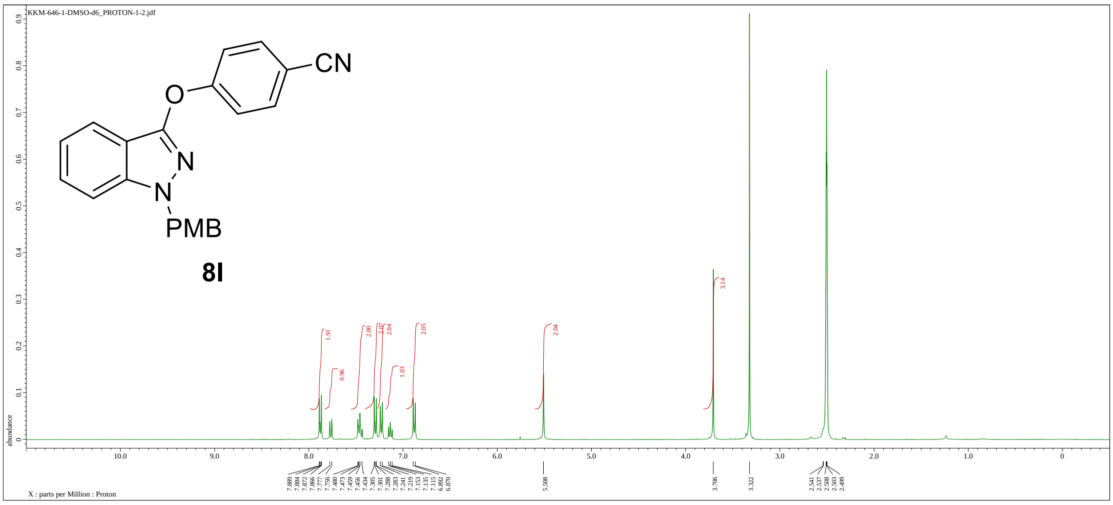

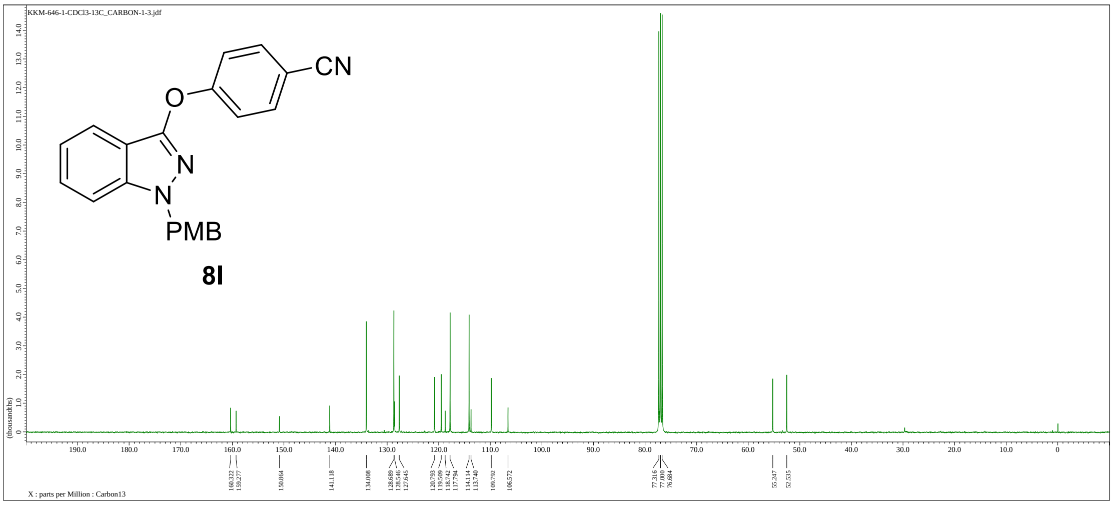

Figure S48. ^{1}H and ^{13}C NMR Spectra of Compounds **7m**.

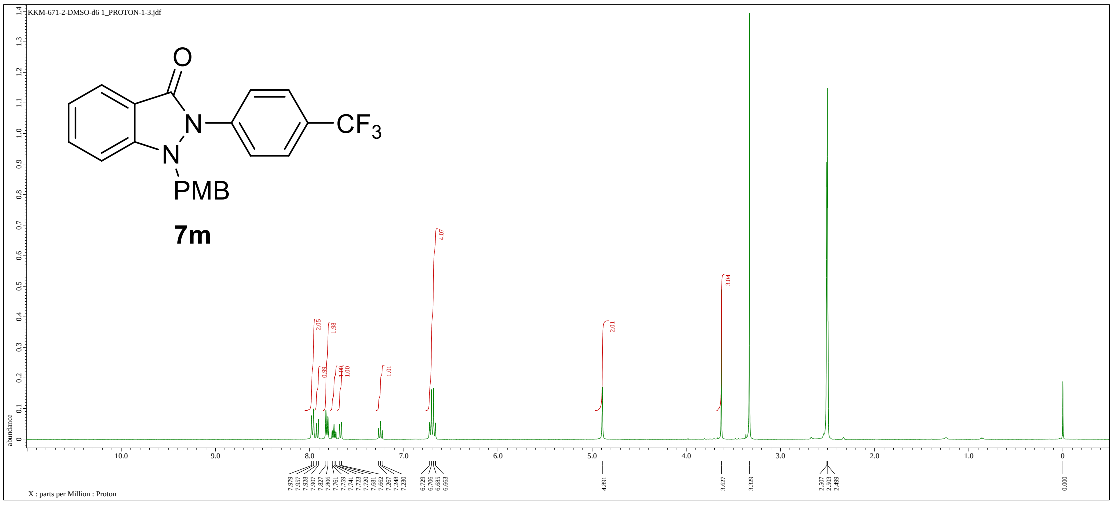

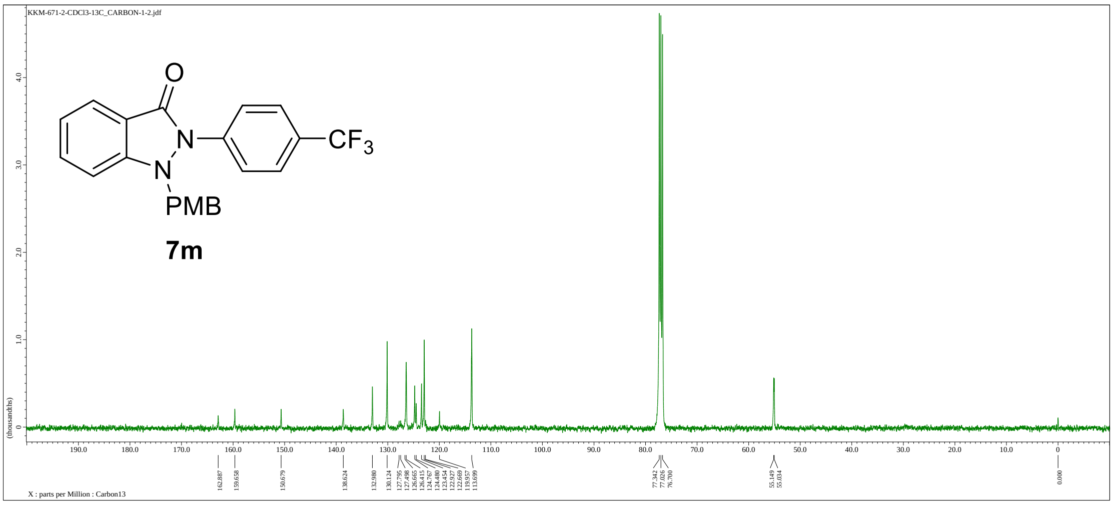

Figure S49. ^{1}H and ^{13}C NMR Spectra of Compounds **8m**.

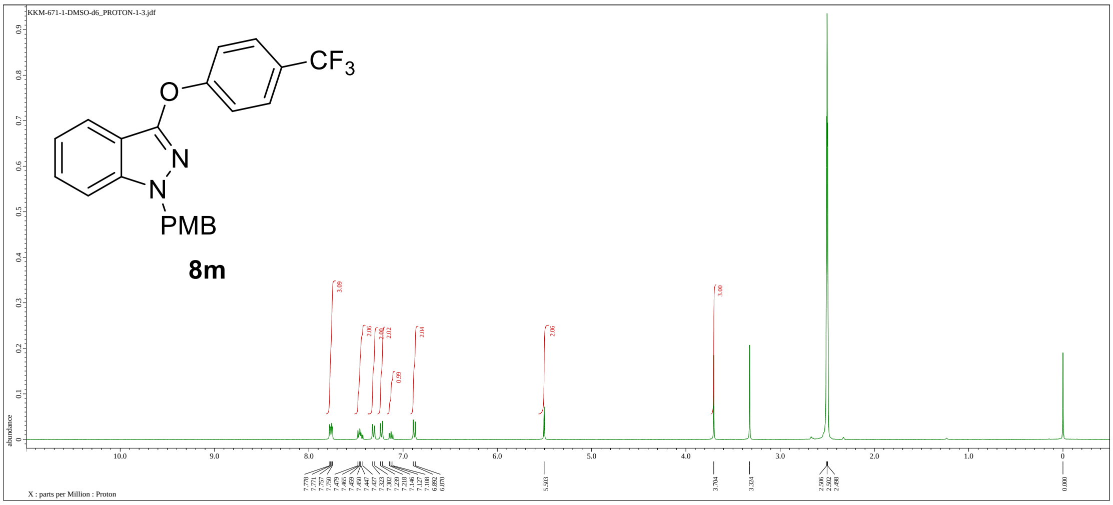

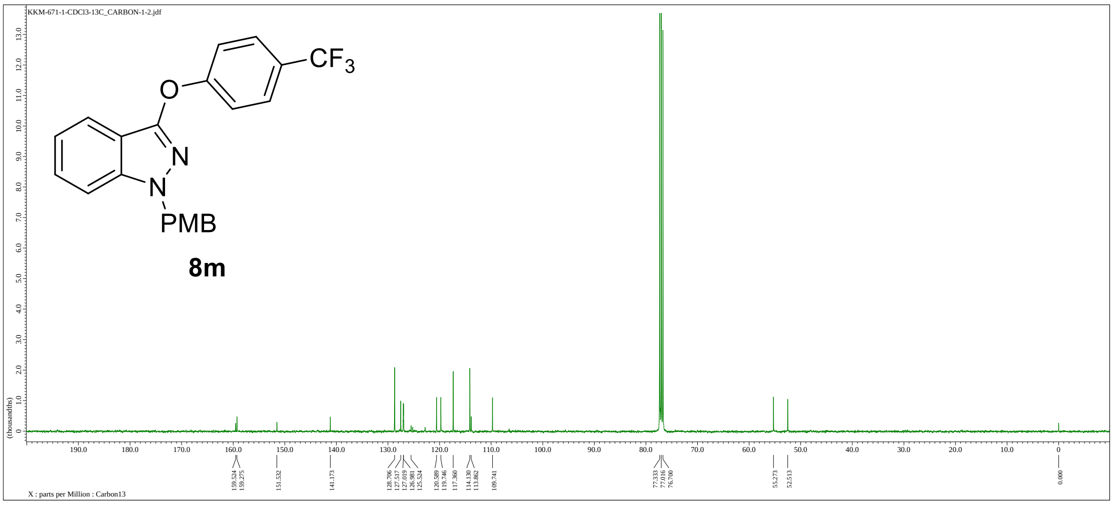

Figure S50. ^{1}H and ^{13}C NMR Spectra of Compounds **7n**.

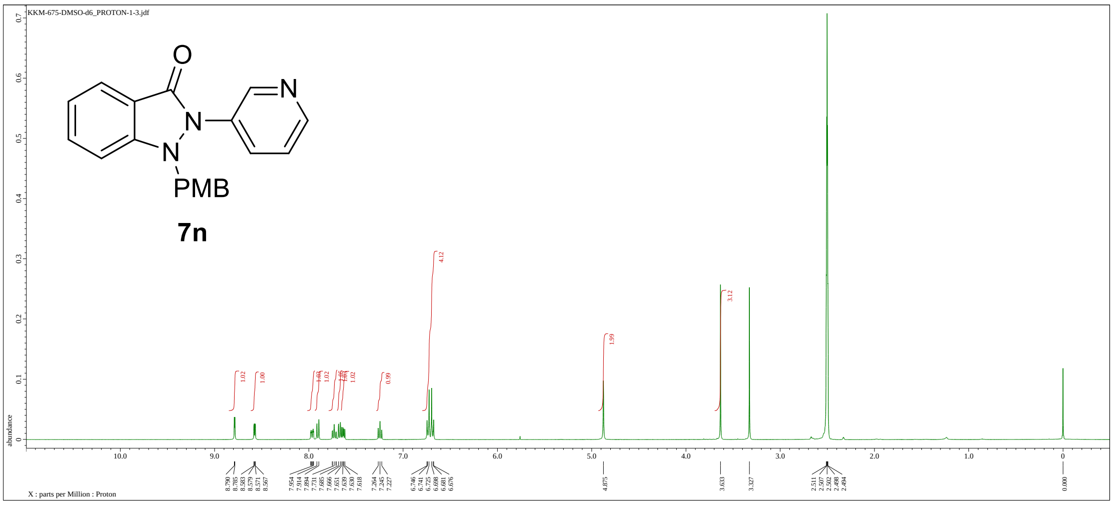

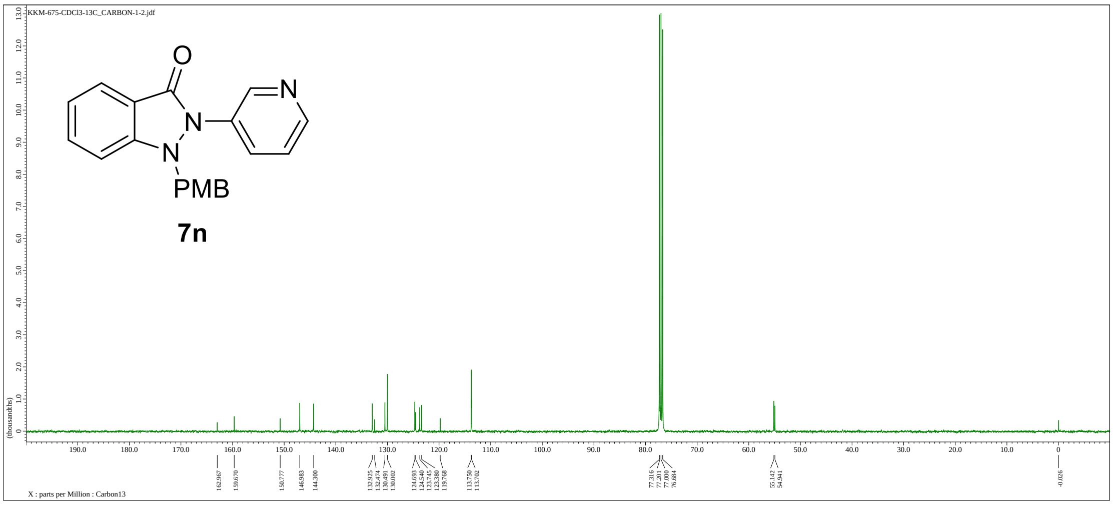

Figure S51. ^{1}H and ^{13}C NMR Spectra of Compounds **7o**.

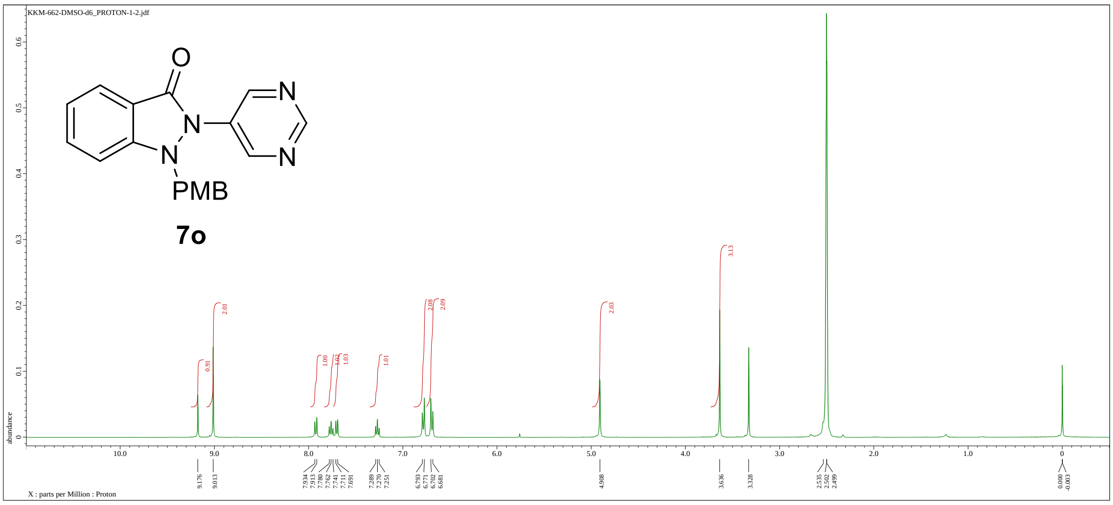

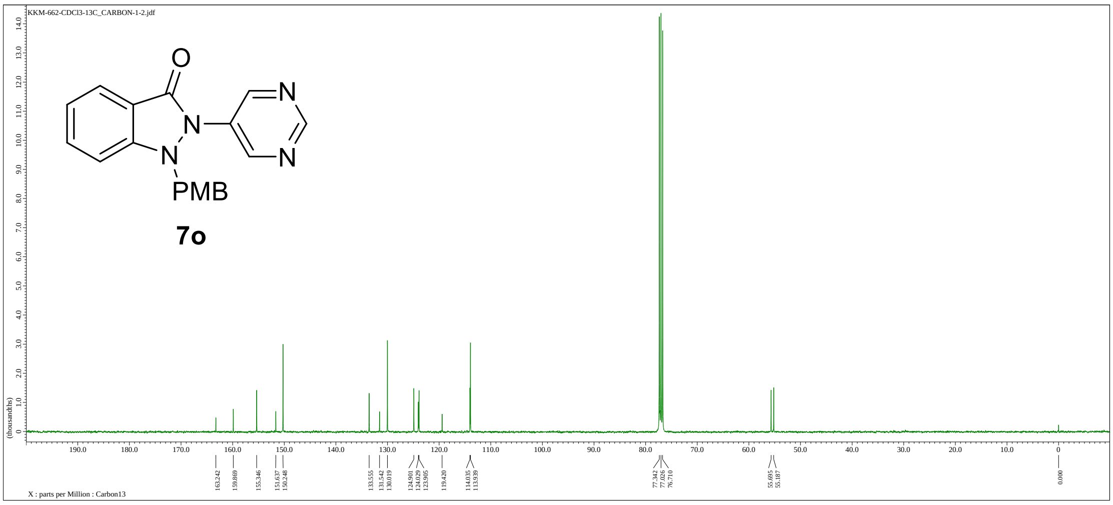

Figure S52. ^{1}H and ^{13}C NMR Spectra of Compounds **S1j**.

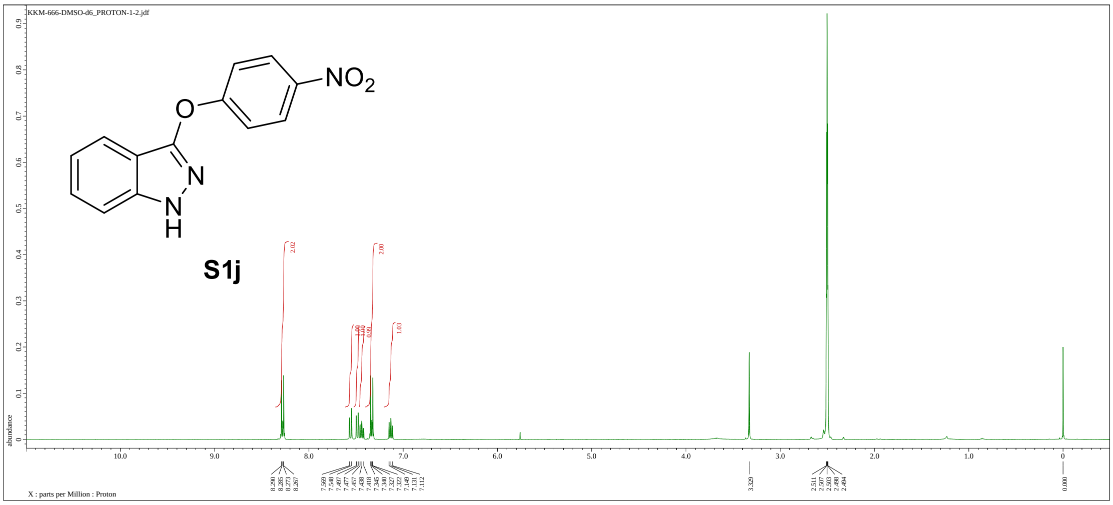

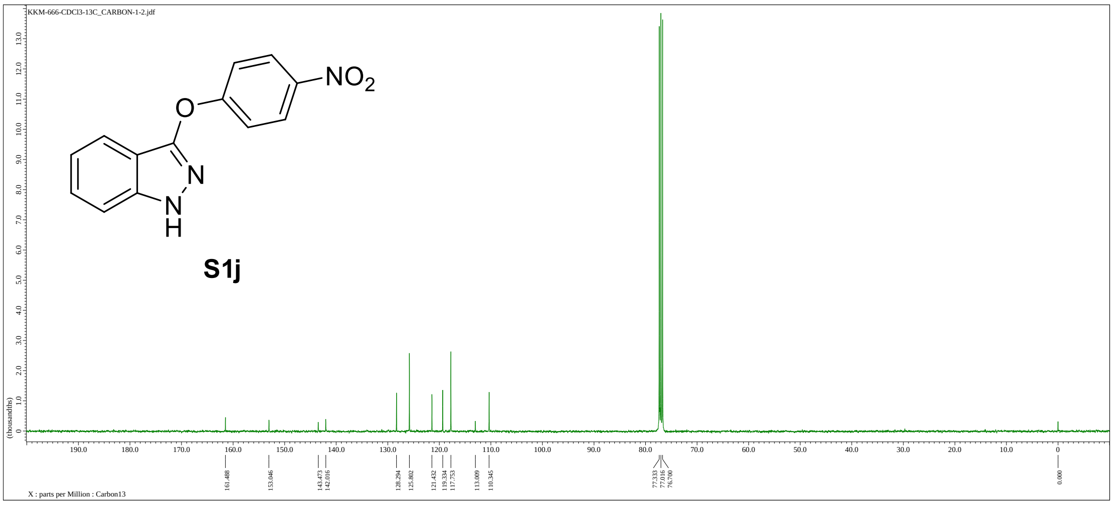

Figure S53. ^{1}H and ^{13}C NMR Spectra of Compounds **S1k**.

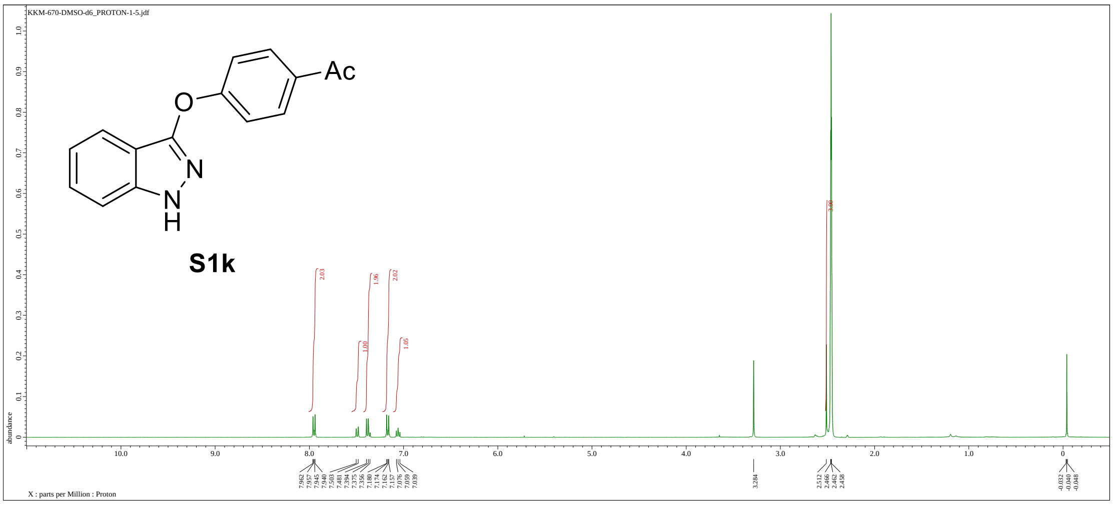

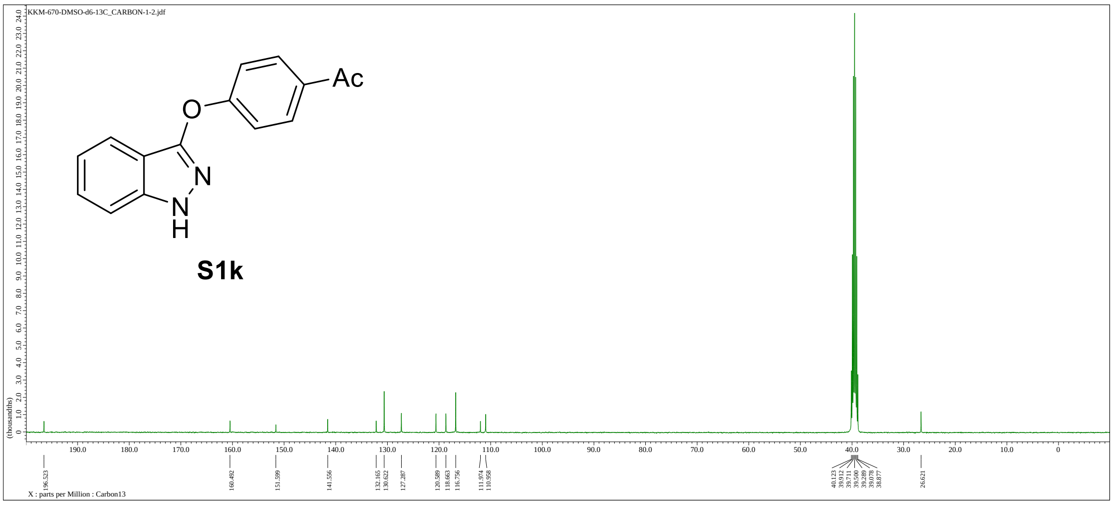

Figure S54. ^{1}H and ^{13}C NMR Spectra of Compounds **S1l**.

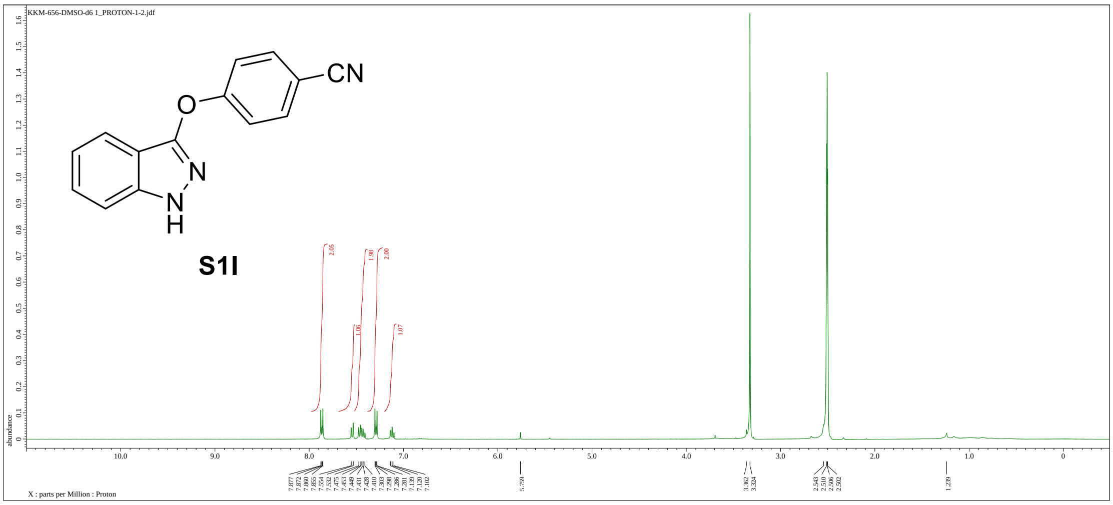

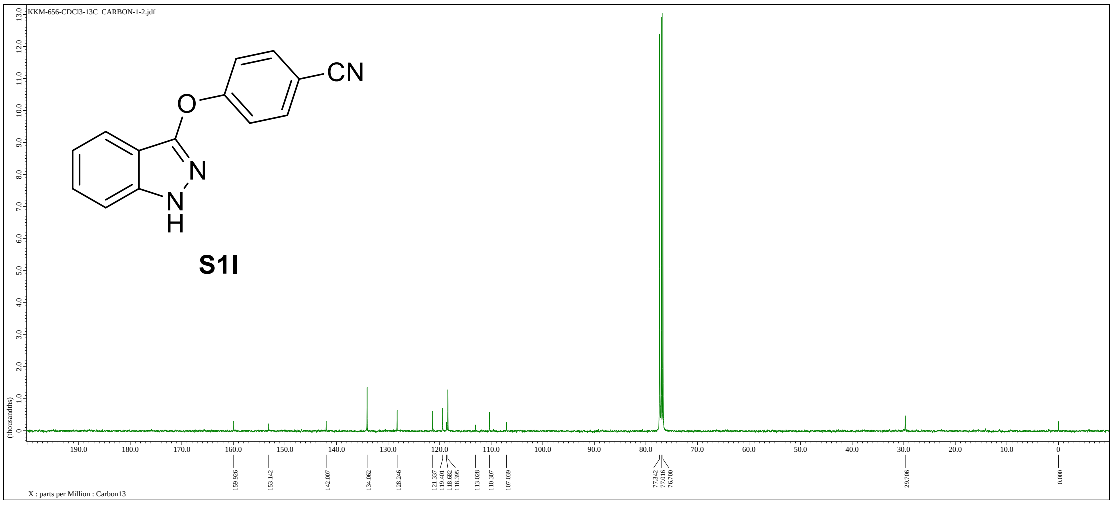

Figure S55. ^{1}H and ^{13}C NMR Spectra of Compounds **S1m**.

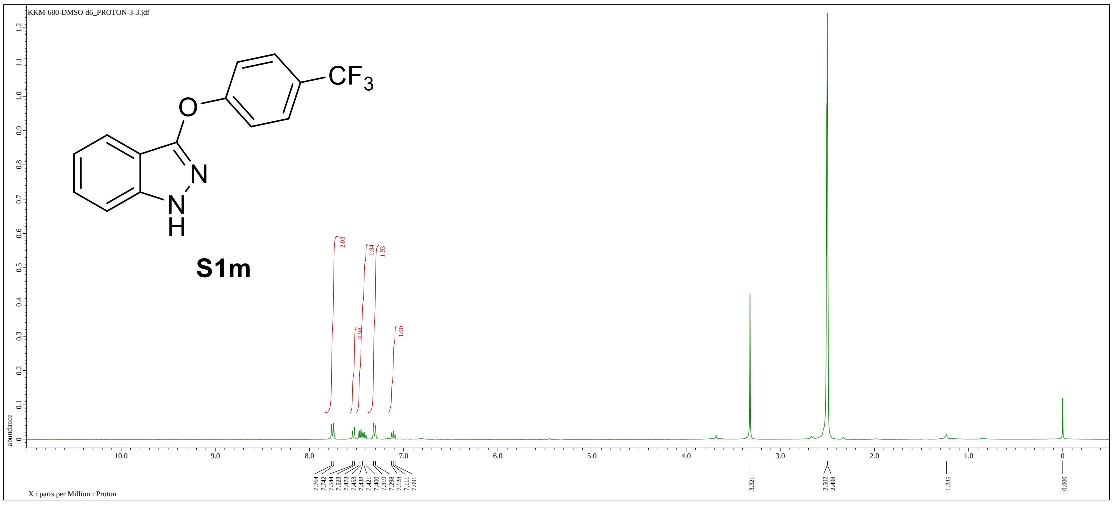

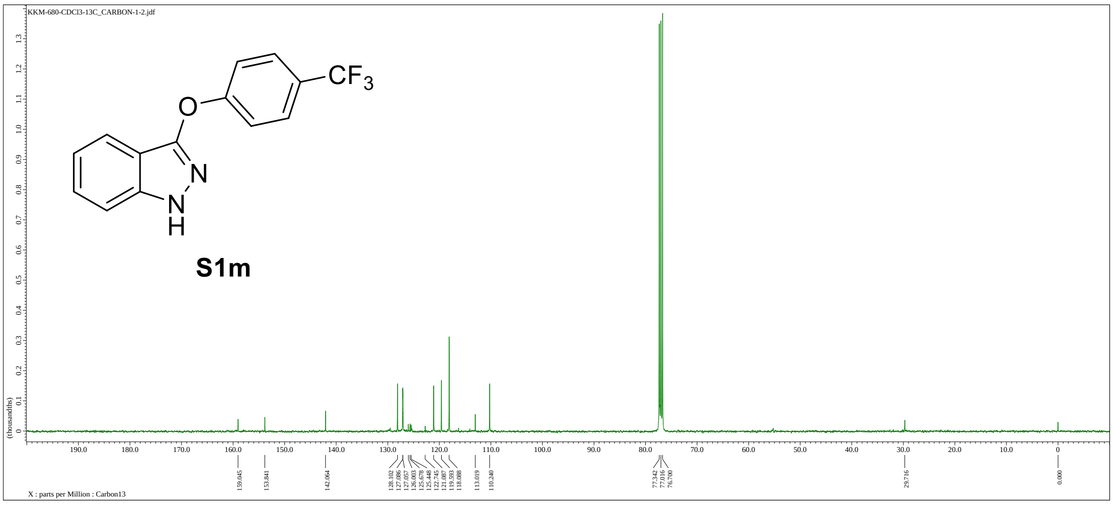

Supplement: Supplementary file 1 [file molecules-28-06706-s001.zip › molecules-2608421-supplementary.pdf]
